# Biomimetic dual-driven STING nanoagonist orchestrates neoadjuvant mild photo-immunotherapy for fibrosarcoma

**DOI:** 10.1016/j.mtbio.2026.103386

**Published:** 2026-06-23

**Authors:** Zhao Wang, Yuan Ma, Wangwei Zhang, Xiaoheng Dai, Xiao Zhou, Zhirong Zhang, Zhenhang Lin, Yilai Gao, Wei Zeng, Guohong Zhuang, Ting Wu, Wengang Li

**Affiliations:** a Department of Hepatobiliary Surgery, Xiang'an Hospital of Xiamen University, Cancer Research Center, School of Medicine, Xiamen University, Xiamen, Fujian, 361000, China; b Department of Pharmacy, The First Affiliated Hospital of Ningbo University, Ningbo, 315010, China; c Cancer Research Center, School of Medicine, Xiamen University, Xiamen, Fujian, 361000, China; d School of Life Sciences, Xiamen University, Xiamen, Fujian, 361000, China; e Department of Gastroenterology, Xiang'an Hospital of Xiamen University, School of Medicine, Xiamen University, Xiamen, Fujian, 361000, China; f Fujian Provincial Key Laboratory of Organ and Tissue Regeneration, School of Medicine, Organ Transplantation Institute of Xiamen University, Xiamen University, Xiamen, Fujian, 361000, China

**Keywords:** Dual-driven nanoagonist, STING activation, Mild photo-immunotherapy, Fibrosarcoma, Neoadjuvant therapy

## Abstract

Fibrosarcoma is an infiltrative malignant soft tissue sarcoma primarily managed with extensive surgical resection. However, securing adequate margins often compromises organ function, while postoperative recurrence and distant metastasis remain formidable clinical challenges, underscoring the urgent need for effective neoadjuvant therapeutic strategies. Neoadjuvant mild photo-immunotherapy represents a promising strategy for coupling preoperative tumor shrinkage with systemic immune priming. Notably, integrated analyses revealed relatively high expression of stimulator of interferon genes (STING) in fibrosarcoma, highlighting STING as a rational target for immunomodulation. Herein, we developed a biomimetic dual-driven STING nanoagonist for neoadjuvant mild photo-immunotherapy against fibrosarcoma. This nanoagonist comprises poly(lactic-co-glycolic acid) (PLGA) nanoparticles co-encapsulating the STING agonist 2′,3′-cyclic GMP-AMP (cGAMP) and the photosensitizer indocyanine green (ICG) (cGAMP/ICG@PLGA, GIP), which are further camouflaged with calreticulin (CRT)-enriched fibrosarcoma cell membranes (CRTM) to form GIP@CRTM. The biomimetic CRTM cloak retains tumor cell membrane antigens and CRT “eat-me” signals, promoting homotypic tumor accumulation and uptake by dendritic cells (DCs), thereby facilitating cGAMP delivery and dual-driven STING activation in both tumor cells and DCs. Upon near-infrared irradiation, ICG-mediated mild phototherapy induces moderate photothermal heating and reactive oxygen species (ROS) generation, triggering immunogenic cell death and further amplifying tumor-intrinsic STING signaling through ROS-induced DNA damage. This synergistic therapeutic effect enabled effective preoperative tumor shrinkage while eliciting systemic antitumor immunity to suppress postoperative contralateral rechallenge tumor outgrowth and limit distant tumor progression. Thus, this study establishes a STING-amplified mild photo-immunotherapeutic strategy, providing a potential neoadjuvant treatment option for fibrosarcoma.

## Graphical abstract

**Figure:**
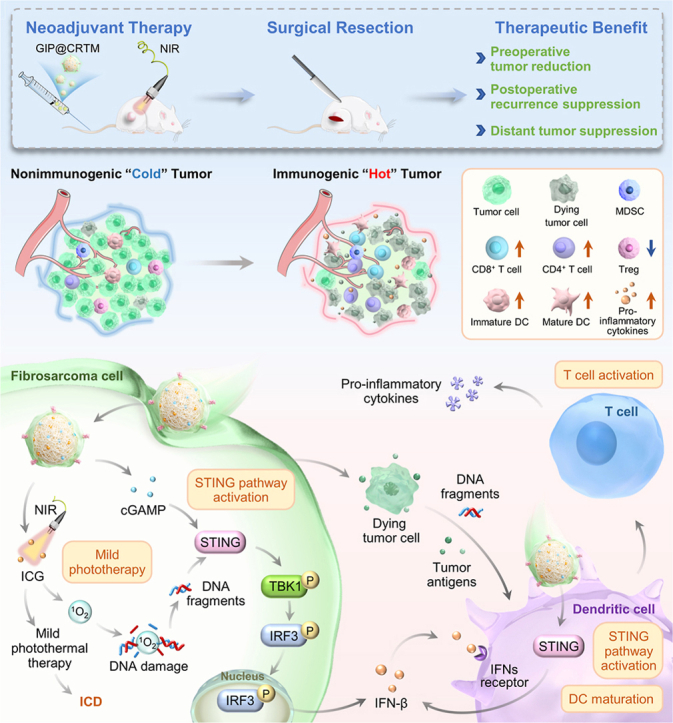

## 1 Introduction

Fibrosarcoma is a malignant mesenchymal soft tissue sarcoma for which wide surgical resection remains the current standard of care. However, these tumors are often large at diagnosis and exhibit infiltrative growth, making it challenging to achieve adequate surgical margins. Moreover, the wide excision required to obtain satisfactory margins may result in severe organ dysfunction. Consequently, surgery alone often fails to achieve an optimal balance between oncologic clearance and functional preservation. These limitations may be associated with an increased risk of postoperative recurrence and distant metastasis, ultimately compromising durable disease control. In addition, patients with fibrosarcoma may harbor occult micrometastatic disease at diagnosis, rendering local treatment alone insufficient to improve long-term outcomes [[1], [2], [3]]. As multidisciplinary cancer treatment paradigms have evolved, neoadjuvant therapy has emerged as an effective strategy for reducing preoperative tumor burden, improving resectability, increasing rates of margin-negative resection, and achieving systemic disease control, as demonstrated in multiple solid tumors [4,5]. Nevertheless, neoadjuvant strategies specifically for fibrosarcoma remain poorly explored. Therefore, exploring effective neoadjuvant treatment approaches for fibrosarcoma is of substantial importance for optimizing comprehensive treatment strategies and improving patient prognosis.

Preoperative immune activation has gained increasing attention as a neoadjuvant immunotherapeutic strategy because of its capacity to prime systemic antitumor immunity and establish sustained immune surveillance to mitigate postoperative recurrence [6,7]. Sarcoma has historically served as an important model in the early development of cancer immunotherapy, and epidemiological associations between sarcoma incidence and immune competence further suggest a role for immunomodulation in the control of this disease [8,9]. Stimulator of interferon genes (STING), a central adaptor in cytosolic DNA sensing, has emerged as a key target in cancer immunotherapy owing to its capacity to induce type I interferon production and initiate innate immune responses [10,11]. Notably, analyses of public databases revealed relatively high STING mRNA expression in sarcoma relative to other solid tumors, with levels that appeared higher than those observed in melanoma, a canonical model for STING agonist studies [[12], [13], [14]]. Additionally, STING mRNA expression was substantially elevated in sarcoma tissues compared with adjacent normal counterparts (Fig. S1). Consistent with these bioinformatic findings, the fibrosarcoma cell line WEHI-164 exhibited higher STING mRNA and protein levels than the melanoma cell line B16F10 (Fig. S2). Together, these findings suggest that fibrosarcoma, a representative sarcoma subtype, exhibits considerable STING-associated immunomodulatory potential, thereby providing a biological rationale for the development of STING-based neoadjuvant immunotherapy.

As a neoadjuvant strategy, phototherapy provides rapid and localized preoperative tumor reduction that is difficult to achieve with immunotherapy alone [15,16]. Photothermal therapy and photodynamic therapy represent two complementary phototherapeutic modalities [[17], [18], [19]]. Photothermal therapy achieves tumor ablation by converting light energy into localized heat [20], and clinical evidence suggests that local hyperthermia significantly prolongs survival in patients with sarcoma [21,22]. However, conventional hyperthermia (>50 °C) often results in uncontrolled heat diffusion, causing nonspecific tissue injury [23]. By contrast, mild photothermal therapy (42-45 °C) enables safe tumor control while avoiding excessive heat-induced antigen denaturation and is therefore frequently combined with immunotherapy to enhance therapeutic efficacy [[24], [25], [26]]. Furthermore, photodynamic therapy generates reactive oxygen species (ROS) via light-activated photosensitizers, inducing DNA damage that activates tumor-intrinsic STING signaling and amplifies innate and adaptive immune responses [[27], [28], [29]]. In situ ROS-generating therapies are widely regarded as effective synergistic partners for STING-mediated immunotherapy [30,31]. Collectively, by engaging complementary mechanisms, the combination of mild phototherapy and STING-based immunotherapy may synergistically enhance neoadjuvant therapeutic efficacy in fibrosarcoma.

With continued advances in nanomedicine, nanomaterials have been increasingly explored in neoadjuvant cancer therapy, offering new opportunities for optimizing preoperative treatment strategies [32,33]. Owing to their structural tunability and capacity for multifunctional integration, nanodelivery systems can improve drug stability and tumor accumulation in vivo, thereby alleviating key limitations of conventional free drugs, including rapid clearance, suboptimal biodistribution, and dose-limiting systemic toxicity [[34], [35], [36], [37]]. Importantly, nanoplatforms enable the coordinated integration of therapeutic modalities with distinct mechanisms of action, thereby enhancing therapeutic synergy and overall treatment outcomes [[38], [39], [40], [41], [42]]. In this context, rational nanocarrier design offers a feasible and controllable approach to implementing neoadjuvant mild photo-immunotherapeutic intervention in fibrosarcoma.

**Scheme 1:**
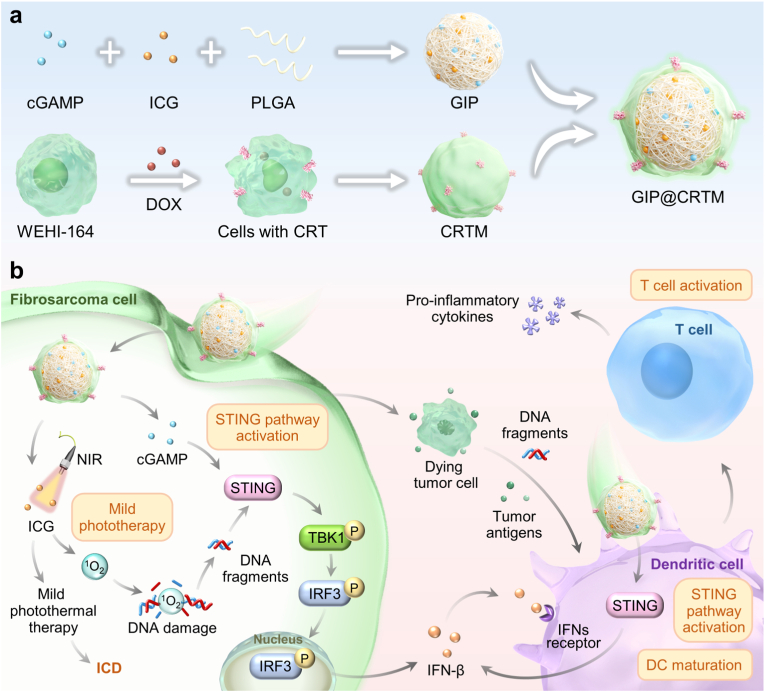
**Schematic illustration of the fabrication and functional mechanisms of the GIP@CRTM nanoagonist.** (a) Preparation process and structural features of GIP@CRTM. (b) Mechanism of GIP@CRTM-mediated mild photo-immunotherapy against fibrosarcoma.

In this study, we developed a biomimetic dual-driven STING nanoagonist for neoadjuvant mild photo-immunotherapy in fibrosarcoma. Here, “dual-driven” denotes the coordinated activation of STING signaling in both tumor cells and dendritic cells (DCs), two pivotal cell types involved in initiating and amplifying antitumor immunity. The system consists of poly(lactic-co-glycolic acid) (PLGA) nanoparticles co-encapsulating the STING agonist 2′,3′-cyclic GMP-AMP (cGAMP) and the photosensitizer indocyanine green (ICG) (cGAMP/ICG@PLGA, GIP), which are further cloaked with calreticulin (CRT)-enriched fibrosarcoma cell membranes (CRTM) to form GIP@CRTM (Scheme 1a). The biomimetic CRTM coating, derived from doxorubicin (DOX)-pretreated fibrosarcoma cells, retains a broad profile of tumor cell membrane antigens and abundant CRT “eat-me” signals, thereby promoting homologous tumor accumulation and internalization by DCs. Consequently, GIP@CRTM enables dual-driven activation of STING signaling in both tumor cells and DCs, enhancing antitumor immune activation. Upon near-infrared (NIR) irradiation, ICG-mediated mild phototherapy induces mild photothermal heating (42-45 °C) and ROS generation. This dual photonic input promotes immunogenic cell death (ICD) to heighten tumor immunogenicity and triggers ROS-driven DNA damage that amplifies tumor-intrinsic STING signaling (Scheme 1b). By integrating localized physical tumor ablation with STING pathway activation, this strategy reduces primary tumor burden to improve surgical resectability and can elicit systemic antitumor immunity and durable immune memory, thereby suppressing distant progression and postoperative contralateral rechallenge tumor outgrowth.

Several recent studies have also explored nanotherapeutic strategies integrating STING activation with phototherapy [[43], [44], [45], [46]]. These studies have demonstrated the feasibility of combining STING activation with phototherapy. However, most existing strategies have primarily been designed to enhance the delivery of STING agonists to tumor cells and to amplify tumor cell-associated STING signaling. In contrast, GIP@CRTM incorporates CRT-enriched homologous tumor cell membranes as a biomimetic interface. This design not only preserves homologous tumor-targeting capability but also enhances uptake by DCs through CRT-mediated “eat-me” signals, thereby extending STING activation from a tumor cell-centered mode to a dual-cell-driven mode involving both tumor cells and DCs. In addition, ICG-mediated mild phototherapy is not merely employed to induce ICD but also further amplifies cGAMP-mediated STING activation through ROS-associated DNA damage. More importantly, unlike most conventional photo-immunotherapy studies, this strategy was evaluated within a neoadjuvant treatment framework for fibrosarcoma, and its effects on preoperative tumor shrinkage, suppression of postoperative contralateral rechallenge tumor outgrowth, distant lesion control, and the establishment of immune memory were systematically assessed. Therefore, GIP@CRTM is clearly distinguished from existing STING-phototherapy nanoplatforms and provides a more targeted biomimetic synergistic strategy for neoadjuvant photo-immunotherapy in fibrosarcoma. Overall, GIP@CRTM-based mild photo-immunotherapy represents a rational neoadjuvant strategy for improving surgical feasibility and long-term therapeutic outcomes in fibrosarcoma.

## 2 Results and discussion

### 2.1 In vitro pretreatment of WEHI-164 cells with DOX

**Fig. 1:**
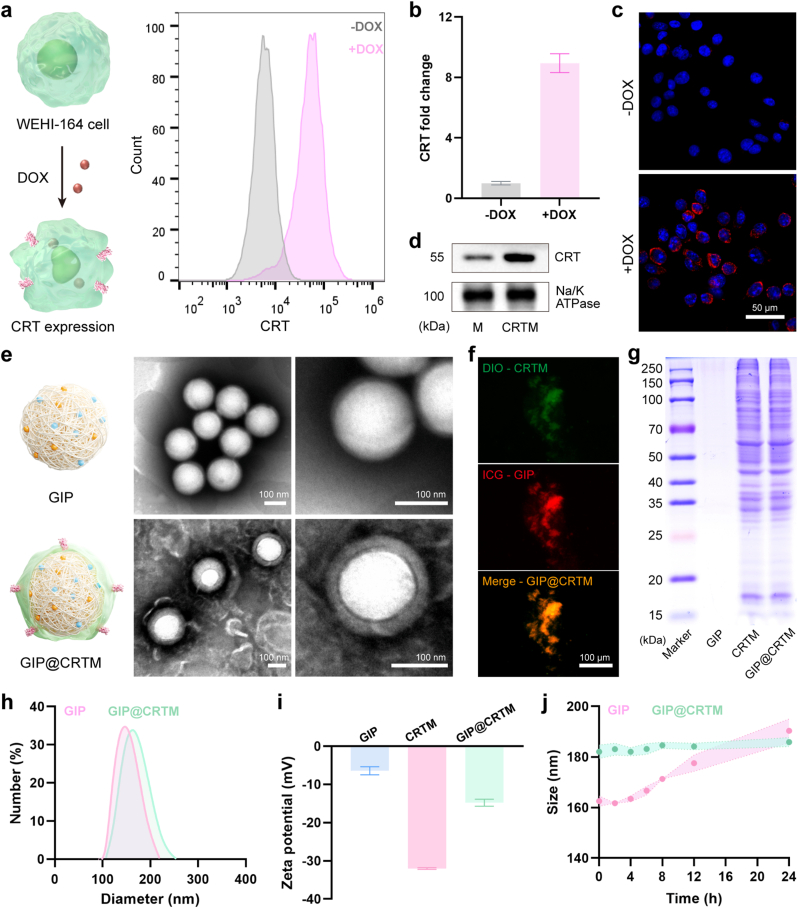
**Preparation and characterization of GIP@CRTM.** (a) Flow cytometric plots and (b) relative quantification of CRT exposure on WEHI-164 cell membranes after 24 h of incubation with 2.5 μM DOX. (c) CLSM images showing CRT (red) on the WEHI-164 cell surfaces before and after DOX treatment, with nuclei stained by DAPI (blue). Scale bar = 50 μm. (d) Western blot analysis of CRT expression in WEHI-164 cell membranes before (M) and after (CRTM) DOX treatment. (e) TEM images of GIP and GIP@CRTM. Scale bar = 100 nm. (f) Fluorescence images of GIP@CRTM (red: ICG in PLGA; green: DiO-labeled cell membranes). Scale bar = 100 μm. (g) SDS-PAGE profiles of GIP, CRTM, and GIP@CRTM. (h) Dynamic light scattering (DLS) analysis of GIP and GIP@CRTM. (i) Zeta potential of GIP, CRTM, and GIP@CRTM. (j) Stability of GIP and GIP@CRTM in PBS over time by DLS. Data were presented as mean ± standard deviation (SD) (n = 3). (For interpretation of the references to colour in this figure legend, the reader is referred to the Web version of this article.)

To obtain CRT-enriched WEHI-164 cell membranes for subsequent biomimetic coating, WEHI-164 cells were pretreated with DOX in vitro to induce CRT externalization [47,48]. The effects of DOX concentration and exposure duration on cell surface CRT expression were systematically evaluated. As shown in Fig. S3, after 12 h of exposure, 2.5 μM DOX treatment significantly upregulated cell surface CRT expression while maintaining high cell viability, which is important for preserving membrane functionality (Fig. S4). Extending the exposure to 24 h at the same concentration further enhanced CRT externalization (Fig. S5, Fig. 1a). Quantitative analysis revealed an 8.94-fold increase in surface CRT expression compared with untreated cells (Fig. 1b), while cell viability remained above 70% (Fig. S6). Immunofluorescence staining corroborated the pronounced CRT enrichment on the cell surface under these conditions (Fig. 1c). Therefore, pretreatment with 2.5 μM DOX for 24 h was identified as the optimal condition for inducing robust CRT externalization in WEHI-164 cells and was selected for subsequent biomimetic nanoplatform fabrication.

### 2.2 Characterization of the nanoagonist

To construct GIP@CRTM, CRTM was isolated and purified from DOX-pretreated WEHI-164 cells by mechanical disruption followed by differential centrifugation. As a control, cell membranes derived from untreated WEHI-164 cells (M) were prepared using the same procedure. Western blot analysis confirmed markedly higher CRT expression in CRTM than in M (Fig. 1d, Fig. S7). Next, CRTM was fused with GIP through ultrasonication and membrane extrusion to obtain the final GIP@CRTM formulation. In parallel, M was fused with GIP using the same procedure to obtain GIP@M for subsequent control experiments.

Transmission electron microscopy (TEM) images revealed that the synthesized nanoparticles were monodisperse and exhibited a spherical morphology. GIP@CRTM exhibited a distinct core-shell structure, indicating successful membrane coating (Fig. 1e). Membrane cloaking was further confirmed by confocal laser scanning microscopy (CLSM), where colocalization of DiO-labeled CRTM (green) and ICG encapsulated within the PLGA core (red) generated a merged yellow signal (Fig. 1f). To further quantify the CRTM coating efficiency, nano-flow cytometric analysis was performed by simultaneously detecting the DiO-labeled membrane signal and the intrinsic ICG fluorescence of the GIP core (Fig. S8). DiO/ICG double-positive events were defined as GIP nanoparticles coated with CRTM. The results showed that the membrane coating efficiency of GIP@CRTM was approximately 62.7%. In addition, SDS-PAGE analysis showed that bare GIP lacked protein components, whereas GIP@CRTM exhibited protein profiles that were highly similar to those of CRTM, suggesting that the membrane coating process preserved the native membrane protein composition and enabled GIP@CRTM to retain the protein features and potential biological functions of CRTM (Fig. 1g). After membrane coating, the hydrodynamic diameter increased from 163.5 nm to 181.4 nm (Fig. 1h), while the zeta potential shifted from −6.41 mV to −14.8 mV (Fig. 1i). The polydispersity index (PDI) values of freshly prepared GIP and GIP@CRTM were approximately 0.18 and 0.14, respectively, suggesting that both formulations possessed relatively narrow size distributions and good particle uniformity (Fig. S9). GIP@CRTM exhibited efficient drug-loading capacity, with encapsulation efficiencies of approximately 60.6% for cGAMP and 75.3% for ICG, as determined by HPLC and UV-vis spectroscopy. Accordingly, the drug loading contents of ICG and cGAMP were calculated to be 5.86% and 36.41%, respectively, corresponding to a total drug loading content of 42.27%. The actual mass ratio of encapsulated ICG to cGAMP was approximately 6.21:1, and this ratio was maintained throughout all in vitro and in vivo experiments. Drug release behavior was further assessed using buffers at pH 5.0 and pH 7.4 to simulate lysosomal and physiological environments, respectively. GIP@CRTM displayed accelerated release under acidic lysosomal conditions, suggesting that this design may help reduce premature drug leakage during circulation and improve drug bioavailability (Fig. S10). Although GIP showed a slightly faster release profile than GIP@CRTM under identical conditions, it exhibited poorer colloidal stability in PBS. The hydrodynamic diameter of GIP gradually increased over 24 h, with its PDI rising from approximately 0.18 to 0.41, indicating particle aggregation and a broadened size distribution. In contrast, GIP@CRTM maintained a nearly unchanged hydrodynamic diameter, and its PDI remained below 0.3 throughout the observation period. This improved stability was likely attributable to the negatively charged cell membrane coating, which enhanced nanoparticle dispersibility and reduced the aggregation tendency of GIP@CRTM (Fig. 1j, Fig. S11). Moreover, cytotoxicity and hemolysis assays (Figs. S12 and S13) confirmed the favorable biocompatibility of GIP@CRTM, underscoring its potential for both in vitro and in vivo applications.

### 2.3 In vitro antitumor activity and STING pathway activation in tumor cells

**Fig. 2:**
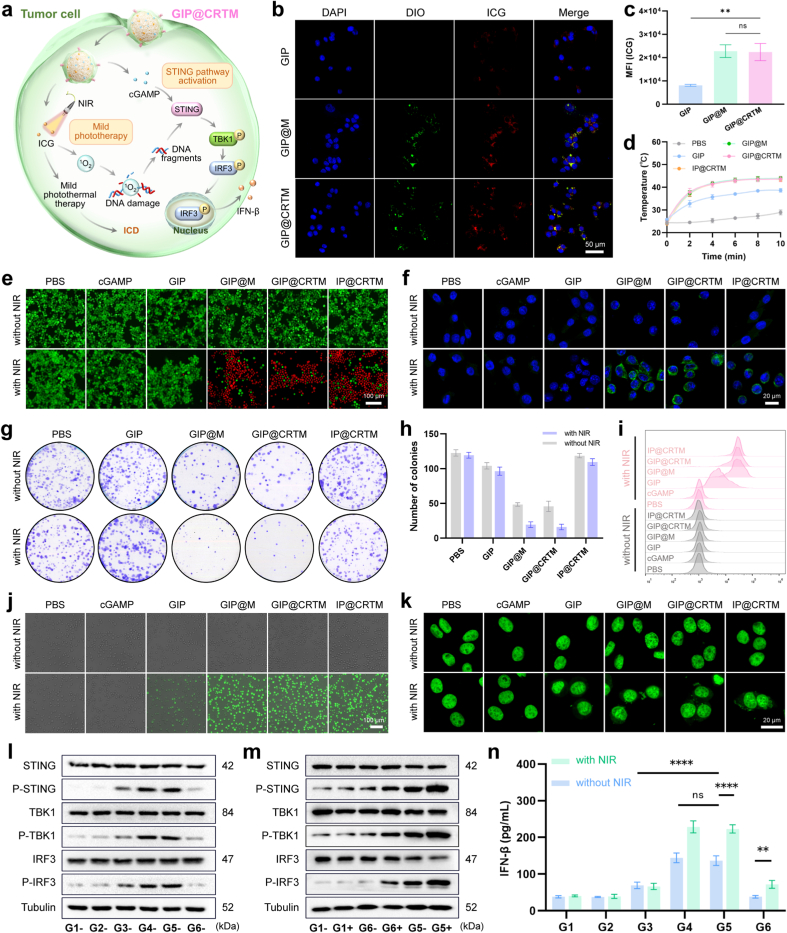
**In vitro antitumor effects and STING pathway activation in tumor cells induced by GIP@CRTM.** G1: PBS, G2: cGAMP, G3: GIP, G4: GIP@M, G5: GIP@CRTM, G6: IP@CRTM. (+): tumor cells with NIR irradiation (808 nm, 0.5 W/cm^2^, 8 min), (−): tumor cells without NIR irradiation. (a) Schematic illustration of the effects of GIP@CRTM on tumor cells. (b) Confocal images and (c) flow cytometric analysis of WEHI-164 cells incubated with GIP, GIP@M, or GIP@CRTM for 6 h. Blue, DAPI; green, DiO; red, ICG. Scale bar = 50 μm. (d) Temperature elevation of WEHI-164 cells incubated with PBS, GIP, GIP@M, GIP@CRTM, or IP@CRTM under NIR irradiation. (e) Calcein AM/PI (live/dead) staining. Scale bar = 100 μm. (f) CLSM images of CRT exposure on WEHI-164 cell surfaces. Scale bar = 20 μm. (g) Colony formation of WEHI-164 cells after different treatments and (h) quantification of colony numbers. (i) Flow cytometric plots and (j) CLSM images of intracellular ROS generation. Scale bar = 100 μm. (k) Representative images of PicoGreen-stained WEHI-164 cells. Scale bar = 20 μm. (l) Western blot of STING pathway activation in WEHI-164 cells treated with different nanoagonists without NIR irradiation. (m) Western blot analysis of STING pathway activation in WEHI-164 cells treated with PBS, GIP@CRTM, or IP@CRTM with or without NIR irradiation. (n) IFN-β secretion in cell supernatants under various treatments. Data were shown as mean ± SD (n = 3). ns, *p* > 0.05; **p < 0.01; ****p < 0.0001. (For interpretation of the references to colour in this figure legend, the reader is referred to the Web version of this article.)

Owing to the homotypic affinity conferred by tumor cell membrane coating [49], membrane-cloaked STING nanoagonists were efficiently internalized by homologous tumor cells, thereby facilitating STING activation in tumor cells. Upon NIR irradiation, the encapsulated ICG generated mild photothermal stress and ROS, inducing ICD and ROS-mediated DNA damage, thereby further amplifying STING pathway activation in tumor cells (Fig. 2a).

To validate the proposed homotypic recognition and cellular uptake mechanism, we first evaluated the selective uptake of GIP@CRTM by homologous WEHI-164 fibrosarcoma cells and normal cells, including AML12 hepatocytes and L929 fibroblasts. Flow cytometric analysis showed that markedly stronger ICG fluorescence was detected in WEHI-164 cells after GIP@CRTM treatment than in AML12 and L929 cells, whereas only weak fluorescence signals were observed in the two normal cell types (Fig. S14a). Consistently, confocal fluorescence imaging revealed pronounced red ICG fluorescence in WEHI-164 cells, while much weaker signals were detected in AML12 and L929 cells (Fig. S14b). These findings indicate that GIP@CRTM was preferentially taken up by homologous WEHI-164 tumor cells, with relatively low nonspecific uptake in the tested normal cells, thereby supporting the role of CRTM coating in mediating homotypic recognition.

After confirming this homotypic recognition capability, we further evaluated the uptake efficiency of different nanoagonist formulations in WEHI-164 cells. Fluorescence microscopy (Fig. 2b) and flow cytometry (Fig. 2c, Fig. S15) showed that both GIP@M and GIP@CRTM exhibited comparably high uptake by WEHI-164 cells, markedly exceeding that of unmodified GIP. These results indicate that membrane modification preserved the homotypic recognition capability of tumor cell membranes. Moreover, cellular uptake of GIP@CRTM increased in a time-dependent manner and reached saturation at approximately 6 h (Fig. S16). Based on these findings, WEHI-164 cells were incubated with GIP@CRTM for 6 h and subsequently subjected to NIR laser irradiation to assess photothermal responsiveness. To achieve mild photothermal conditions, irradiation at 0.5 W/cm^2^ gradually increased the cellular temperature, maintaining it within the therapeutic range of 42-45 °C (Fig. S17). Under identical conditions, GIP@CRTM, GIP@M, and IP@CRTM (lacking cGAMP) all reached comparable temperatures within the mild photothermal range, with no significant differences observed among these groups. By contrast, GIP exhibited poor cellular uptake, resulting in insufficient heating that failed to reach 42 °C (Fig. 2d, Fig. S18).

To further evaluate the in vitro therapeutic efficacy of the nanoagonists, live/dead staining (Fig. 2e) and CCK-8 assays (Fig. S19) were performed to assess tumor cell viability under different treatments. In the absence of irradiation, tumor cells in all treatment groups showed no apparent cell death; however, GIP@M and GIP@CRTM both induced approximately 15% growth inhibition, which was greater than that induced by GIP (6.8%), indicating that STING nanoagonists alone exert a modest antiproliferative effect. Upon NIR irradiation, GIP@M, GIP@CRTM, and IP@CRTM all induced pronounced tumor cell death, accompanied by elevated ICD marker levels (Fig. 2f, Fig. S20). Mild phototherapy alone (IP@CRTM) reduced cell viability to 39.7%, whereas either GIP@M or GIP@CRTM combined with irradiation further decreased viability to approximately 15%. Furthermore, long-term proliferative capacity was evaluated by colony formation assays. First, free cGAMP, either alone or combined with NIR irradiation, did not significantly affect the colony-forming ability of WEHI-164 cells (Fig. S21). This result may be attributable to the poor membrane permeability and limited intracellular delivery efficiency of free cGAMP. GIP@CRTM inhibited colony formation by 62.8% even without irradiation, whereas mild phototherapy alone (IP@CRTM) suppressed colony formation by 10.9%. Combination treatment with GIP@CRTM and NIR irradiation further reduced clonogenic survival, resulting in 87.0% inhibition of colony formation. GIP@M exhibited long-term inhibitory effects comparable to those of GIP@CRTM. In contrast, GIP exhibited no significant difference in therapeutic efficacy with or without phototherapy, likely due to limited cellular uptake and insufficient intracellular accumulation of ICG (Fig. 2g and h). Overall, STING nanoagonists camouflaged with cell membranes (GIP@M and GIP@CRTM) achieved stronger in vitro antitumor effects than unmodified GIP, and mild phototherapy provided additional synergistic enhancement, potentially through amplified activation of the STING pathway.

In principle, any mechanism that causes DNA damage and promotes DNA leakage into the cytosol may enhance STING pathway activation [[50], [51], [52]]. Upon laser irradiation, ICG induces photodynamic effects, generating ROS that cause DNA damage and promote DNA release into the cytosol. To validate this process, intracellular ROS accumulation and DNA leakage were assessed. Upon irradiation, GIP@M, GIP@CRTM, and IP@CRTM significantly increased ROS levels in tumor cells (Fig. 2i, j and Fig. S22). PicoGreen staining (Fig. 2k) revealed prominent cytosolic green fluorescent puncta, indicating substantial DNA fragment leakage. To further verify cytosolic mitochondrial DNA accumulation after irradiation, qPCR analysis of cytosolic fractions was performed using the mitochondrially encoded genes mt-Nd1 and mt-Cox1 as mitochondrial DNA markers (Fig. S23). Consistent with the PicoGreen staining results, markedly increased cytosolic levels of mt-Nd1 and mt-Cox1 were observed following NIR irradiation in the GIP@M, GIP@CRTM, and IP@CRTM groups. The concomitant elevation in ROS generation suggests that NIR-triggered ROS production promoted cytosolic mitochondrial DNA accumulation in WEHI-164 tumor cells, thereby providing an additional DNA source for subsequent STING pathway activation.

To assess STING pathway activation in tumor cells after treatment, key signaling proteins were examined by Western blotting. In the absence of irradiation, GIP (G3-) induced only a slight increase in the levels of phosphorylated STING (P-STING), phosphorylated TANK-binding kinase 1 (P-TBK1), and phosphorylated interferon regulatory factor 3 (P-IRF3). By contrast, GIP@M (G4-) and GIP@CRTM (G5-) elicited more pronounced phosphorylation of these proteins, indicating stronger activation of the STING pathway relative to GIP-treated tumor cells (Fig. 2l, Fig. S24). Crucially, mild phototherapy (G6+) moderately activated STING through DNA damage. Compared with GIP@CRTM (G5-), the combined treatment (G5+) further enhanced STING pathway activation (Fig. 2m, Fig. S25). Consistently, ELISA results (Fig. 2n, Fig. S26) confirmed that GIP@M (G4-) and GIP@CRTM (G5-) significantly enhanced the secretion of downstream STING-related cytokines, including interferon-β (IFN-β), interleukin-6 (IL-6), and C-X-C motif chemokine ligand 10 (CXCL10). When combined with phototherapy, cytokine production was further amplified, contributing to a robust proinflammatory milieu conducive to subsequent antitumor immune responses.

### 2.4 In vitro DC maturation and STING pathway activation

**Fig. 3:**
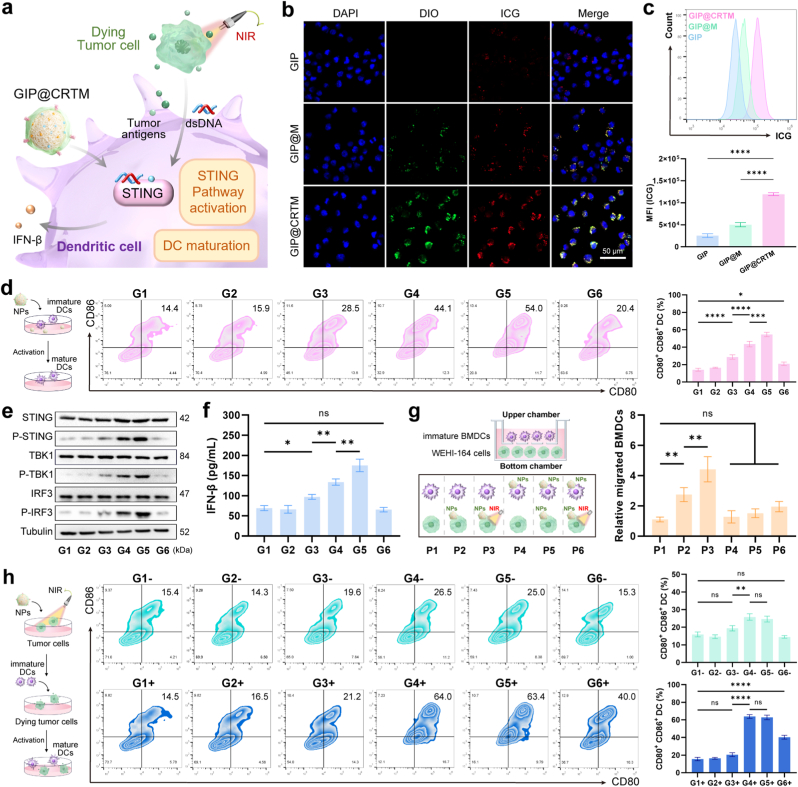
**BMDC uptake and activation induced by GIP@CRTM.** G1: PBS, G2: cGAMP, G3: GIP, G4: GIP@M, G5: GIP@CRTM, G6: IP@CRTM. (+): tumor cells with NIR irradiation (808 nm, 0.5 W/cm^2^, 8 min), (−): tumor cells without NIR irradiation. (a) Schematic illustration of intracellular activation of BMDCs by GIP@CRTM. (b) Confocal images and (c) flow cytometric analysis of BMDCs incubated with GIP, GIP@M, or GIP@CRTM for 4 h. Blue, DAPI; green, DiO; red, ICG. Scale bar = 50 μm. (d) Flow cytometric analysis of BMDC activation following nanoagonist treatment. (e) Western blot analysis of STING pathway-related proteins in BMDCs after different treatments. (f) IFN-β secretion from BMDCs treated with nanoagonists. (g) Transwell assay of BMDC migration toward tumor cells subjected to different treatments. (h) Flow cytometric analysis of BMDC activation after co-culture with tumor cells under different treatments. Values were shown as mean ± SD (n = 3). ns, *p* > 0.05; *p < 0.05; **p < 0.01; ***p < 0.001; ****p < 0.0001. (For interpretation of the references to colour in this figure legend, the reader is referred to the Web version of this article.)

As an “eat-me” signal, CRT is known to promote uptake by DCs [53], thereby enhancing the uptake of GIP@CRTM by DCs. By simultaneously delivering the STING agonist and tumor membrane antigens, GIP@CRTM is expected to facilitate DC recruitment and activation. In addition, mild photo-immunotherapy induces tumor cell death, leading to the release of tumor antigens and cytosolic DNA that may further stimulate DC activation and support subsequent immune responses (Fig. 3a).

Efficient internalization by DCs is a critical prerequisite for effective immunostimulation. Fluorescence microscopy (Fig. 3b) and flow cytometry (Fig. 3c) demonstrated that uptake of GIP@CRTM by BMDCs was 2.38-fold higher than that of GIP@M and 4.68-fold higher than that of GIP, highlighting the positive contribution of CRTM to BMDC internalization. Flow cytometric analysis further showed that GIP@CRTM (G5) increased BMDC maturation to 54.6%, compared with 28.7% for GIP (G3) and 43.5% for GIP@M (G4), corresponding to 1.90-fold and 1.26-fold increases, respectively. IP@CRTM (G6, 20.9%) induced only a modest maturation effect relative to untreated controls (G1, 14.0%) (Fig. 3d). Western blot analysis demonstrated that IP@CRTM (G6) failed to activate the STING pathway in BMDCs. GIP@CRTM (G5) induced higher levels of P-STING, P-TBK1, and P-IRF3 in BMDCs than GIP (G3) and GIP@M (G4), confirming stronger STING activation in BMDCs (Fig. 3e, Fig. S27). Elevated secretion of IFN-β and other downstream STING-related cytokines further supported this activation profile (Fig. 3f, Fig. S28). These results indicate that IP@CRTM induced only modest DC activation, likely because it carries only limited amounts of membrane-associated antigens, whereas effective DC activation was primarily driven by the STING agonist delivered by GIP@CRTM.

To further clarify the contribution of the membrane components themselves, M and CRTM were included as additional controls. The results showed that M and CRTM exhibited comparable accumulation in homologous WEHI-164 tumor cells, further indicating that homologous tumor cell recognition was mainly mediated by the tumor cell membrane itself, and that CRT enrichment did not markedly impair the homologous targeting capability of the tumor cell membrane (Fig. S29a and b). In contrast, BMDC uptake of CRTM was significantly higher than that of M, suggesting that the CRT-mediated surface “eat-me” signal markedly promoted the uptake of membrane components by BMDCs (Fig. S29c and d). Nevertheless, treatment with M or CRTM alone induced negligible activation of the STING pathway (Fig. S29e and f). Together with the pronounced STING pathway activation induced by GIP@CRTM, these results indicate that the CRTM coating mainly contributes to dual-cell delivery to homologous tumor cells and DCs through tumor cell membrane-mediated homologous recognition and CRT-mediated pro-phagocytic signal. Robust STING pathway activation primarily depends on efficient nanocarrier-mediated delivery of the STING agonist cGAMP. In addition, flow cytometric analysis (Fig. S30) showed that CRTM treatment alone induced a certain degree of BMDC activation, which may be attributable to the membrane-associated tumor antigens carried by CRTM and enhanced CRT-mediated cellular uptake. In contrast, no significant enhancement of BMDC activation was observed after M treatment, possibly due to the lack of CRT-mediated pro-phagocytic signals, which resulted in relatively limited BMDC uptake.

Directed migration of DCs into tumors is essential for sustaining tumor-specific immune responses. Using a Transwell assay (Fig. 3g), we found that tumor cells treated with GIP@CRTM (P2) exhibited marked chemotactic attraction of immature BMDCs, with a 2.50-fold increase in migrated cells relative to the untreated group (P1). This effect may be attributed to STING pathway activation in tumor cells, which promotes chemokine secretion (e.g., CXCL10) and thereby enhances chemotactic recruitment of immature DCs. When GIP@CRTM was combined with mild phototherapy (P3), BMDC migration was further enhanced to 4.02-fold relative to the untreated group. This enhancement likely resulted from further augmentation of intratumoral STING signaling and DAMP release following combined photo-immunotherapy. In contrast, once BMDCs were fully activated by GIP@CRTM, they exhibited minimal migration toward tumor cells (P4-P6), consistent with the tendency of mature DCs to migrate to lymph nodes to initiate T cell responses rather than remain within the tumor microenvironment [54]. Based on these data, we speculate that once the nanoagonist accumulates in the tumor region, local DCs activated by the nanoagonist may migrate to the draining lymph nodes to initiate antitumor immune responses. Meanwhile, treated tumor cells may recruit surrounding immature DCs into the tumor site, thereby establishing a basis for durable antitumor immunity.

To further evaluate whether immature DCs that migrate into tumor sites can be effectively activated, we examined the indirect immunoregulatory effects of treated tumor cells on DCs in a coculture system. Flow cytometric analysis (Fig. 3h) showed that, in the absence of irradiation, tumor cells pretreated with GIP@M (G4-, 25.8%) or GIP@CRTM (G5-, 24.6%) induced moderate BMDC activation. This effect is likely attributable to type I interferon release following STING pathway activation in tumor cells. Pretreatment of tumor cells with mild phototherapy alone (G6+) increased BMDC maturation to 40.3%, consistent with tumor cell death and subsequent antigen release. Notably, tumor cells pretreated with photo-immunotherapy using GIP@M (G4+) or GIP@CRTM (G5+) further increased BMDC maturation to 63.9% and 62.8%, respectively. GIP@M and GIP@CRTM induced comparable indirect activation of BMDCs, mainly because both formulations were coated with homologous tumor cell membranes derived from WEHI-164 cells and exhibited similar intracellular accumulation in WEHI-164 tumor cells. Therefore, the immunostimulatory signals released by tumor cells after treatment with either formulation were comparable, ultimately resulting in nearly identical levels of indirect BMDC maturation. Western blot analysis further showed that BMDCs cocultured with tumor cells treated with mild phototherapy (G6+) exhibited modest STING activation, whereas coculture with tumor cells receiving GIP@CRTM-based photo-immunotherapy (G5+) produced a slight further enhancement of this effect (Fig. S31). This enhancement is likely due to the increased tumor cell death induced by GIP@CRTM-based mild photo-immunotherapy, leading to greater release of tumor-derived DNA and subsequent amplification of STING activation in BMDCs. This multilevel regulation provides a basis for subsequent T cell responses.

### 2.5 In vivo imaging and photothermal performance of the nanoagonist

**Fig. 4:**
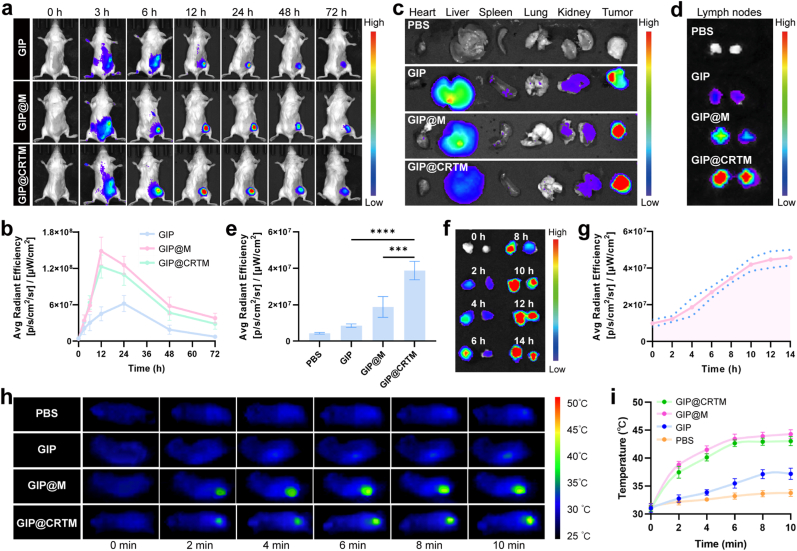
**In vivo biodistribution imaging and photothermal performance of GIP@CRTM.** (a) Fluorescence images of tumor-bearing mice at different time points after intravenous injection of various nanoagonist formulations. (b) Quantification of tumor fluorescence signals (average radiant efficiency). (c) Ex vivo fluorescence images of major organs and tumors 12 h post-injection. (d) Representative ex vivo fluorescence images of lymph nodes 12 h post-injection and (e) corresponding quantification. (f) Ex vivo fluorescence images of lymph nodes at different time points after GIP@CRTM injection and (g) corresponding quantification. (h) Photothermal images and (i) temperature-time curves of tumors under 808 nm NIR irradiation (0.5 W/cm^2^, 10 min) at 12 h after nanoagonist injection. Values were expressed as mean ± SD (n = 5). ***p < 0.001; ****p < 0.0001.

To identify the optimal time window for phototherapy, the biodistribution of the nanoagonists in tumor-bearing mice was monitored using fluorescence imaging (Fig. 4a and b). Because ICG was co-encapsulated within the nanoagonist, ICG fluorescence was used as a tracer signal for nanoagonist distribution. Following intravenous injection, GIP@M and GIP@CRTM accumulated more efficiently in tumors than GIP, with fluorescence signals peaking at 12 h post-injection, indicating that tumor enrichment of the nanoagonists was most pronounced at this time point. Ex vivo imaging confirmed higher tumor enrichment and lower off-target organ accumulation in the membrane-coated groups (Fig. 4c, Fig. S32). This biodistribution likely resulted from the homologous affinity conferred by the M and CRTM coatings. Based on these results, NIR irradiation was performed at 12 h post-injection in the subsequent therapeutic experiments, a timing expected to improve phototherapeutic efficiency at the tumor site while minimizing potential photothermal damage associated with nanoagonist accumulation in non-target tissues.

Given that GIP@CRTM markedly enhanced BMDC uptake of the nanoagonist and promoted BMDC maturation in vitro, and considering the central role of DCs in linking local tumor immune activation to T cell priming in tumor-draining lymph nodes, we further examined the nanoagonist-associated ICG fluorescence in tumor-draining inguinal lymph nodes. At 12 h post-injection, the GIP@CRTM group exhibited the strongest nanoagonist-associated ICG signals in bilateral inguinal lymph nodes, with intensities 2.07 and 4.58 times those in the GIP@M and GIP groups (Fig. 4d and e), indicating greater accumulation of GIP@CRTM in tumor-draining lymph nodes. This enhanced lymph node ICG signal may be associated with the enhanced DC recognition, uptake, and activation induced by GIP@CRTM, together with subsequent DC-related lymph-node trafficking. This inference is consistent with the stronger BMDC uptake and maturation induced by GIP@CRTM in vitro. To further determine whether this fluorescence signal was associated with lymph-node DCs, we performed flow cytometric analysis of mature DCs in the draining lymph nodes (Fig. S33). The results showed that, compared with PBS treatment, GIP@CRTM treatment significantly increased the proportion of ICG-positive cells among mature DCs in draining lymph nodes, indicating that more mature DCs carrying nanoagonist-associated ICG signals were present in the draining lymph nodes after GIP@CRTM treatment. Moreover, lymph node fluorescence gradually increased after GIP@CRTM injection and reached a plateau at approximately 10 h (Fig. 4f and g), suggesting that most tumor-infiltrating DCs activated by GIP@CRTM may have trafficked to the draining lymph nodes by this time.

Precise control of tumor temperature elevation is critical for achieving mild phototherapy while avoiding nonspecific thermal injury. Therefore, an 808-nm NIR laser with a relatively moderate power density of 0.5 W/cm^2^ was used in vivo. The ICG-equivalent dose was optimized using IP@CRTM, which shares the same ICG-loaded CRTM-coated nanocarrier structure as GIP@CRTM but lacks cGAMP, allowing the photothermal behavior of the carrier system to be assessed independently of STING agonist delivery (Fig. S34). At an ICG-equivalent dose of approximately 60 μg/mouse, the tumor temperatures in the GIP@M and GIP@CRTM groups rapidly increased to 42-45 °C under NIR irradiation and remained within the mild photothermal window during irradiation. In contrast, owing to insufficient tumor accumulation, inadequate heat generation was observed in the GIP group, and the tumor temperature failed to reach 42 °C (Fig. 4h and i). Considering inter-animal variability, tumor temperature was monitored in real time using an infrared thermal imaging system throughout irradiation, thereby avoiding unintended overheating and minimizing nonspecific thermal damage to surrounding normal tissues.

### 2.6 In vivo therapeutic efficacy and neoadjuvant performance

**Fig. 5:**
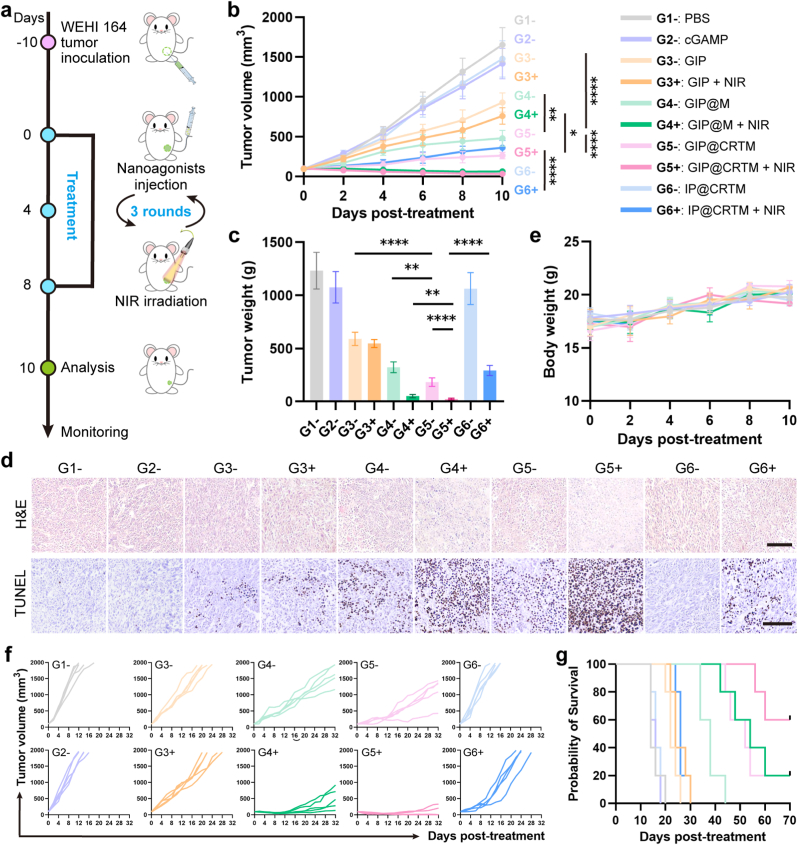
**In vivo therapeutic performance of STING nanoagonists combined with mild phototherapy.** G1: PBS, G2: cGAMP, G3: GIP, G4: GIP@M, G5: GIP@CRTM, G6: IP@CRTM. (+): tumors with NIR irradiation (808 nm, 0.5 W/cm^2^, 10 min), (−): tumors without NIR irradiation. (a) Schematic of the therapeutic strategy. (b) Tumor volume during treatment and (c) tumor weight on day 10 in fibrosarcoma-bearing mice under different regimens. (d) H&E and TUNEL staining of tumor sections after the indicated treatments. Scale bar = 100 μm. (e) Body weight of tumor-bearing mice during treatment. (f) Tumor growth curves of individual mice in each group. (g) Survival curves of fibrosarcoma-bearing mice under different treatments. Results were given as mean ± SD (n = 5). *p < 0.05; **p < 0.01; ****p < 0.0001.

We next evaluated the therapeutic efficacy of STING nanoagonists in vivo using a BALB/c subcutaneous fibrosarcoma model. A schematic of the experimental design is shown in Fig. 5a. When tumors reached approximately 100 mm^3^, this time point was designated as day 0, and mice received tail vein injections on days 0, 4, and 8. For the phototherapy combination groups, NIR irradiation was applied 12 h after each injection. Free cGAMP (G2-) showed little effect on tumor growth, likely due to its poor stability and inefficient delivery. Even without irradiation, GIP@CRTM- (G5-) exhibited markedly stronger antitumor efficacy than GIP- (G3-) and GIP@M − (G4-). When combined with mild phototherapy, both GIP@M+ (G4+) and GIP@CRTM+ (G5+) induced marked tumor regression, with GIP@CRTM+ (G5+) achieving the greatest efficacy. Importantly, GIP@CRTM+ (G5+) exhibited superior antitumor efficacy compared with mild phototherapy alone (IP@CRTM+, G6+) or immunotherapy alone (GIP@CRTM-, G5-), indicating potential synergy between mild phototherapy and STING nanoagonist treatment (Fig. 5b, c, and Fig. S35). Histopathological analysis further supported these findings. H&E and TUNEL staining showed that GIP@CRTM+ (G5+) induced the most extensive tumor necrosis and apoptosis among all groups (Fig. 5d). During treatment, no significant body weight loss was observed (Fig. 5e), no pathological changes were detected in major organs (Fig. S36), and hematological and serum biochemical parameters remained within normal ranges (Fig. S37).

Regarding long-term antitumor efficacy (Fig. 5f and g), tumors in the PBS- (G1-) and cGAMP- (G2-) groups rapidly reached the endpoint tumor volume (2000 mm^3^) within 20 days after treatment initiation. GIP@CRTM- (G5-) provided greater survival benefit than GIP- (G3-) and GIP@M − (G4-), with 20% of mice surviving until day 70. Mild phototherapy (G6+) modestly prolonged survival but failed to achieve durable tumor control. Remarkably, although GIP@CRTM+ (G5+) showed only a slight advantage over GIP@M+ (G4+) during the first 10 days, this advantage progressively increased over time. In the GIP@M+ (G4+) group, tumors resumed growth at later stages, with only 20% of mice surviving until day 70. By comparison, the GIP@CRTM+ (G5+) group achieved a 60% survival rate on day 70, and one mouse even showed complete tumor remission. Overall, both GIP@M+ (G4+) and GIP@CRTM+ (G5+) induced early tumor regression, with tumor volumes decreasing below the pretreatment baseline value of 100 mm^3^ by day 10. During subsequent follow-up, GIP@CRTM+ (G5+) achieved more durable tumor control than GIP@M+ (G4+), indicating superior long-term therapeutic benefit.

**Fig. 6:**
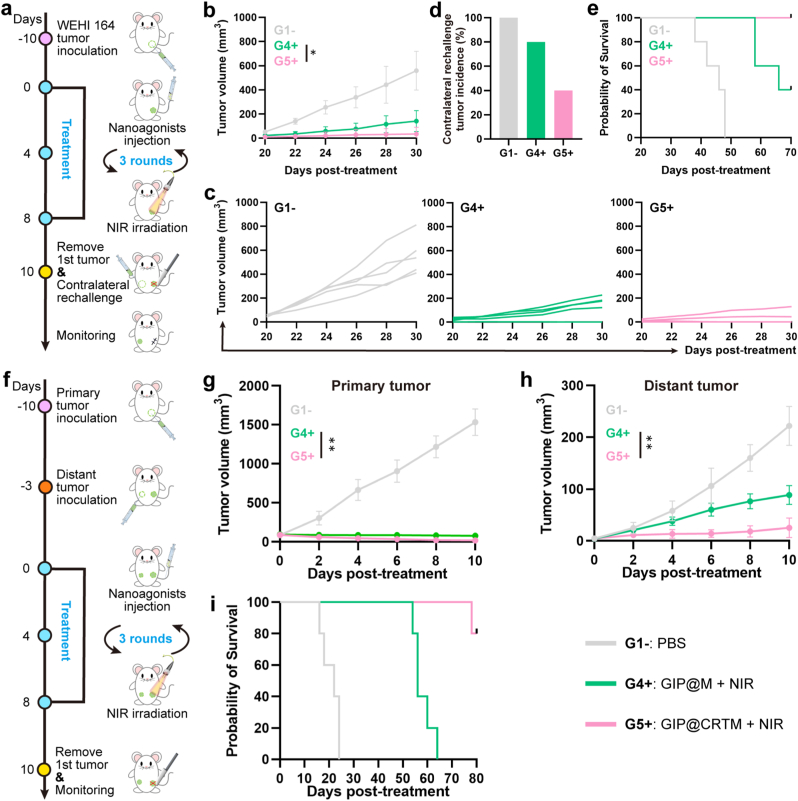
**Neoadjuvant mild photo-immunotherapy for the inhibition of postoperative contralateral rechallenge tumor outgrowth and distant tumor progression.** G1: PBS, G4: GIP@M, G5: GIP@CRTM. (+): tumors with NIR irradiation (808 nm, 0.5 W/cm^2^, 10 min). (a) Schematic overview of the postoperative contralateral tumor rechallenge model following neoadjuvant treatment with STING nanoagonists. (b) Growth curves of postoperative contralateral rechallenge tumors and (c) individual growth profiles of postoperative contralateral rechallenge tumors after different interventions. (d) Postoperative contralateral rechallenge tumor incidence in each group on day 70. (e) Survival curves of mice in different groups. (f) Schematic of the bilateral tumor model for photo-immunotherapy. (g) Growth of primary tumors and (h) distant tumors in each treatment group. (i) Survival curves of mice under different treatments. Data were presented as mean ± SD (n = 5). *p < 0.05; **p < 0.01.

In the neoadjuvant setting, effective control of postoperative tumor relapse is pivotal to improving long-term surgical outcomes but remains a major unmet clinical challenge [55,56]. To assess the perioperative applicability of neoadjuvant mild photo-immunotherapy, we established a postoperative contralateral tumor rechallenge model to determine whether this neoadjuvant strategy could suppress postoperative contralateral rechallenge tumor outgrowth (Fig. 6a). Compared with PBS-treated mice (G1-), both GIP@M+ (G4+) and GIP@CRTM+ (G5+) effectively delayed the outgrowth of postoperative contralateral rechallenge tumors, with GIP@CRTM+ (G5+) exhibiting the most pronounced inhibitory effect (Fig. 6b, c and Fig. S38). For mice that did not develop measurable postoperative contralateral rechallenge tumors, tumor volume was recorded as 0 mm^3^, resulting in overlap between the corresponding tumor growth curves and the x-axis. Remarkably, by day 70, postoperative contralateral rechallenge tumors had developed in 80% of mice in the GIP@M+ (G4+) group, with a survival rate of 40%, whereas only 40% of mice in the GIP@CRTM+ (G5+) group developed such tumors, with this group achieving 100% survival (Fig. 6d and e). Collectively, these results indicate that GIP@CRTM-based mild photo-immunotherapy, when applied as a neoadjuvant strategy, effectively suppressed the outgrowth of postoperative contralateral rechallenge tumors and prolonged survival.

Managing distant micrometastases in fibrosarcoma is particularly challenging. These lesions are small, latent, and difficult to detect by conventional imaging, yet often serve as the source of postoperative recurrence. To simulate the clinical setting of distant lesions, mice were inoculated with bilateral subcutaneous tumors, and only the larger right-sided ‘primary’ lesion received NIR irradiation. This model was used to evaluate whether mild photo-immunotherapy could effectively trigger systemic immunity to suppress the distant site (Fig. 6f). Treatment results showed that GIP@CRTM+ (G5+) led to marked suppression of both the primary and distant tumors, outperforming the GIP@M+ group (G4+) in systemic tumor control (Fig. 6g and h). On day 10 of treatment, the right-sided primary tumors were surgically resected, after which long-term prognosis was monitored. Survival analysis revealed that by day 80, all mice in the PBS-treated (G1-) and GIP@M+ (G4+) groups had reached the ethical endpoint. Notably, only one mouse in the GIP@CRTM + group (G5+) reached this endpoint, whereas the remaining mice exhibited sustained tumor suppression (Fig. 6i). Collectively, these results suggest that GIP@CRTM-based neoadjuvant mild photo-immunotherapy may provide effective control of micrometastatic lesions, thereby improving long-term prognosis and overall therapeutic benefit.

### 2.7 Mechanistic evaluation of local and systemic immune responses

**Fig. 7:**
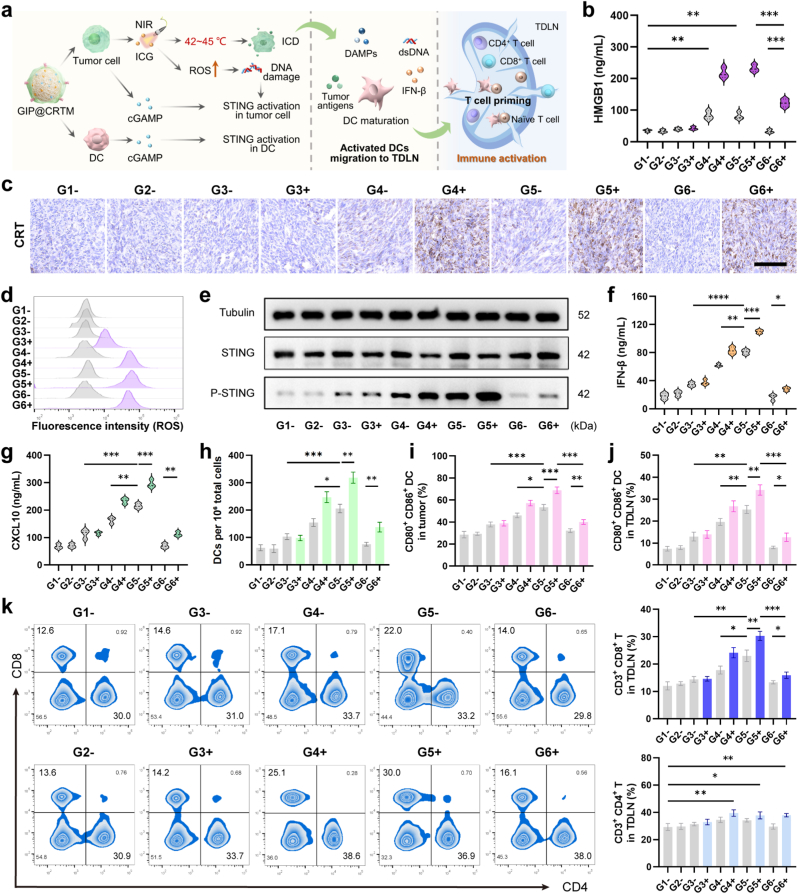
**Local tumor immune activation and lymph node T-cell priming.** G1: PBS, G2: cGAMP, G3: GIP, G4: GIP@M, G5: GIP@CRTM, G6: IP@CRTM. (+): tumors with NIR irradiation (808 nm, 0.5 W/cm^2^, 10 min), (−): tumors without NIR irradiation. (a) Schematic of the therapeutic mechanism. (b) ELISA analysis of HMGB1 levels in tumor interstitial fluid. (c) Immunohistochemical staining of CRT in tumor tissues. Scale bar = 100 μm. (d) Flow cytometric analysis of intracellular ROS levels in single-cell suspensions from tumor tissues. (e) Western blot analysis of STING and phosphorylated STING in tumor tissues. (f, g) ELISA analysis of IFN-β and CXCL10 levels in tumor tissues. (h) Quantification of tumor infiltrating DCs per 10^6^ total tumor cells under different treatments. (i, j) Flow cytometric analysis of mature DCs (CD11c^+^CD80^+^CD86^+^) in tumor tissues and tumor-draining lymph nodes (TDLNs) under different treatments. (k) Flow cytometry plots and quantification of CD8^+^ (CD3^+^CD8^+^) and CD4^+^ T cells (CD3^+^CD4^+^) in TDLNs. All data were presented as mean ± SD (n = 5). *p < 0.05; **p < 0.01; ***p < 0.001; ****p < 0.0001.

Based on prior evidence, we hypothesized that GIP@CRTM-based mild photo-immunotherapy enhances antitumor efficacy through two complementary pathways (Fig. 7a). The first pathway is driven by STING activation. GIP@CRTM activates STING signaling in both intratumoral DCs and tumor cells, thereby initiating innate immune responses within the tumor microenvironment and promoting downstream immune activation. The second pathway is mediated by mild phototherapy. ICG-mediated mild phototherapy induces ICD in tumor cells and elevates intracellular ROS levels, which further amplifies STING signaling, generating a positive immune amplification effect.

To confirm ICD induction, high mobility group box 1 (HMGB1) release (Fig. 7b, Fig. S39) and CRT exposure (Fig. 7c, Fig. S40) were evaluated in tumor tissues from different treatment groups. Only limited changes in HMGB1 release and CRT exposure were observed in the GIP@M − (G4-) and GIP@CRTM- (G5-) groups, indicating that relatively modest cellular stress responses were elicited by the STING nanoagonist alone. In contrast, mild phototherapy (G6+) significantly enhanced HMGB1 release and CRT exposure, underscoring its critical role in ICD induction. These ICD-associated signals were further amplified by the combination treatments (GIP@M+, G4+; GIP@CRTM+, G5+). This enhancement may be attributable to the cooperative effects of STING agonist delivery and ICG-mediated mild phototherapy.

To elucidate the role of phototherapy-induced ROS in STING sensitization, intracellular ROS levels in tumor tissues were further evaluated. Compared with the GIP + group (G3+), the GIP@M+ (G4+), GIP@CRTM+ (G5+), and IP@CRTM+ (G6+) groups exhibited substantially higher ROS levels in tumor tissues (Fig. 7d, Fig. S41), which were positively correlated with nanoagonist accumulation in the tumor microenvironment. STING activation was subsequently assessed by analyzing P-STING levels in tumor tissues. Western blot analysis (Fig. 7e, Fig. S42) revealed higher P-STING levels in the GIP@CRTM-group (G5-) than in the GIP@M − (G4-) and GIP- (G3-) groups, likely reflecting concurrent STING engagement in tumor cells and DCs. Mild phototherapy alone (G6+) induced modest P-STING expression. The GIP@CRTM + group (G5+) further elevated P-STING levels relative to GIP@CRTM- (G5-). Consistently, IFN-β expression, a key functional readout of STING signaling, showed the same trend as STING activation across treatment groups (Fig. 7f). In addition, enhanced STING activation led to increased CXCL10 secretion (Fig. 7g), which promoted DC infiltration into tumors (Fig. 7h).

STING-driven IFN-β production promotes DC activation within the tumor microenvironment. Flow cytometric analysis demonstrated that the proportion of mature DCs was significantly higher in the GIP@CRTM-group (G5-, 53.4%) than in the GIP@M − (G4-, 46.0%) and GIP- (G3-, 37.8%), confirming the superior capacity of GIP@CRTM to activate intratumoral DCs. When GIP@CRTM was combined with irradiation (G5+), robust ICD led to the release of abundant tumor antigens, further increasing the proportion of mature DCs to 68.9% (Fig. 7i, Fig. S43). Activated DCs within tumors subsequently migrated to tumor-draining lymph nodes. The relative changes in mature DC proportions across treatment groups in lymph nodes showed a trend similar to that observed in tumor tissues (Fig. 7j, Fig. S44). Within lymph nodes, mature DCs promoted the differentiation of naïve T cells into CD8^+^ and CD4^+^ T cell subsets (Fig. 7k). In the absence of irradiation, the proportion of CD8^+^ T cells increased from 12.1% in the PBS- group (G1-) to 17.8% in the GIP@M − group (G4-, 1.47-fold) and 23.0% in the GIP@CRTM-group (G5-, 1.90-fold). In contrast, CD4^+^ T cells exhibited only modest increases, rising from 29.3% in the PBS- group (G1-) to 34.5% in the GIP@M − group (G4-) and 34.2% in the GIP@CRTM-group (G5-). With irradiation, GIP@CRTM+ (G5+) achieved the most pronounced immune priming, elevating CD8^+^ and CD4^+^ T cell proportions to 30.3% and 37.8%, respectively. These results indicate that GIP@CRTM-based mild photo-immunotherapy not only strengthens local immune activation but also promotes T cell responses in draining lymph nodes.

**Fig. 8:**
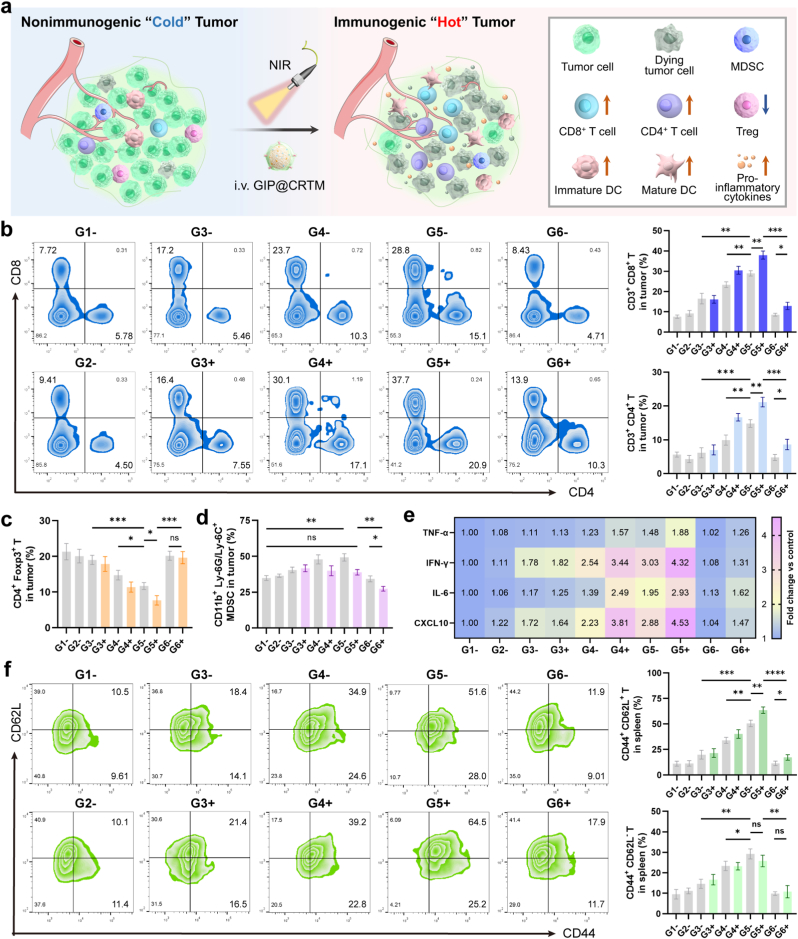
**Intratumoral immune modulation and systemic immune activation.** G1: PBS, G2: cGAMP, G3: GIP, G4: GIP@M, G5: GIP@CRTM, G6: IP@CRTM. (+): tumors with NIR irradiation (808 nm, 0.5 W/cm^2^, 10 min), (−): tumors without NIR irradiation. (a) Schematic illustration of the conversion of a non-immunogenic “cold” tumor microenvironment into an immunogenic “hot” tumor microenvironment induced by GIP@CRTM plus NIR irradiation. (b) Flow cytometric analysis of intratumoral CD8^+^ and CD4^+^ T cells after gating on CD3^+^ T cells. (c, d) Proportions of immunosuppressive cells, including Tregs (CD45^+^CD3^+^CD4^+^Foxp3^+^) (c) and MDSCs (CD45^+^CD11b^+^Ly-6G/Ly-6C^+^) (d) in tumor tissues from different groups. (e) Serum levels of TNF-α, IFN-γ, IL-6, and CXCL10 in mice after various treatments. (f) Flow cytometric analysis of Tcm (CD3^+^CD8^+^CD44^+^CD62L^+^) and Tem (CD3^+^CD8^+^CD44^+^CD62L^−^) in spleens. Values were expressed as mean ± SD (n = 5). ns, *p* > 0.05; *p < 0.05; **p < 0.01; ***p < 0.001; ****p < 0.0001.

To assess whether lymph node immune activation was reflected at the tumor site, we analyzed changes in the intratumoral immune microenvironment, as schematically illustrated in Fig. 8a. T cells that had expanded and differentiated in peripheral immune organs subsequently trafficked to tumor sites. Flow cytometric analysis (Fig. 8b) showed that, in the absence of irradiation, STING nanoagonists enhanced intratumoral CD8^+^ T cell proportions to 16.5% in the GIP- group (G3-), 23.4% in the GIP@M − group (G4-), and 29.0% in the GIP@CRTM-group (G5-), corresponding to 2.17-fold, 3.08-fold, and 3.82-fold increases relative to the PBS- group (G1-, 7.59%). The GIP@CRTM-group (G5-, 14.8%) also induced the strongest CD4^+^ T cell infiltration, representing 2.40-fold and 1.49-fold increases compared with the GIP- group (G3-, 6.16%) and the GIP@M − group (G4-, 9.91%), respectively. Combination therapy with GIP@CRTM and mild phototherapy (G5+) further amplified T cell infiltration, raising CD8^+^ and CD4^+^ T cell proportions to 38.0% and 21.2%, respectively. In contrast, IP@CRTM+ (G6+) produced only modest increases in CD8^+^ T cells (12.9%) and CD4^+^ T cells (8.6%), indicating limited modulation of the tumor immune milieu. Moreover, immunohistochemical analysis (Fig. S45) confirmed that GIP@CRTM+ (G5+) promoted greater infiltration of both CD8^+^ and CD4^+^ T cells within tumors, further corroborating its ability to promote a more immunologically active tumor microenvironment.

Infiltration by immunosuppressive cells is a key driver of tumor progression and immune evasion. STING pathway activation may contribute to limiting Treg accumulation within the tumor microenvironment [57]. In the GIP@CRTM-group (G5-), the proportion of intratumoral regulatory T cells (Tregs) decreased to 11.7%, representing a 9.6% reduction compared with the PBS- control group (G1-, 21.3%), whereas GIP@CRTM combined with NIR irradiation (G5+) further reduced Tregs to 7.67%. Mild phototherapy (G6+, 19.6%) was insufficient to markedly suppress Treg levels, potentially due to limited STING activation (Fig. 8c, Fig. S46a). Interestingly, the GIP@CRTM-group (G5-, 49.3%) increased the intratumoral proportion of myeloid-derived suppressor cells (MDSCs) compared with the PBS- group (G1-, 34.9%), whereas mild phototherapy (G6+, 27.3%) reduced MDSC infiltration. In the GIP@CRTM-based photo-immunotherapy group (G5+, 38.9%), this GIP@CRTM-associated increase in intratumoral MDSCs was partially attenuated by the addition of mild phototherapy (Fig. 8d, Fig. S46b).

To further investigate the potential mechanism by which mild phototherapy alleviated GIP@CRTM-induced MDSC accumulation, the levels of GM-CSF, CCL2, and IL-6 in tumor lysates were examined (Fig. S47). GM-CSF and CCL2 were used to assess signals associated with MDSC expansion and recruitment, respectively, whereas IL-6 was selected as a representative cytokine involved in MDSC activation and acute inflammatory responses. The results showed that GIP@CRTM treatment alone significantly increased GM-CSF and CCL2 levels, suggesting the induction of a myeloid feedback response conducive to MDSC expansion and recruitment. In contrast, IP@CRTM-mediated mild phototherapy did not markedly alter GM-CSF levels, but it significantly reduced CCL2 levels, indicating that phototherapy alone may reduce MDSC accumulation in the tumor region mainly by weakening CCL2-associated MDSC recruitment signals. Importantly, the addition of mild phototherapy attenuated the GIP@CRTM-induced upregulation of GM-CSF and CCL2, whereas IL-6 remained at a relatively high level. These results indicate that mild phototherapy-mediated MDSC reduction was not mediated by global suppression of inflammation. Instead, mild phototherapy may partially alleviate GIP@CRTM-associated MDSC accumulation by reshaping the GIP@CRTM-induced inflammatory cytokine microenvironment and weakening GM-CSF/CCL2-related myeloid expansion and recruitment signals.

To further assess systemic immune activation, serum levels of TNF-α, IFN-γ, IL-6, and CXCL10 were quantified (Fig. 8e, Fig. S48). Compared with the other groups, the GIP@CRTM + group (G5+) induced significantly elevated concentrations of these cytokines without observable clinical abnormalities, indicating effective systemic immune activation. We next assessed splenic memory T cell subsets to evaluate immune memory induction (Fig. 8f). Flow cytometric analysis demonstrated that STING nanoagonist treatment promoted the formation of both central memory T cells (Tcm) and effector memory T cells (Tem). Compared with the GIP- group (G3-, Tcm: 19.8%, Tem: 14.6%) and the GIP@M − group (G4-, Tcm: 34.0%, Tem: 23.4%), the GIP@CRTM-group induced higher proportions of memory T cells (G5-, Tcm: 50.6%, Tem: 29.3%). When combined with mild phototherapy, the GIP@CRTM + group (G5+) further increased the proportion of Tcm to 63.6%, markedly surpassing all other groups. Together, these results demonstrate that GIP@CRTM-based mild photo-immunotherapy elicits robust systemic immune activation and supports the establishment of durable antitumor immune memory.

Based on the above results, the key differences between GIP@CRTM and GIP@M are primarily reflected in immune regulation. Because both formulations are coated with homologous tumor cell membranes derived from WEHI-164 cells, they possess comparable homologous tumor-recognition ability and therefore exhibit similar performance in tumor cell uptake, mild photothermal heating, and several tumor cell-related therapeutic indicators. Notably, the key advantage of GIP@CRTM lies in the “eat-me” signal conferred by CRT, which enhances DC uptake of the nanoagonist, promotes DC maturation, and strengthens STING activation in DCs. This CRT-mediated immune-amplifying effect is more evident in immune-dependent therapeutic outcomes, including long-term tumor control, suppression of postoperative contralateral rechallenge tumor outgrowth, inhibition of distant tumors, and survival benefits. Therefore, in the present study, the key advantage of GIP@CRTM-mediated mild photo-immunotherapy over the corresponding GIP@M-mediated mild photo-immunotherapy lies not primarily in enhanced short-term local tumor killing, but rather in its greater capacity to promote DC-mediated immune priming and establish a more durable systemic antitumor immune response, ultimately resulting in more pronounced long-term therapeutic benefits.

## 3 Conclusions

Given the limited neoadjuvant options for fibrosarcoma and the need to improve perioperative disease control, we developed a biomimetic dual-driven STING nanoagonist for neoadjuvant mild photo-immunotherapy. The nanoagonist, cGAMP/ICG@PLGA@CRTM (GIP@CRTM), co-encapsulated a STING agonist (cGAMP) and a photosensitizer (ICG) within a PLGA core and was cloaked with CRT-enriched fibrosarcoma cell membranes (CRTM). This membrane camouflage promoted preferential homologous tumor accumulation, while surface-exposed CRT enhanced uptake by DCs. Consequently, GIP@CRTM achieved dual-driven STING activation in both tumor cells and DCs, thereby strengthening innate immune priming. Upon NIR irradiation, ICG enabled mild phototherapy by inducing mild hyperthermia and ROS generation. Mild heating promoted ICD to enhance tumor immunogenicity, while ROS-driven DNA damage and cytosolic DNA leakage further amplified tumor-intrinsic STING signaling. This coordinated cascade remodeled the tumor immune microenvironment by increasing intratumoral DC recruitment and maturation, promoting infiltration of CD8^+^ cytotoxic T cells and CD4^+^ helper T cells, and reducing immunosuppressive Tregs. Notably, mild phototherapy attenuated the increase in intratumoral MDSC abundance observed after STING nanoagonist treatment alone, suggesting that the incorporation of phototherapy may further contribute to GIP@CRTM-mediated remodeling of the tumor immune microenvironment. In parallel, the expansion of splenic memory T cell subsets supported durable immune memory and sustained immune surveillance. Functionally, when implemented as a neoadjuvant intervention, GIP@CRTM-mediated mild photo-immunotherapy achieved effective preoperative tumor reduction, suppressed the outgrowth of postoperative contralateral rechallenge tumors, inhibited the progression of distant small lesion, and prolonged survival in murine fibrosarcoma models.

Taken together, the above therapeutic mechanisms and antitumor efficacy indicate that the proposed strategy offers multiple integrated advantages over conventional STING immunotherapy alone or phototherapy alone. Compared with existing STING-based immunotherapeutic strategies, this strategy is not merely aimed at improving the delivery efficiency of STING agonists. Instead, a biomimetic regulatory interface was constructed using CRT-enriched homologous tumor cell membranes, thereby endowing the nanoplatform with both homologous tumor-targeting capability and enhanced uptake by DCs. Accordingly, dual-cell STING activation in both tumor cells and DCs was achieved. When further combined with mild phototherapy, this combination strategy could compensate for the limitations of STING immunotherapy alone in rapid preoperative tumor debulking and antigen release. In addition, the incorporation of mild phototherapy partially reduced the MDSC upregulation associated with GIP@CRTM treatment alone, thereby further improving the immune microenvironment in the tumor region. Compared with phototherapy alone, this strategy is also not confined to local photodamage and ICD induction. Rather, ROS generated by ICG-mediated phototherapy were utilized to induce DNA damage, thereby further amplifying endogenous STING signaling in tumor cells. Meanwhile, exogenous cGAMP-mediated STING activation further facilitated the conversion of phototherapy-induced local immunogenic signals into systemic antitumor immunity, thereby addressing the limitations of phototherapy alone in distant lesion control, postoperative recurrence suppression, and immune memory establishment. Therefore, this study provides a mechanistically complementary STING-phototherapy synergistic therapeutic strategy for the neoadjuvant treatment of fibrosarcoma and demonstrates systemic therapeutic advantages in preoperative tumor reduction, postoperative contralateral rechallenge tumor suppression, and distant lesion control.

Although GIP@CRTM-mediated mild photo-immunotherapy achieved encouraging therapeutic efficacy in murine fibrosarcoma models, the observation that mild phototherapy attenuated intratumoral MDSC accumulation warrants further investigation. Future clarification of MDSC functional changes during this therapeutic process and their potential contribution to antitumor immune responses will help provide a more comprehensive understanding of the immunological mechanisms underlying GIP@CRTM-mediated mild photo-immunotherapy. In addition, further validation in more clinically relevant models, together with systematic optimization of dosing, treatment regimens, and irradiation parameters, will help refine this therapeutic strategy and facilitate its clinical translation. Overall, this work defines a neoadjuvant mild photo-immunotherapeutic framework for fibrosarcoma by combining biomimetic nanotechnology with STING-mediated immunomodulation and mild phototherapy. By integrating localized preoperative tumor reduction with systemic immune reinforcement, this platform not only expands neoadjuvant treatment options for fibrosarcoma but may also inform the rational design of treatment strategies for other tumors in which STING signaling is amenable to therapeutic modulation.

## 4 Materials and methods

### 4.1 Materials

Polyvinyl alcohol (PVA), poly(lactic-co-glycolic acid) (PLGA, 50:50), and the STING agonist 2′,3′-cGAMP were purchased from Sigma-Aldrich. DOX and ICG were obtained from Aladdin. The green fluorescent membrane probe DiO, nuclear stain DAPI, Cell Counting Kit-8 (CCK-8), and ROS detection kit were purchased from Beyotime Biotechnology. Mouse monoclonal antibodies targeting calreticulin (CRT) and high-mobility group box 1 (HMGB1) were obtained from Cell Signaling Technology, whereas recombinant murine interleukin-4 (IL-4) and granulocyte-macrophage colony-stimulating factor (GM-CSF) were supplied by PeproTech. ELISA kits for mouse CXCL10, IL-6, IFN-β, TNF-α, and IFN-γ were purchased from BioLegend. Fluorescently conjugated antibodies for flow cytometry were also obtained from BioLegend, including CD45 (BV510, clone 30-F11), CD3 (BV605, clone 145-2C11), CD4 (APC, clone GK1.5), CD8a (PE, clone 53-6.7), CD44 (BV421, clone IM7), CD62L (PE-CY7, clone MEL-14), CD11c (APC, clone N418), CD80 (PE-CY7, clone 16-10A1), CD86 (PE, clone GL-1), CD11b (BV605, clone M1/70), Ly-6G/Ly-6C (BV421, clone RB6-8C5), and Foxp3 (BV421, clone MF-14). All chemicals and reagents were of analytical grade and used as received without further purification.

### 4.2 Cell culture and animal husbandry

Mouse fibrosarcoma cell line WEHI-164 was obtained from the American Type Culture Collection (ATCC® CRL-1751™, RRID: CVCL_2251). The cell line was authenticated and confirmed to be free of mycoplasma contamination before use. Cells were maintained in Dulbecco's modified Eagle's medium (DMEM) containing 10% fetal bovine serum (FBS) and 1% penicillin-streptomycin, and incubated at 37 °C in a humidified atmosphere of 5% CO_2_ under aseptic culture conditions.

Bone marrow-derived dendritic cells (BMDCs) were generated using a classical protocol. Briefly, femurs and tibias were harvested from euthanized BALB/c mice, and bone surfaces were disinfected with 75% ethanol. After removing both ends of the bones under a biosafety cabinet, bone marrow cells were harvested by flushing with RPMI-1640 medium, passed through a 70 μm cell strainer, and collected via centrifugation at 1500 rpm for 5 min. The resulting pellet was treated with 2 mL of red blood cell lysis buffer for 3 min at room temperature, neutralized with 10 mL PBS, and centrifuged again at 2000 rpm for 5 min. The obtained cells were then resuspended in complete RPMI-1640 medium supplemented with 10% FBS, IL-4 (10 ng/mL), and GM-CSF (20 ng/mL) and maintained at 37 °C in a 5% CO_2_ humidified atmosphere. Half of the culture medium was refreshed every other day. On day 6, immature BMDCs suspended in the medium were harvested by gently blowing the culture medium with a pipette for subsequent experiments.

BALB/c mice were obtained from GemPharmatech. All animal procedures were performed with the approval of the Animal Ethics Committee of Xiamen University [XMULAC20210114] and in full compliance with institutional and national guidelines for the care and use of laboratory animals.

### 4.3 In vitro pretreatment of WEHI-164 cells

To induce elevated surface expression of CRT, WEHI-164 cells were subjected to DOX pretreatment. Specifically, cells were seeded in 6-well plates and exposed to 2.5 μM DOX for 24 h after cell adherence. Following treatment, cell viability was measured using a CCK-8 assay, and CRT expression was evaluated by flow cytometry (Beckman Cytoflex LX) and immunofluorescence confocal microscopy (Zeiss LSM 880). CRT-upregulated WEHI-164 cells were harvested and cryopreserved in liquid nitrogen for subsequent experiments.

### 4.4 Cell membrane extraction

DOX-pretreated WEHI-164 cells were resuspended in PBS and lysed on ice using a sonicator (Diagenode Bioruptor Plus, 20 W, 2 s on/2 s off, total 5 min). The lysates were centrifuged at 5000 g for 20 min at 4 °C, and the supernatant was subsequently centrifuged at 20000 g for 1 h to isolate tumor cell membranes (CRTM), which were stored at −80 °C until use. As a control, untreated WEHI-164 cells underwent the same extraction procedure to obtain native tumor cell membranes (M), which were employed for comparative evaluation of biomimetic membrane-coating functional differences.

### 4.5 Construction of the biomimetic nanoagonist

GIP (cGAMP/ICG@PLGA) was fabricated using a double emulsion-solvent evaporation method. Briefly, 200 μL of DMSO solution containing 10 mg cGAMP and 50 mg ICG was added to 1 mL dichloromethane containing 100 mg PLGA and thoroughly mixed. The resulting mixture was emulsified in 3 mL of 7% PVA solution under ice-bath conditions and sonicated using a probe sonicator (100 W, 30 s) to generate a primary emulsion. This emulsion was immediately introduced into 50 mL of 1% PVA solution and further sonicated for 60 s to form a stable double emulsion. The emulsion was stirred at room temperature to evaporate the organic solvent, and GIP was subsequently collected by centrifugation.

The initial feeding ratio of cGAMP and ICG was determined based on the requirements for mild phototherapy and the preliminary in vivo therapeutic efficacy. First, under fixed 808 nm laser irradiation conditions, IP@CRTM was used to optimize the ICG-equivalent dose required to achieve mild temperature elevation at the tumor site. IP@CRTM shares the same ICG-loaded CRTM-coated nanocarrier structure as GIP@CRTM but lacks cGAMP, thereby allowing the photothermal behavior of the carrier system to be evaluated independently of STING agonist delivery. The results showed that an ICG-equivalent dose of approximately 60 μg per mouse was sufficient to increase the tumor temperature into the mild phototherapy range (Fig. S34). Based on this ICG dose, the in vivo antitumor efficacy of different cGAMP feeding ratios was further evaluated. It was found that an ICG:cGAMP ratio of 1:0.2 was sufficient to produce a marked tumor-inhibitory effect (Fig. S49). Therefore, 10 mg of cGAMP and 50 mg of ICG were ultimately selected as the initial feeding amounts for GIP preparation.

For biomimetic coating, CRTM (100 μg/mL) was mixed with GIP solution (100 μg/mL) and treated under ice-bath conditions with intermittent sonication (10 W, 2 s on/2 s off). To achieve uniform coating, the mixture was extruded ten times sequentially through 400 nm and 200 nm polycarbonate membranes using a mini-extruder (Avanti Polar Lipids). Excess unbound vesicles were removed by centrifugation, and the resulting GIP@CRTM was stored at 4 °C for subsequent use. As a control, GIP@M was prepared following the same procedure using membranes isolated from untreated WEHI-164 cells.

### 4.6 Physicochemical characterization of the nanoagonist

The hydrodynamic size and zeta potential of the nanoagonists were determined using a Zetasizer Nano ZSP (Malvern, ZEN5600). Particle morphology was characterized by transmission electron microscopy (JEOL JEM-1400). Flow cytometry was carried out on a CytoFLEX LX system (Beckman Coulter). Fluorescence imaging was performed using a confocal laser scanning microscope (Zeiss LSM 880). The cGAMP loading capacity and release kinetics of the nanoagonists were quantified by high-performance liquid chromatography (Shimadzu Prominence LC-20A) at 256 nm. Protein composition was examined by SDS-PAGE and Western blot analysis using a Bio-Rad electrophoresis apparatus. The encapsulation efficiency of ICG was evaluated by UV-Vis spectroscopy (Agilent Cary 60) at its characteristic absorbance peak of 780 nm. The encapsulation efficiency and drug loading content were calculated according to the following equations:

Encapsulationefficiency(%)=amountofencapsulateddrug/initialamountofdrug×100%.

Drugloadingcontent(%)=amountofencapsulateddrug/totaldrymassofnanoparticles×100%.

### 4.7 In vitro cGAMP release study

The release of cGAMP from GIP and GIP@CRTM was evaluated by suspending equal amounts of cGAMP-loaded formulations in 2 mL PBS and enclosing them in dialysis bags with a molecular weight cutoff (MWCO) of 3500 Da. The dialysis bags were immersed in 10 mL PBS (pH 7.4 or pH 5.0) to simulate physiological and lysosomal environments, respectively. All samples were incubated at 37 °C in a thermostatic shaker. At predetermined time points, 100 μL of dialysate was withdrawn for analysis, and an equal volume of fresh PBS was replenished to maintain a constant total volume. The amount of cGAMP released was quantified by Shimadzu Prominence LC-20A.

### 4.8 Hemolysis assay

The hemolytic activity of GIP@CRTM was evaluated using red blood cells (RBCs) isolated from healthy mice. RBCs were washed three times with PBS and collected by centrifugation at 500*g* for 5 min. Subsequently, 0.8 mL of GIP@CRTM suspension was mixed with 0.2 mL of RBC suspension and incubated at 37 °C for 4 h. After incubation, the mixtures were centrifuged at 1000 g for 5 min to collect the supernatants. The absorbance of the supernatants was recorded at 560 nm using a microplate reader (Thermo Fisher Varioskan Flash). Distilled water (DW) and PBS were used as the positive and negative controls, respectively. The hemolysis ratio was determined according to the following equation: Hemolysis (%) = (A_NA_ - A_PBS_)/(A_DW_ - A_PBS_) × 100%, where A_NA_, A_PBS_, and A_DW_ represent the absorbance of the nanoagonist solution, PBS, and distilled water, respectively.

### 4.9 In vitro cellular uptake

The cellular uptake efficiency of nanoagonist formulations by homologous fibrosarcoma cells and BMDCs was examined. Three formulations were tested: GIP, GIP@M, and GIP@CRTM. WEHI-164 cells were incubated with nanoagonists containing cGAMP (3 μg/mL) and ICG (18.6 μg/mL) for 6 h. For BMDCs, nanoagonists containing cGAMP (1.5 μg/mL) and ICG (9.3 μg/mL) were introduced and incubated for 4 h. After incubation, the cells were rinsed with PBS to eliminate unbound nanoagonists. They were then fixed in 4% paraformaldehyde for 20 min at room temperature and counterstained with DAPI (10 μg/mL) for nuclear visualization. Intracellular fluorescence distribution was visualized using a confocal laser scanning microscope. Quantitative nanoagonist uptake was determined by flow cytometry.

### 4.10 Photoresponsive properties in tumor cells

The photothermal effects of nanoparticles in tumor cells were investigated. WEHI-164 cells were seeded in 96-well plates and allowed to adhere. Nanoparticles containing ICG (18.6 μg/mL), including GIP, GIP@M, GIP@CRTM, and IP@CRTM, were added and incubated at 37 °C for 6 h to achieve cellular uptake. After incubation, cells were exposed to 808 nm NIR laser irradiation (0.5 W/cm^2^) for 8 min. Real-time temperature changes in each well were recorded at 2 min intervals using an infrared thermal imager (FLIR E54, FLIR Systems) to compare the photothermal performance of different formulations.

To further assess ROS generation upon irradiation, cells were incubated with 10 μM DCFH-DA solution at 37 °C for 20 min, and then irradiated (808 nm, 0.5 W/cm^2^, 8 min). Immediately after laser exposure, intracellular ROS levels were assessed. DCF fluorescence was visualized by confocal laser scanning microscope Zeiss LSM 880 (λ_ex_ = 488 nm, λ_em_ = 525 nm). Quantitative analysis of mean fluorescence intensity (MFI) was carried out by Beckman CytoFLEX LX.

### 4.11 In vitro detection of immunogenic cell death markers

WEHI-164 cells were plated in 6-well dishes, and the growth medium was substituted with preparations containing the nanoagonists co-loaded with cGAMP (3 μg/mL) and ICG (18.6 μg/mL). The cells were incubated for 6 h and then exposed to an 808 nm NIR laser at 0.5 W/cm^2^ for 8 min. CRT Translocation Assay: After treatment, cells were first incubated with an anti-CRT primary antibody (1:800) at 37 °C for 30 min, followed by staining with an Alexa Fluor 647-conjugated secondary antibody (λ_ex_ = 650 nm, λ_em_ = 668 nm). The samples were examined by flow cytometry or counterstained with DAPI and observed under a confocal laser scanning microscope to assess the distribution of CRT. HMGB1 Release Assay: The cells were fixed in 4% paraformaldehyde for 15 min and subsequently permeabilized using 0.1% Triton X-100. They were then incubated with an anti-HMGB1 primary antibody (1:100) overnight at 4 °C, followed by staining with an Alexa Fluor 488-conjugated secondary antibody (λ_ex_ = 495 nm, λ_em_ = 519 nm). After nuclear counterstaining with DAPI, HMGB1 localization was examined by confocal microscopy. ATP Release Assay: The cell culture supernatants were harvested and centrifuged at 300 g for 5 min to eliminate residual cellular debris. Extracellular ATP concentrations were determined with an ATP assay kit following the manufacturer's protocol. Bioluminescence was recorded using a multifunctional microplate reader (Thermo Fisher Varioskan Flash).

### 4.12 In vitro DC activation

To evaluate the direct stimulatory effect of nanoagonists on DCs, immature BMDCs were incubated with GIP@CRTM at concentrations equivalent to cGAMP (1.5 μg/mL) and ICG (9.3 μg/mL). After 24 h, cells were harvested and stained with anti-CD11c (APC), anti-CD80 (PE-Cy7), and anti-CD86 (PE) antibodies (1:200 dilution) at 4 °C in the dark for 30 min. Flow cytometry was performed to assess BMDC maturation. As controls, immature BMDCs were treated with PBS, free cGAMP, GIP, GIP@M, or IP@CRTM, followed by identical staining and analysis procedures to compare their effects on DC activation.

To further assess the indirect activation of BMDCs by mild photo-immunotherapy, WEHI-164 cells were treated with GIP@CRTM containing cGAMP (3 μg/mL) and ICG (18.6 μg/mL). Immature BMDCs were cocultured with pretreated tumor cells in the same wells for 12 h. At the end of coculture, suspended cells were stained with anti-CD11c (APC), anti-CD80 (PE-Cy7), and anti-CD86 (PE) antibodies (1:200 dilution) at 4 °C in the dark for 30 min. Flow cytometric analysis was conducted to evaluate the proportion of mature BMDCs.

### 4.13 Western blot analysis

Protein expression was analyzed by Western blot. The treated cells were lysed on ice for 30 min in RIPA buffer containing protease and phosphatase inhibitors. The lysates were centrifuged at 12,000 rpm for 10 min, and the resulting supernatants were collected. Total protein concentrations were determined using a BCA assay kit (Thermo Scientific). Equal amounts of protein were combined with 5× SDS loading buffer and denatured by boiling at 95 °C for 5 min. The samples were then separated on 10% SDS-PAGE gels and transferred onto PVDF membranes (Millipore). The membranes were blocked with 5% nonfat milk at room temperature for 1.5 - 2 h, or with 5% BSA when detecting phosphorylated proteins, followed by incubation with the corresponding primary antibodies overnight at 4 °C. After washing, the membranes were incubated with HRP-conjugated secondary antibodies for 1 h at room temperature. The protein bands were then visualized using an enhanced chemiluminescence detection reagent (Millipore) and imaged with a ChemiDoc MP system (Bio-Rad).

### 4.14 In vitro DC chemotaxis assay

A Transwell migration assay was employed to determine whether the GIP@CRTM-based therapeutic strategy enhances BMDC chemotaxis toward tumor cells. WEHI-164 tumor cells were seeded in the lower chambers of a 12-well Transwell system, and immature BMDCs (1 × 10^5^ cells/100 μL) were added to the upper chambers. Six experimental groups were designed: P1, untreated control (tumor cells and BMDCs untreated); P2, tumor cells treated with GIP@CRTM only; P3, tumor cells treated with GIP@CRTM followed by NIR irradiation (808 nm, 0.5 W/cm^2^, 8 min); P4, BMDCs treated with GIP@CRTM only; P5, both tumor cells and BMDCs treated with GIP@CRTM; and P6, both tumor cells and BMDCs treated with GIP@CRTM, with tumor cells additionally exposed to NIR irradiation (808 nm, 0.5 W/cm^2^, 8 min). Following treatment, the Transwell system was incubated at 37 °C with 5% CO_2_ for 24 h. Suspended cells in the lower chambers were collected and stained with anti-CD11c antibody. Migrated BMDCs in the lower chambers were evaluated by flow cytometry.

### 4.15 In vivo imaging and tumor heating

The biodistribution of GIP, GIP@M, and GIP@CRTM in tumor-bearing mice was evaluated. Nanoagonists were administered via tail vein injection at doses of 10 μg cGAMP and 62.1 μg ICG per mouse. Whole-body fluorescence imaging was conducted at 0, 3, 6, 12, 24, 48, and 72 h after injection using an IVIS Spectrum system (PerkinElmer). Fluorescence intensity within the tumor region was quantified by ROI analysis using Living Image software to determine nanoagonist accumulation. At 12 h post-injection, corresponding to the peak tumor signal of ICG, mice were euthanized and major organs (heart, liver, spleen, lungs, kidneys), tumor tissues, and adjacent inguinal lymph nodes were excised. Ex vivo fluorescence imaging was performed by placing tissues on a black background, and ROI-based quantification was conducted to delineate the organ-level distribution profile of nanoagonists. For lymph node fluorescence analysis, the left and right inguinal lymph nodes excised from each mouse were quantified separately, and the average of the bilateral signals was used as the representative lymph node fluorescence value for that mouse in the statistical analysis. To assess the photothermal performance of the nanoagonists in vivo, tumors were exposed to an 808 nm NIR laser at 0.5 W/cm^2^ for 10 min, 12 h after injection. Real-time temperature variations within the tumor area were recorded using an infrared thermal imaging system (FLIR E54), enabling comparative evaluation of the heating effects induced by different formulations.

### 4.16 In vivo antitumor therapy

A subcutaneous tumor model was generated by inoculating 6-8-week-old BALB/c mice in the right lower flank with 1 × 10^6^ WEHI-164 cells suspended in 100 μL of PBS. When tumors reached approximately 100 mm^3^, treatment was initiated and designated as day 0. The tumor-bearing mice were randomly divided into ten groups (n = 5 per group): G1-, PBS control; G2-, free cGAMP (10 μg per mouse); G3-, GIP (cGAMP 10 μg per mouse, ICG 62.1 μg per mouse); G3+, GIP + NIR (cGAMP 10 μg per mouse, ICG 62.1 μg per mouse); G4-, GIP@M (cGAMP 10 μg per mouse, ICG 62.1 μg per mouse); G4+, GIP@M + NIR (cGAMP 10 μg per mouse, ICG 62.1 μg per mouse); G5-, GIP@CRTM (cGAMP 10 μg per mouse, ICG 62.1 μg per mouse); G5+, GIP@CRTM + NIR (cGAMP 10 μg per mouse, ICG 62.1 μg per mouse); G6-, IP@CRTM (ICG 62.1 μg per mouse); and G6+, IP@CRTM + NIR (ICG 62.1 μg per mouse). Treatments were administered via tail vein injection every 4 days for a total of three injections. For phototherapy groups, tumors were irradiated with an 808 nm laser (0.5 W/cm^2^, 10 min) under anesthesia at 12 h post-injection. Tumor temperatures were continuously monitored using an infrared thermal imaging system, and those in the G4+, G5+, and G6+ groups were strictly maintained at 42-45 °C to ensure mild photothermal treatment. In contrast, tumors in the G3+ group failed to exceed 42 °C and therefore required no additional temperature control. Tumor sizes were recorded every other day, and the volume was calculated using formula V = (A × B^2^)/2, where A and B denote the tumor length and width, respectively. Mice were euthanized when tumor volumes reached 2000 mm^3^ in accordance with ethical guidelines. For Kaplan–Meier survival analyses in the tumor models, mice that died or reached a predefined humane endpoint (tumor volume = 2000 mm^3^) were counted as events, whereas mice that remained alive without reaching the endpoint by the end of the observation period were right-censored at the final follow-up time point.

Postoperative contralateral tumor rechallenge model: To evaluate whether neoadjuvant treatment could suppress postoperative contralateral rechallenge tumor outgrowth, mice in the G4+ and G5+ groups underwent surgical resection of the right-sided tumors on day 10. At the same time, WEHI-164 cells were subcutaneously inoculated into the contralateral left flank to establish the postoperative contralateral tumor rechallenge model. Growth of the postoperative contralateral rechallenge tumors was monitored every 2 days.

Bilateral Tumor Model: To further evaluate the impact of treatments on distant lesions, bilateral tumor models were generated in G4+ and G5+ groups. WEHI-164 cells were inoculated into the right and left flanks on days −10 and −3, respectively. During treatment, only right-side primary tumors were exposed to laser irradiation. Tumor growth on both sides was measured every 2 days.

### 4.17 In vivo immune microenvironment analysis

To investigate the modulation of the immune microenvironment by different treatment strategies, tumors, inguinal lymph nodes, and spleens were collected and kept in PBS at 4 °C. Tissues were minced into ∼1 mm^3^ fragments, mechanically dissociated on ice, and filtered through 70 μm strainers to remove debris. Cell suspensions were treated with RBC lysis buffer to eliminate erythrocytes, followed by PBS washing. For tumor samples, immune cells were further enriched by Percoll density gradient centrifugation. Briefly, cells were resuspended in 4 mL of 40% Percoll and overlaid onto 3 mL of 80% Percoll. The gradients were centrifuged at 800 g for 30 min with the brake disengaged, and the cells located at the interphase were harvested. This step was not required for lymph node or spleen samples. Single-cell suspensions were incubated with fluorophore-labeled antibodies (1:200 dilution) for 30 min at 4 °C in the dark. After being washed twice with PBS, the samples were analyzed using a CytoFLEX LX flow cytometer (Beckman Coulter), and the data were processed with FlowJo software. Gating strategies are provided in Supporting Information Fig. S50. To further evaluate systemic inflammatory responses, blood samples were collected and centrifuged at 3000 rpm for 10 min to obtain serum. The concentrations of key cytokines, including TNF-α, IFN-γ, IL-6, and CXCL10, were determined using commercial ELISA kits in accordance with the manufacturer's protocols.

### 4.18 In vivo toxicity evaluation

Systemic toxicity associated with different treatment regimens was evaluated. Body weights were monitored every other day throughout the treatment period to track potential adverse responses. At the completion of therapy, the mice were euthanized, and the major organs, including the heart, liver, spleen, lungs, and kidneys, were collected for histopathological analysis. The tissues were fixed in 4% paraformaldehyde for 24 h, dehydrated through a graded ethanol series, embedded in paraffin, sectioned at a thickness of 5 μm, and stained with hematoxylin and eosin (H&E). Stained sections were examined and imaged under a Leica DM 3000 optical microscope to identify potential histopathological alterations. In parallel, blood biochemical analyses were performed by a fully automated biochemical analyzer (BS-240vet, Mindray) to further assess systemic physiological effects induced by the treatments.

### 4.19 Statistical analysis

All data are presented as the mean ± standard deviation (SD), and each experiment was independently performed at least three times. Statistical differences between two groups were evaluated using an unpaired two-tailed Student's t-test. For comparisons involving more than two groups, one-way analysis of variance (ANOVA) followed by Tukey's post hoc multiple-comparisons test was performed when all pairwise comparisons were required. When multiple treatment groups were compared with a single control group, one-way ANOVA followed by Dunnett's post hoc test was used. For datasets involving two independent variables, two-way ANOVA followed by Šídák's multiple-comparisons test was applied. A *p* value < 0.05 was considered statistically significant, and significance levels were defined as follows: *p < 0.05, **p < 0.01, ***p < 0.001, ****p < 0.0001. All statistical analyses were conducted using GraphPad Prism software.

## CRediT authorship contribution statement

**Zhao Wang:** Conceptualization, Methodology, Writing – original draft. **Yuan Ma:** Investigation, Methodology. **Wangwei Zhang:** Methodology, Visualization. **Xiaoheng Dai:** Formal analysis, Methodology. **Xiao Zhou:** Validation. **Zhirong Zhang:** Validation. **Zhenhang Lin:** Validation. **Yilai Gao:** Validation. **Wei Zeng:** Project administration, Supervision. **Guohong Zhuang:** Project administration, Supervision. **Ting Wu:** Supervision, Writing – review & editing. **Wengang Li:** Supervision, Writing – review & editing.

## Declaration of competing interest

The authors declare that they have no known competing financial interests or personal relationships that could have appeared to influence the work reported in this paper.

## Data Availability

Data will be made available on request.
