# Supplementary material for: Biomimetic dual-driven STING nanoagonist orchestrates neoadjuvant mild photo-immunotherapy for fibrosarcoma
Source: Mater Today Bio. 2026 Jun 23;39:103386. doi: 10.1016/j.mtbio.2026.103386 (PMC13320259; doi:10.1016/j.mtbio.2026.103386)
Supplement: Multimedia component 1 [file mmc1.docx]

**Supporting Information**

**Biomimetic** **dual-driven STING nanoagonist orchestrates neoadjuvant mild** **photo-immunotherapy for fibrosarcoma**


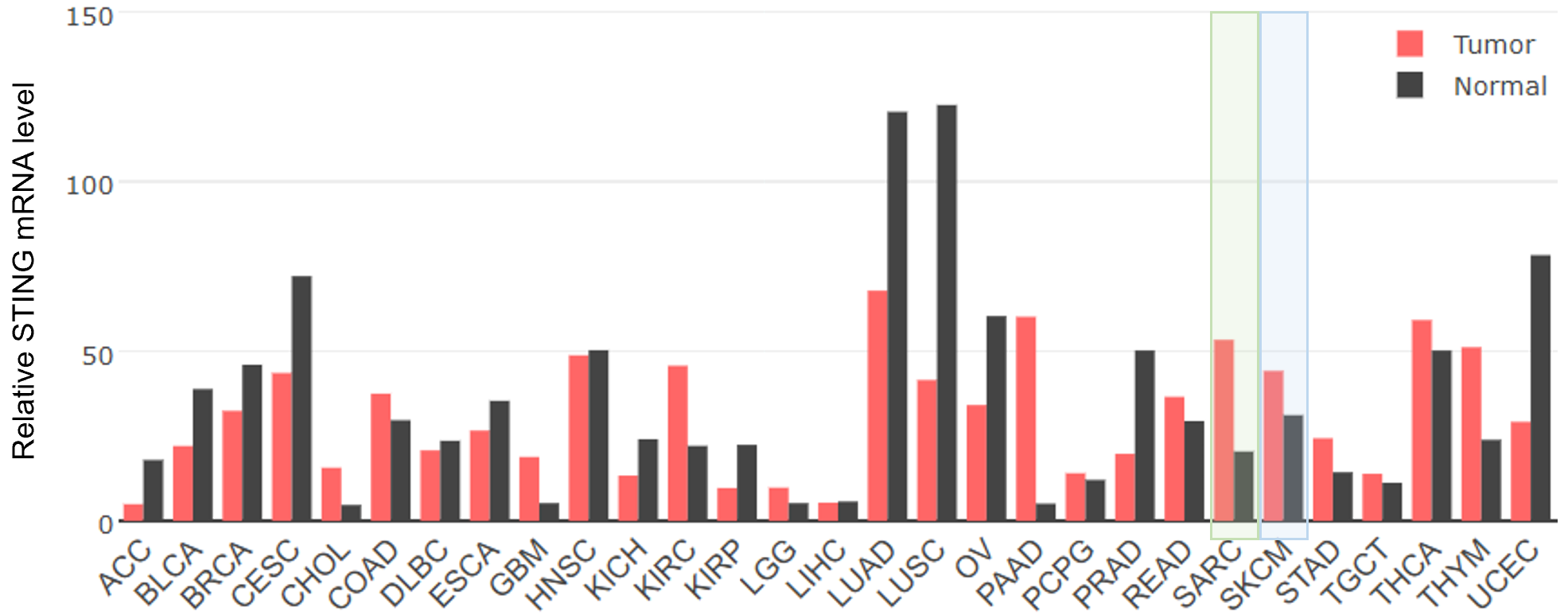


**Fig. S1.** Relative STING mRNA levels in tumor and adjacent normal tissues. Data were extracted from TCGA (The Cancer Genome Atlas) database. (ACC: Adrenocortical carcinoma; BLCA: Bladder urothelial carcinoma; BRCA: Breast invasive carcinoma; CESC: Cervical squamous cell carcinoma and endocervical adenocarcinoma; CHOL: Cholangiocarcinoma; COAD: Colon adenocarcinoma; DLBC: Diffuse Large B-cell Lymphoma; ESCA: Esophageal carcinoma; GBM: Glioblastoma multiforme; HNSC: Head and neck squamous cell carcinoma; KICH: Kidney Chromophobe; KIRC: Kidney renal clear cell carcinoma; KIRP: Kidney renal papillary cell carcinoma; LGG: Lower grade glioma; LIHC: Liver hepatocellular carcinoma; LUAD: Lung adenocarcinoma; LUSC: Lung squamous cell carcinoma; OV: Ovarian serous cystadenocarcinoma; PAAD: Pancreatic adenocarcinoma; PCPG: Pheochromocytoma and Paraganglioma; PRAD: Prostate adenocarcinoma; READ: Rectum adenocarcinoma; SARC: Sarcoma; SKCM: Skin cutaneous melanoma; STAD: Stomach adenocarcinoma; TGCT: Testicular germ cell tumors; THCA: Thyroid carcinoma; THYM: Thymoma; UCEC: Uterine corpus endometrial carcinoma).


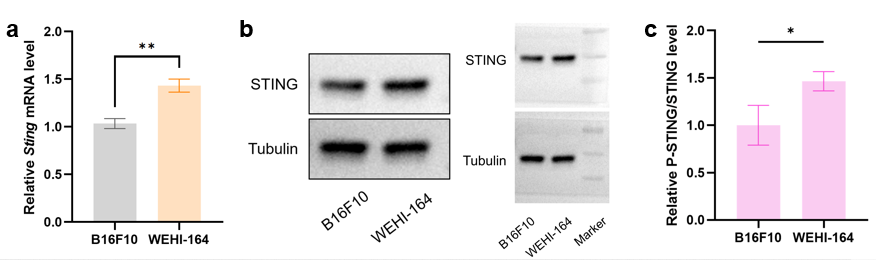


**Fig. S2.** (a) Relative *Sting* mRNA levels in B16F10 and WEHI-164 cells determined by qPCR. (b) Western blot analysis and corresponding uncropped original blot images of STING and Tubulin. (c) Semi-quantitative densitometric analysis of relative p-STING/STING levels. Data are presented as mean ± SD (n=3). **p* < 0.05; ***p* < 0.01.


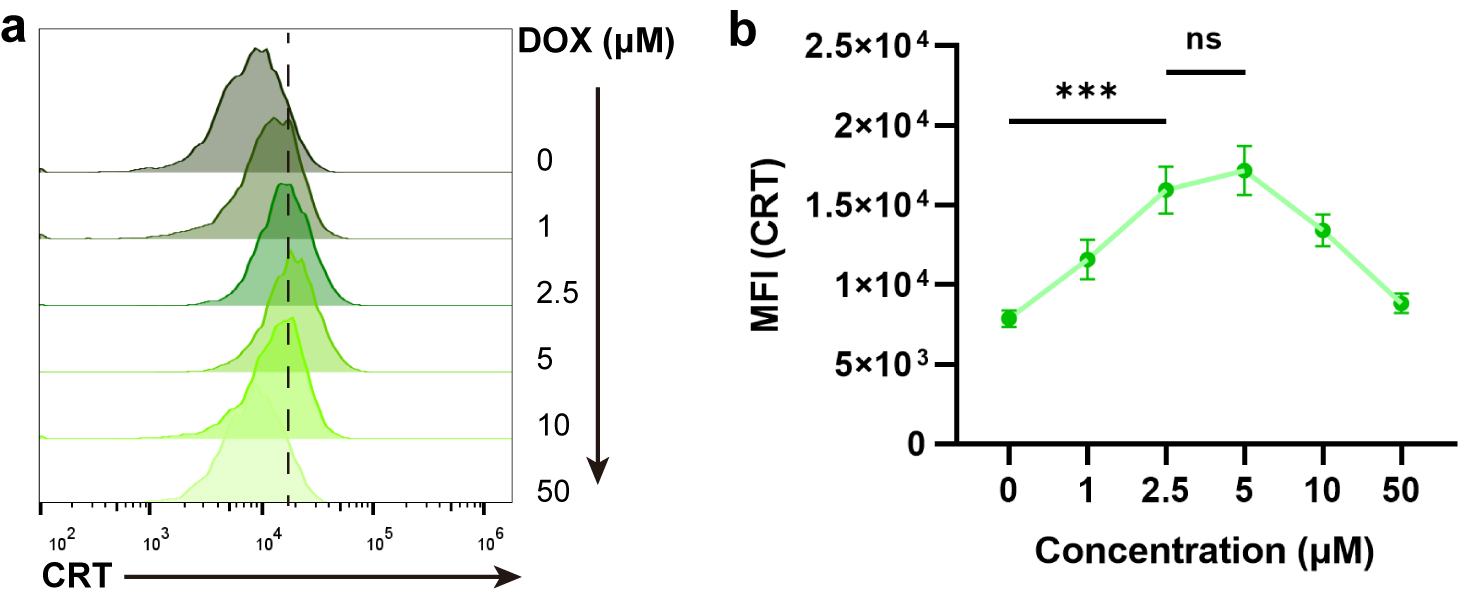


**Fig. S3.** Flow cytometry profiles (a) and quantification (b) of CRT expression on WEHI-164 cell surfaces after 12 h incubation with different concentrations of DOX. Data were presented as mean ± SD (n=3). ns, *p* > 0.05; ****p* < 0.001


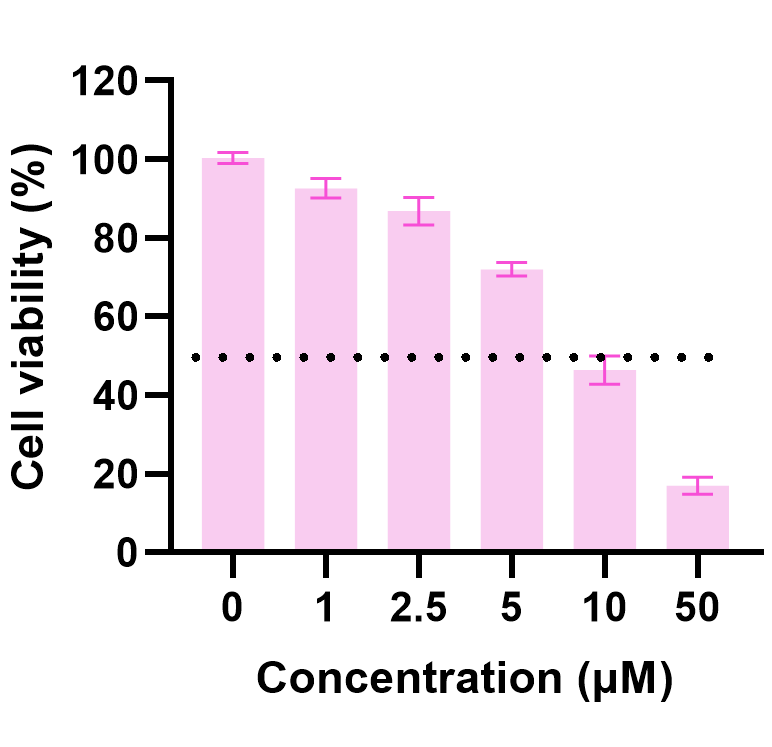


**Fig. S4.** Viability of WEHI-164 cells after 12 h incubation with different doses of DOX. Data were presented as mean ± SD (n=3).


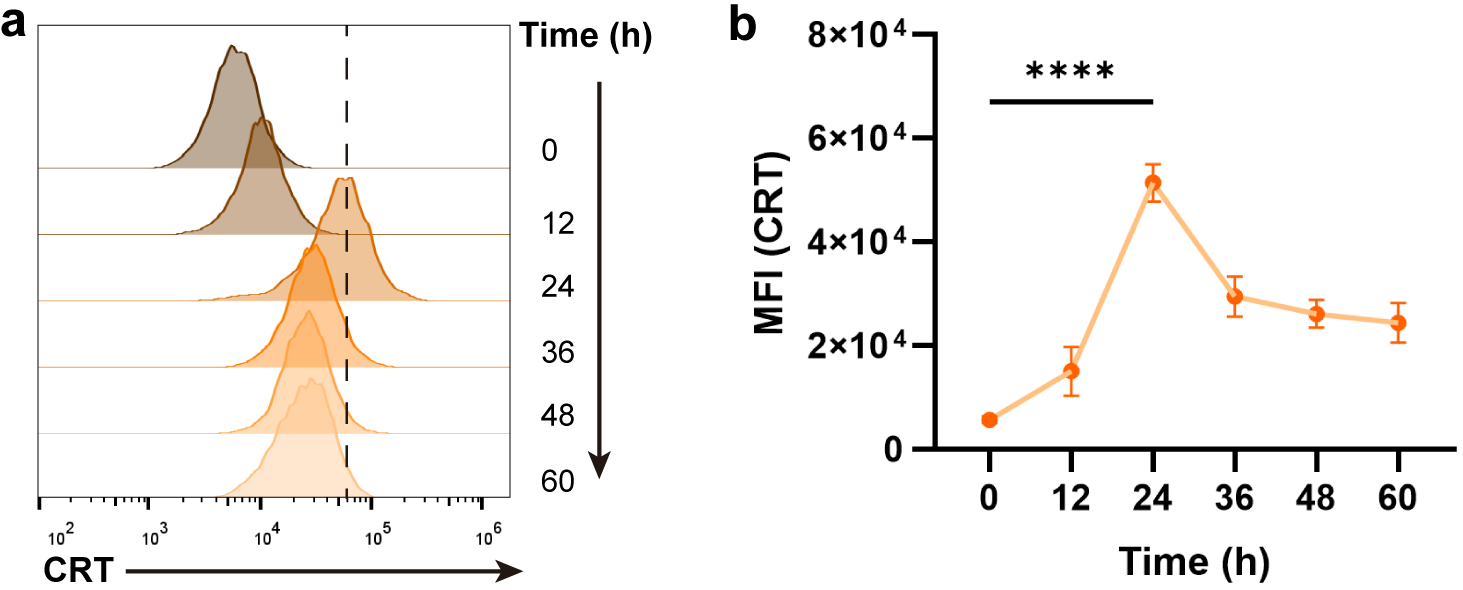


**Fig. S5.** Flow cytometry profiles (a) and quantification (b) of CRT expression on WEHI-164 cell surfaces after incubation with 2.5 μM DOX for different periods. Data were presented as mean ± SD (n=3). *****p* < 0.0001.


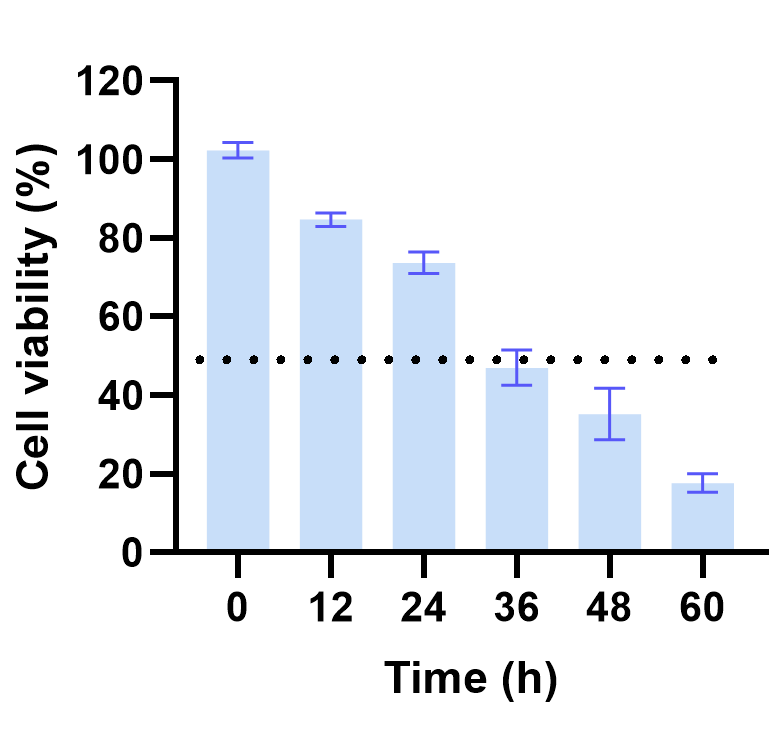


**Fig. S6.** Viability of WEHI-164 cells after incubation with 2.5 μM DOX for different durations. Data were presented as mean ± SD (n=3).


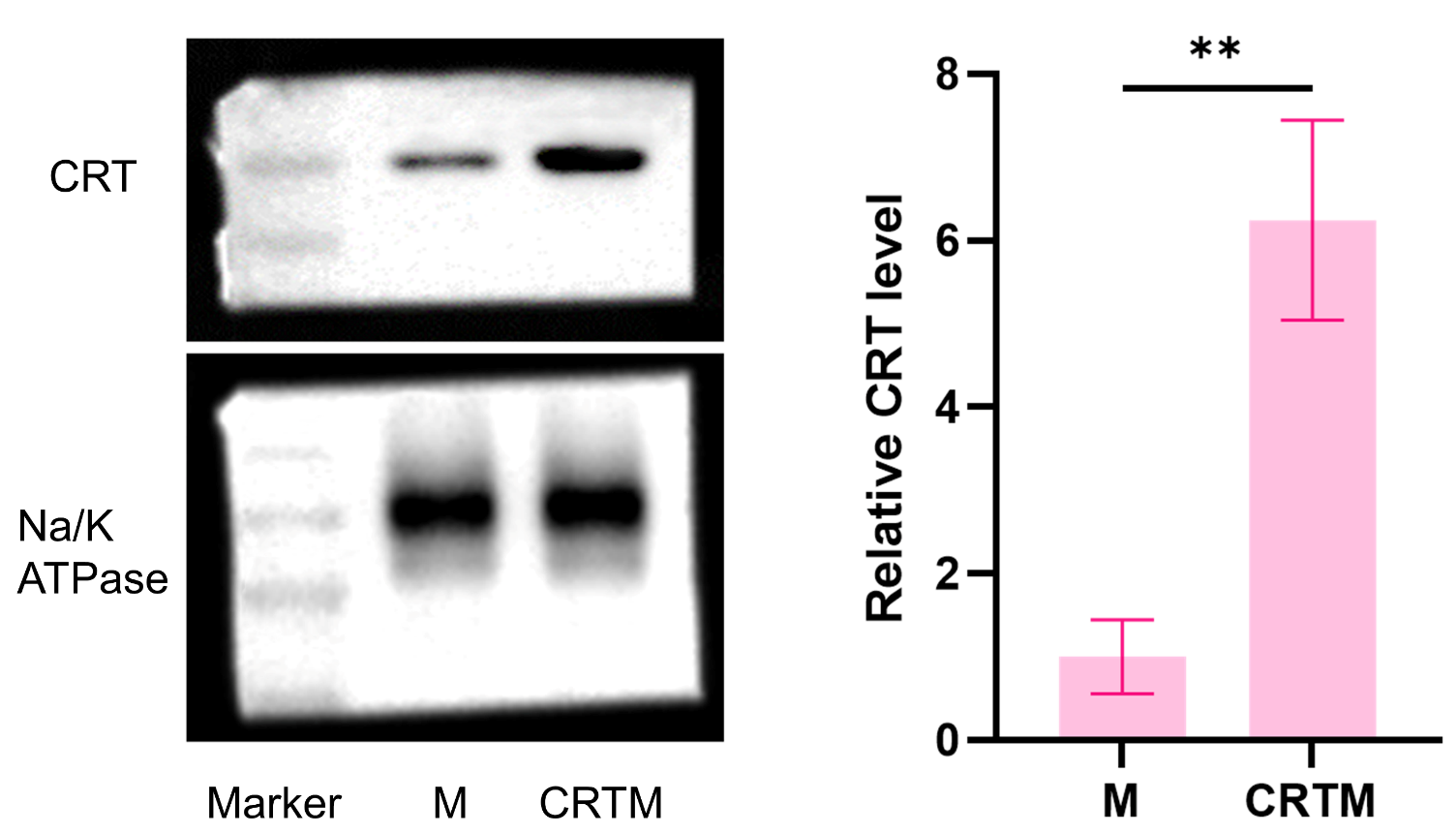


**Fig. S7.** Original uncropped Western blot images and semi-quantitative analysis corresponding to Fig. 1d. Data are presented as mean ± SD (n=3). ***p* < 0.01.

**
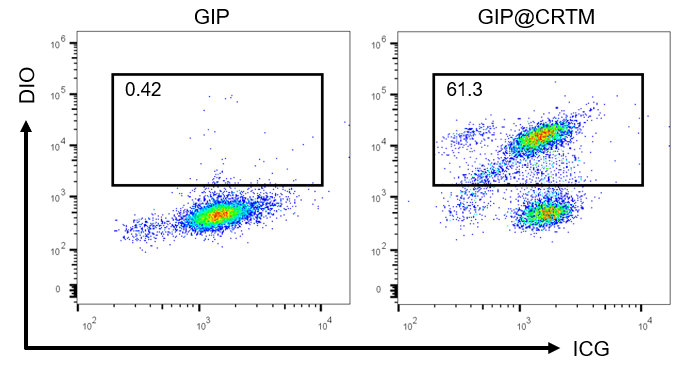
**

**Fig. S8.** Representative nano-flow cytometry dot plots of GIP and GIP@CRTM.


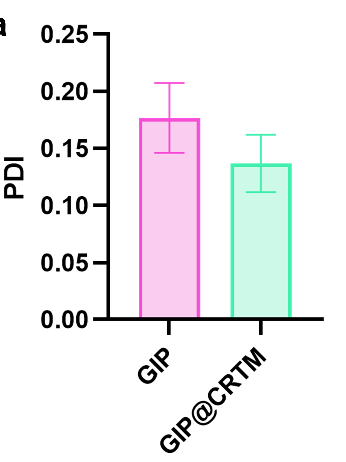


**Fig. S9.** PDI values of freshly prepared GIP and GIP@CRTM. Data were presented as mean ± SD (n=3).


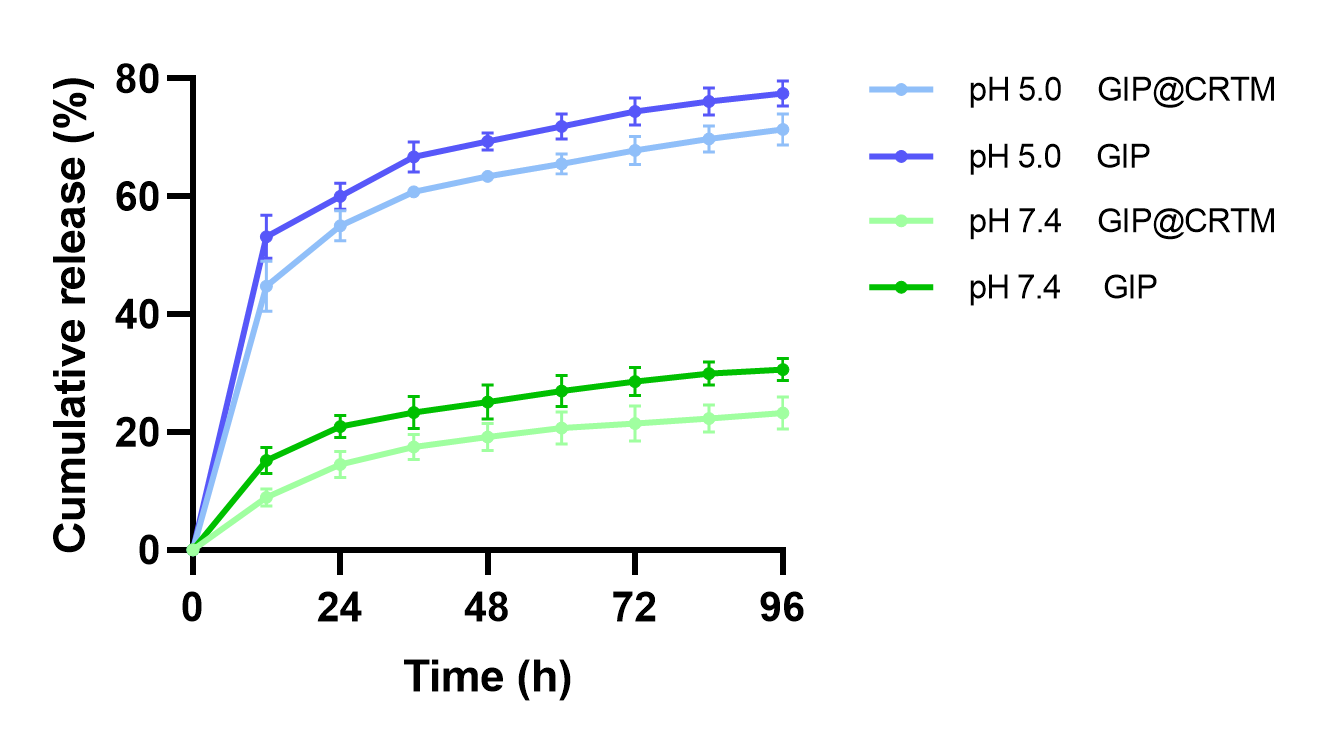


**Fig. S10.** Drug release profiles of GIP@CRTM and GIP under different pH conditions. Data were presented as mean ± SD (n=3).


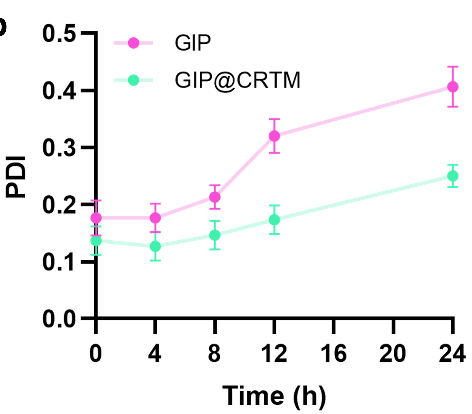


**Fig. S11.** Changes in the PDI values of GIP and GIP@CRTM during 24 h of storage. Data were presented as mean ± SD (n=3).


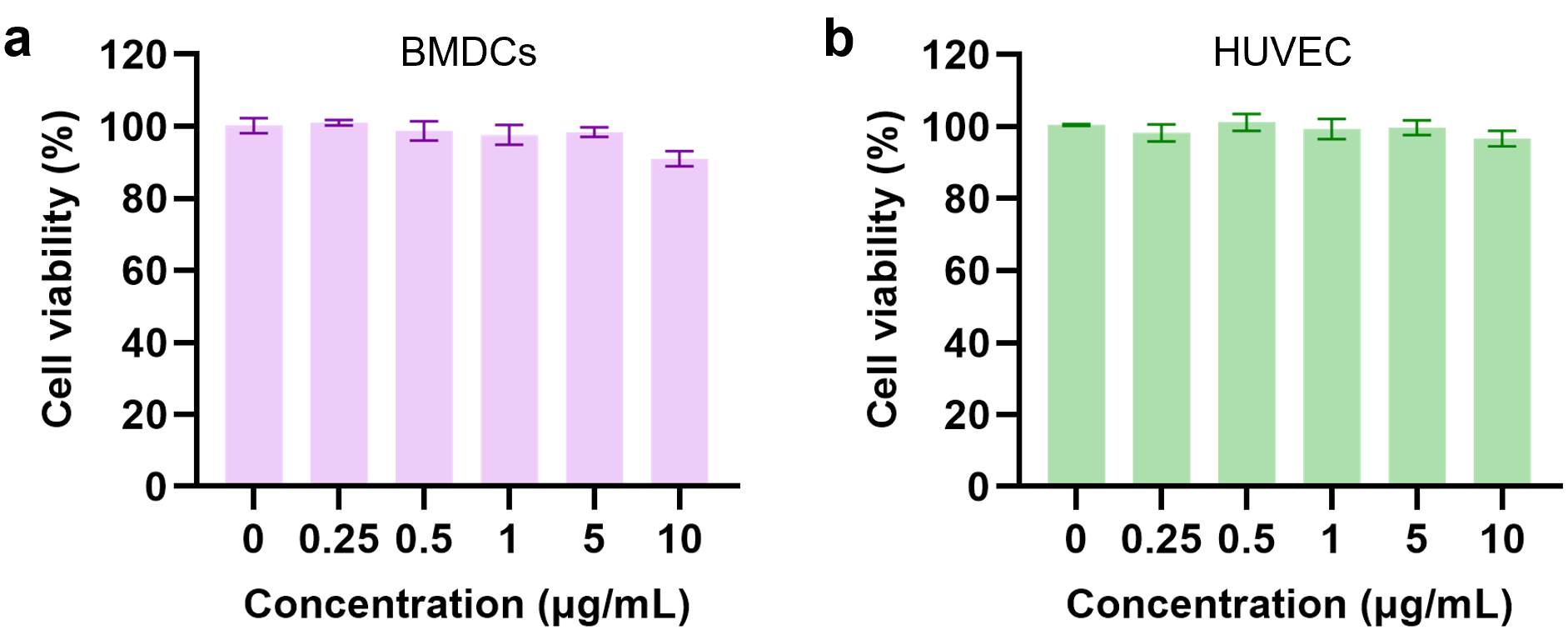


**Fig. S12.** Viability of BMDCs (a) and HUVECs (b) treated with GIP@CRTM containing different concentrations of cGAMP (0－10 μg/mL). Data were presented as mean ± SD (n=3).


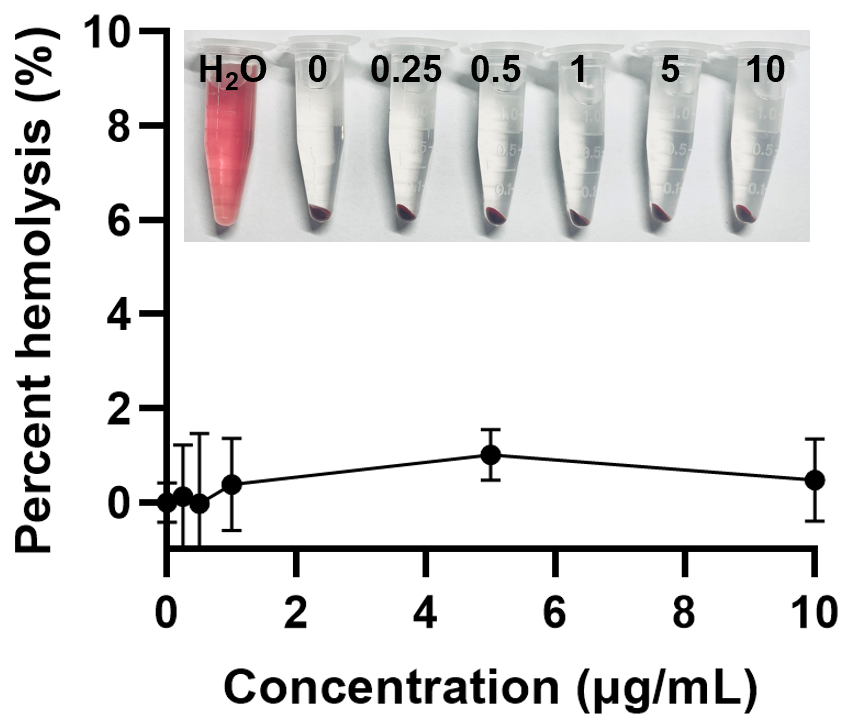


**Fig. S13.** Hemolytic activity of GIP@CRTM loaded with different concentrations of cGAMP (0, 0.25, 0.5, 1, 5, and 10 μg/mL). Data were presented as mean ± SD (n=3).


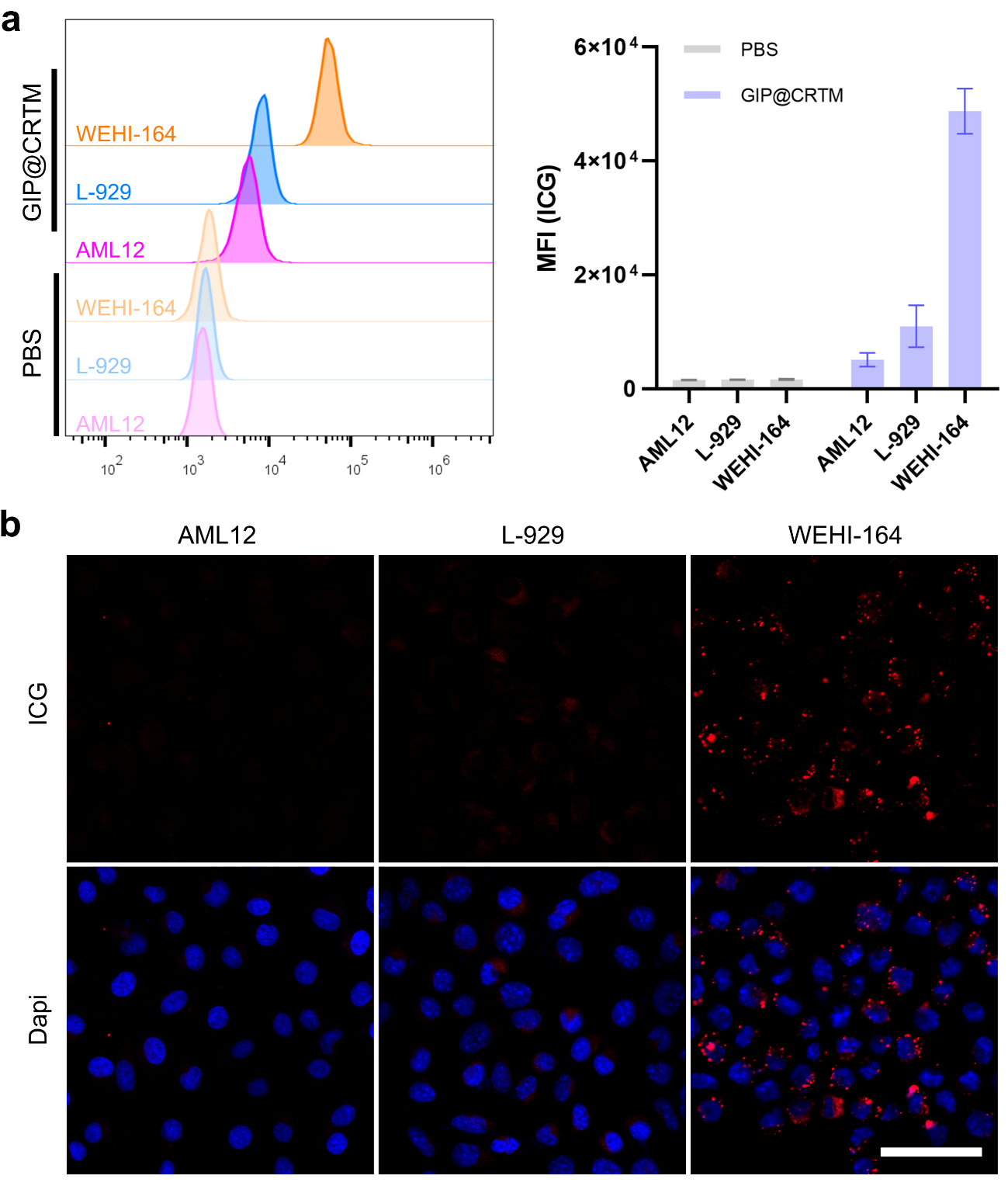


**Fig. S14. Selective uptake of GIP@CRTM by homologous WEHI-164 tumor cells.** (a) Flow cytometric analysis and MFI quantification of ICG fluorescence in AML12, L929, and WEHI-164 cells after incubation with GIP@CRTM. (b) Confocal fluorescence images of GIP@CRTM uptake in AML12, L929, and WEHI-164 cells. Scale bar = 50 µm. Red, ICG; blue, DAPI. Data are presented as mean ± SD (n=3).


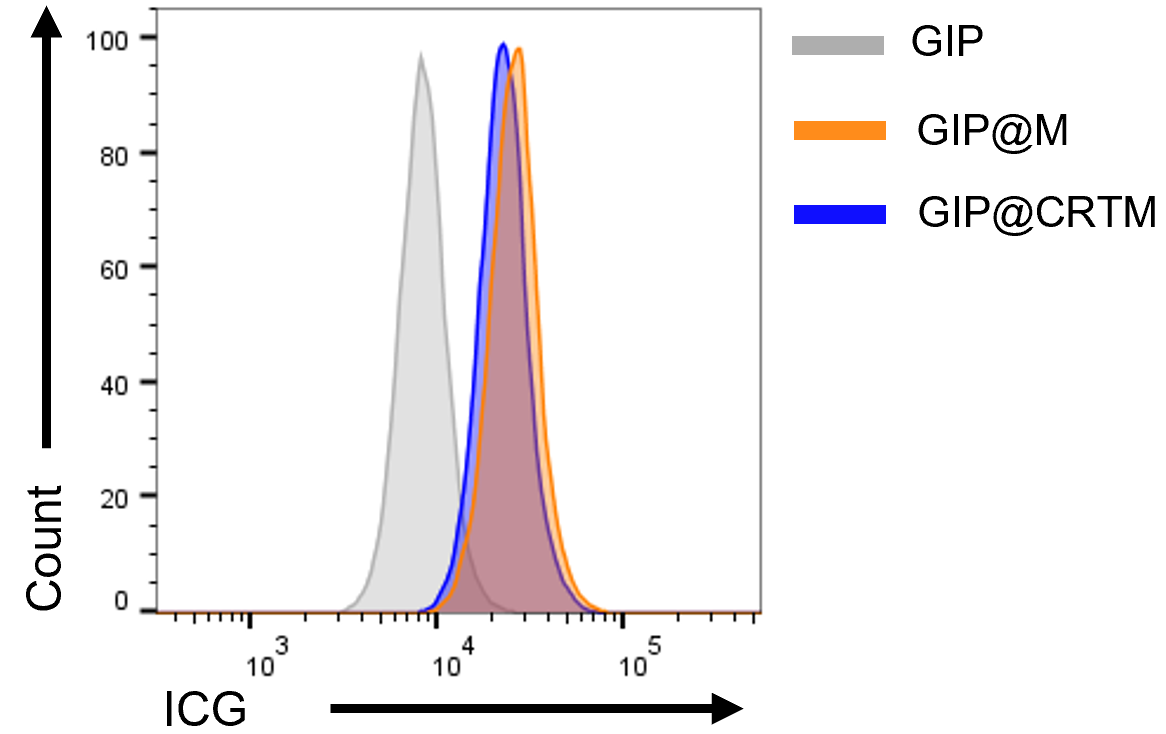


**Fig. S15.** Flow cytometry of WEHI-164 cells treated with GIP, GIP@M or GIP@CRTM for 6 h.


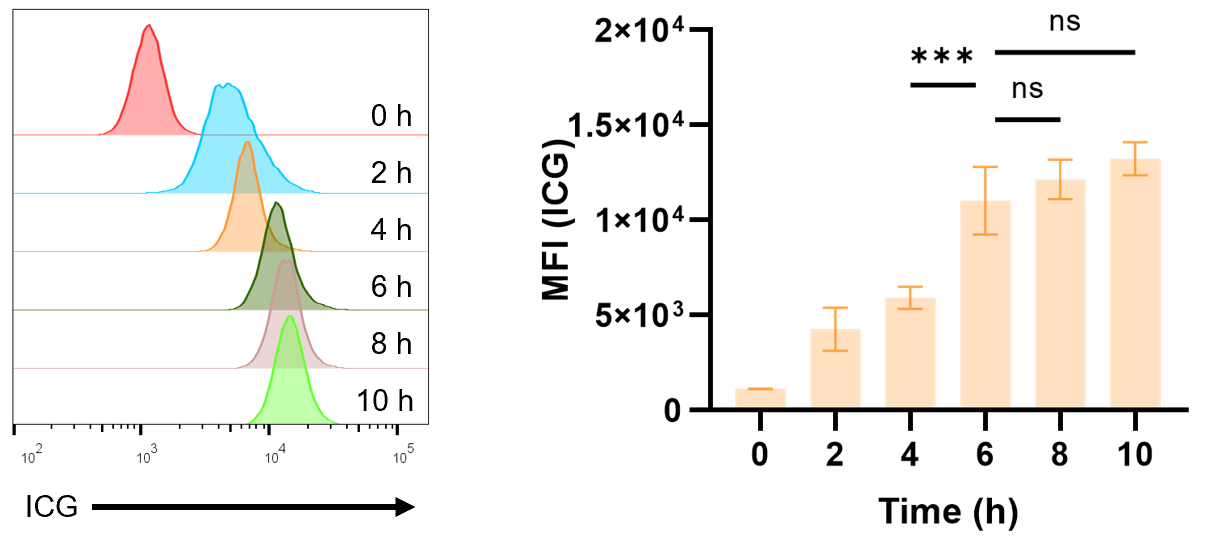


**Fig. S16.** Flow cytometry quantification of GIP@CRTM internalization in WEHI-164 cells over 0－10 h. Data were presented as mean ± SD (n=3). ns, *p* > 0.05; ****p* < 0.001.


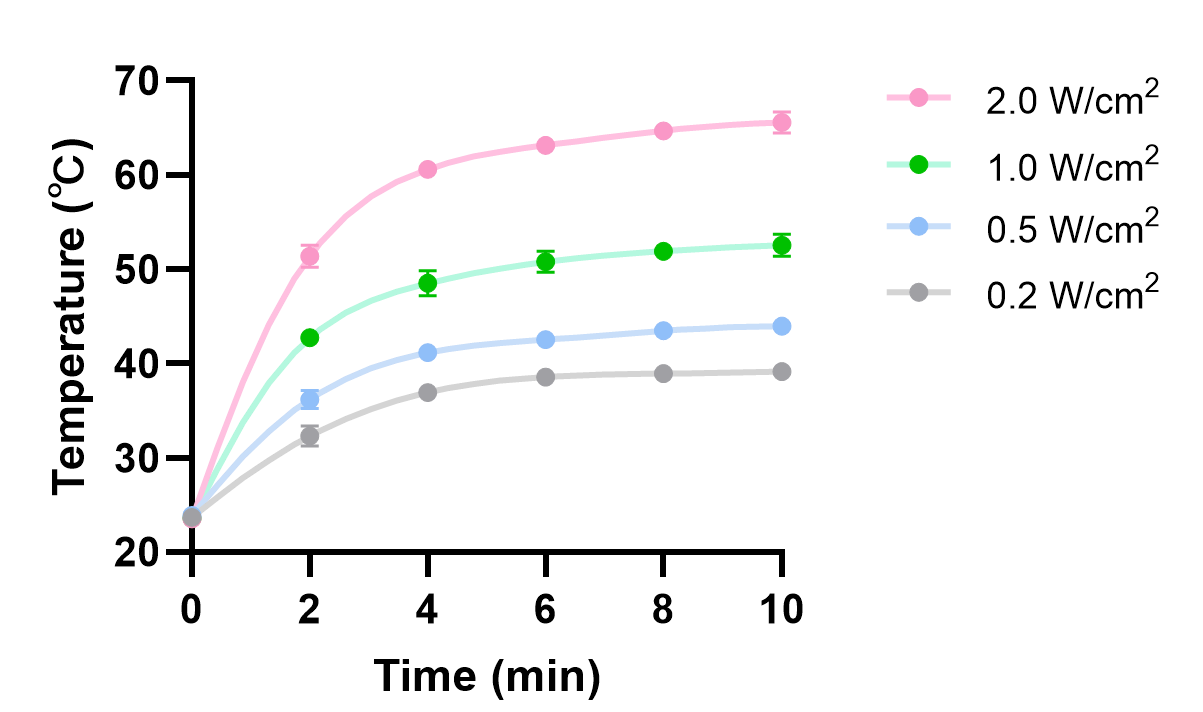


**Fig. S17.** **Temperature curves of** WEHI-164 cells incubated with **GIP@CRTM** (*C*_cGAMP_ = 3 μg/mL, *C*_ICG_ = 18.6 μg/mL) **under 808 nm laser irradiation at different power densities (2.0, 1.0, 0.5, 0.2 W/cm^2^).** Data were presented as mean ± SD (n=3).


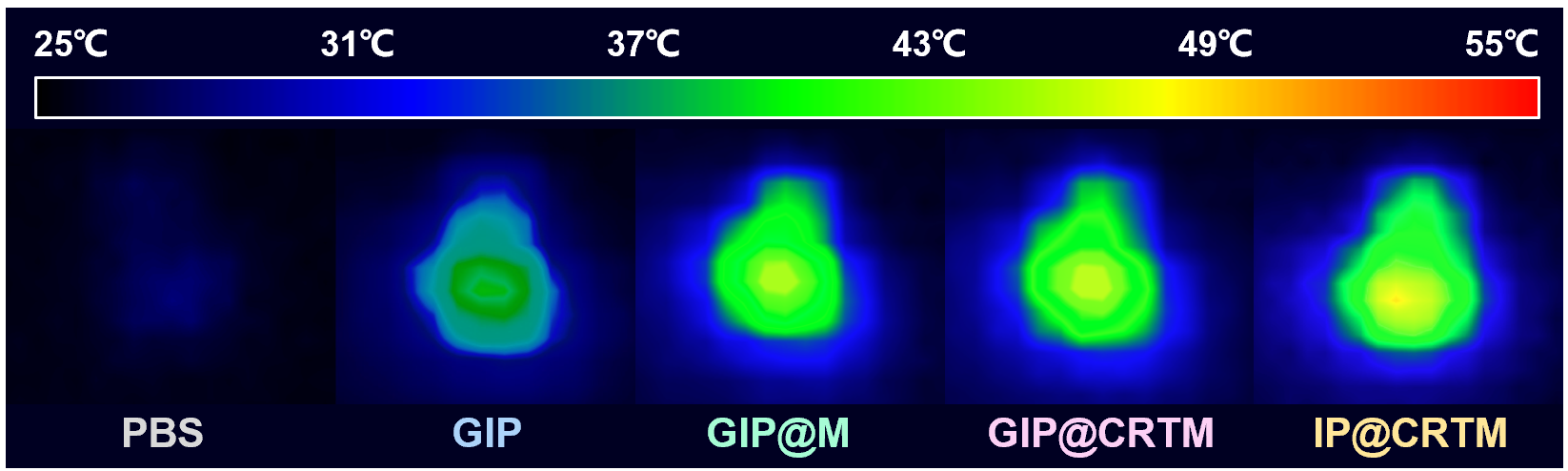


**Fig. S18.** Infrared thermographic images of WEHI-164 cells after 6 h nanoparticle incubation followed by 8 min laser irradiation. (*C*_cGAMP_ = 3 μg/mL, *C*_ICG_ = 18.6 μg/mL)


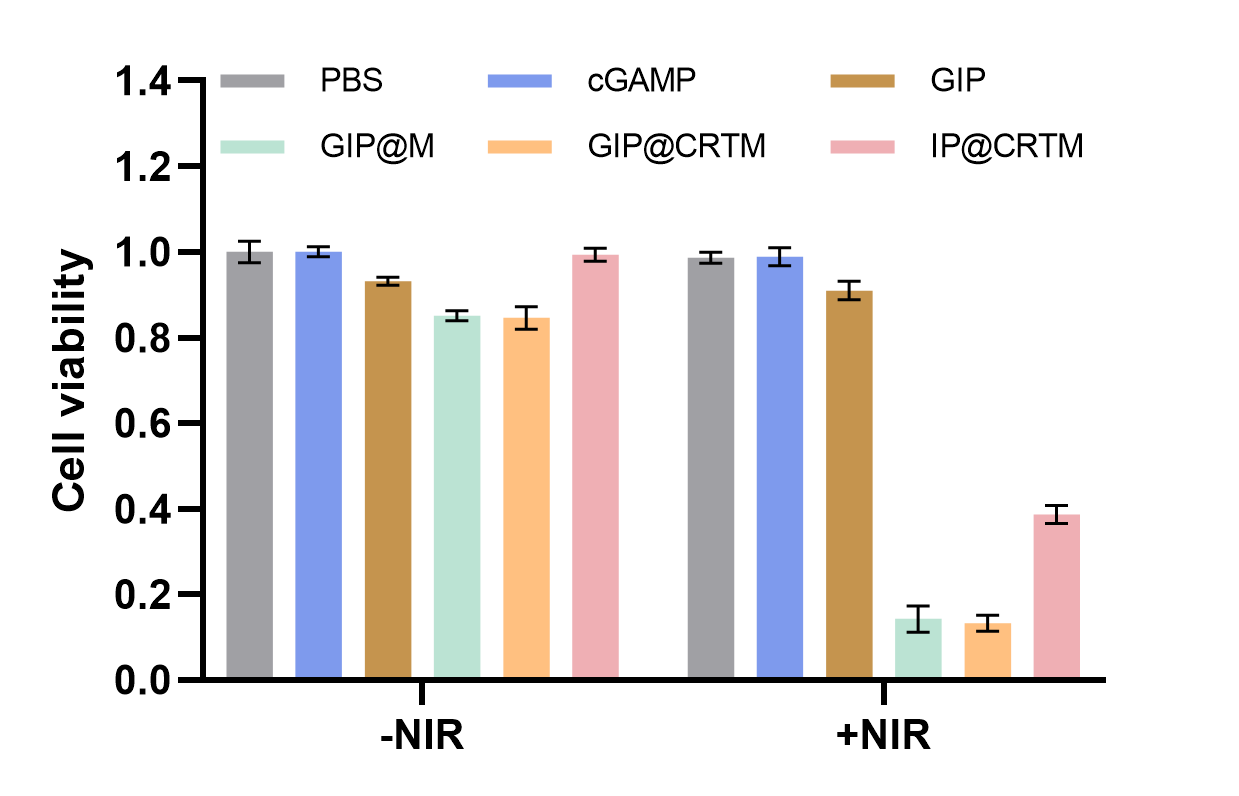


**Fig. S19.** Viability of WEHI-164 cells after different treatments. Data were presented as mean ± SD (n=3).


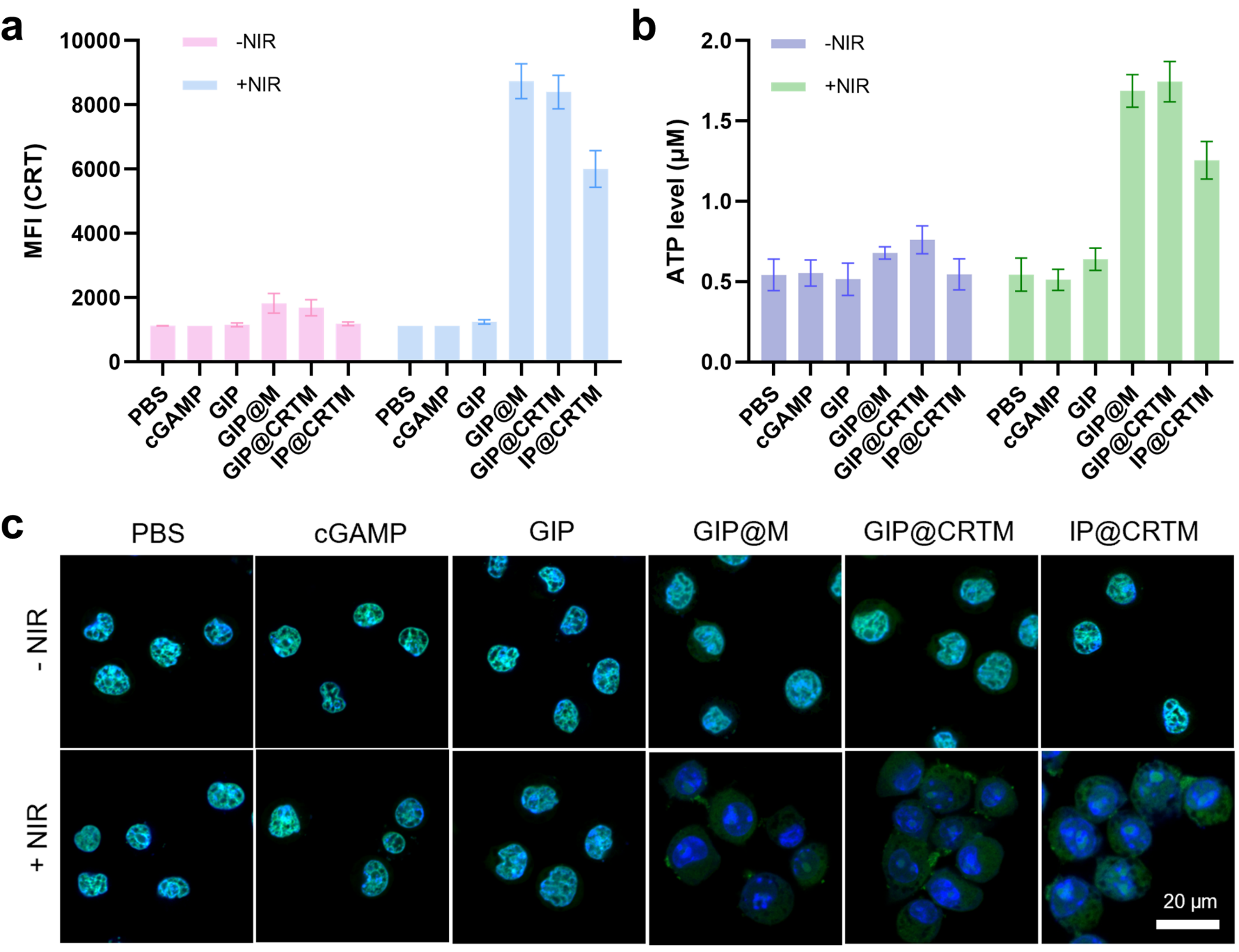


**Fig. S20.** In vitro ICD induction. (a) Flow cytometry quantification of CRT exposure on WEHI-164 cell surfaces after different treatments. (b) ATP release in the supernatant of WEHI-164 cells under various treatments. (c) CLSM images of HMGB1 exposure in WEHI-164 cells after different treatments. Scale bar = 20 μm. Data were presented as mean ± SD (n=3).


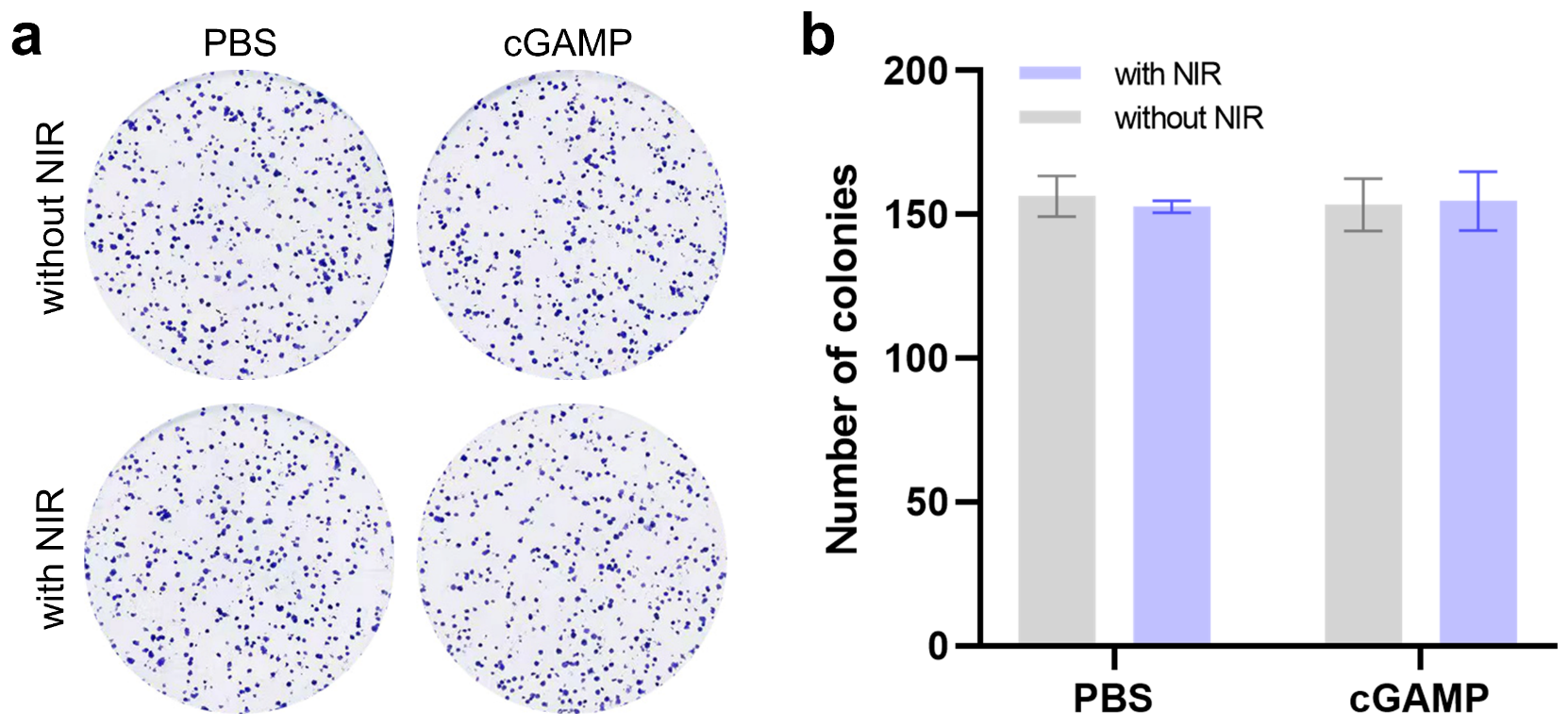


**Fig. S21.** **Effect of free cGAMP on the colony-forming ability of WEHI-164 cells. (a)** Representative colony formation images of WEHI-164 cells treated with PBS or free cGAMP with or without NIR irradiation. **(b)** Quantification of colony numbers in different groups. Data are presented as mean ± SD (n=3).


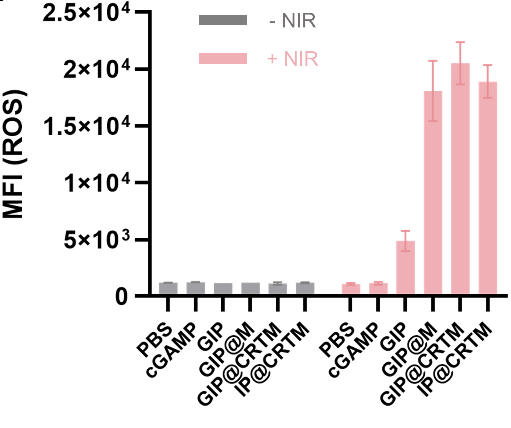


**Fig. S22.** Flow cytometry of ROS generation in WEHI-164 cells with various treatments. Data were presented as mean ± SD (n=3).


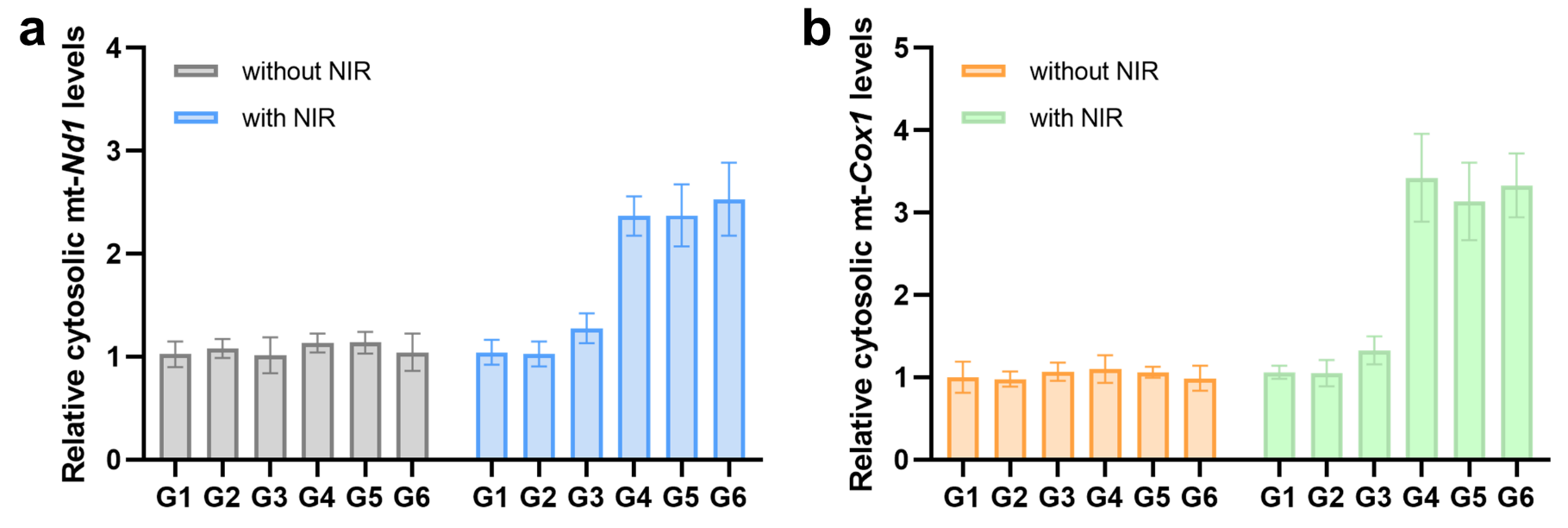


**Fig. S23.** Relative cytosolic levels of mitochondrial genes mt-Nd1 and mt-CoX1 in WEHI-164 cells after different treatments. G1: PBS, G2: cGAMP, G3: GIP, G4: GIP@M, G5: GIP@CRTM, G6: IP@CRTM. Data are presented as mean ± SD (n=3).


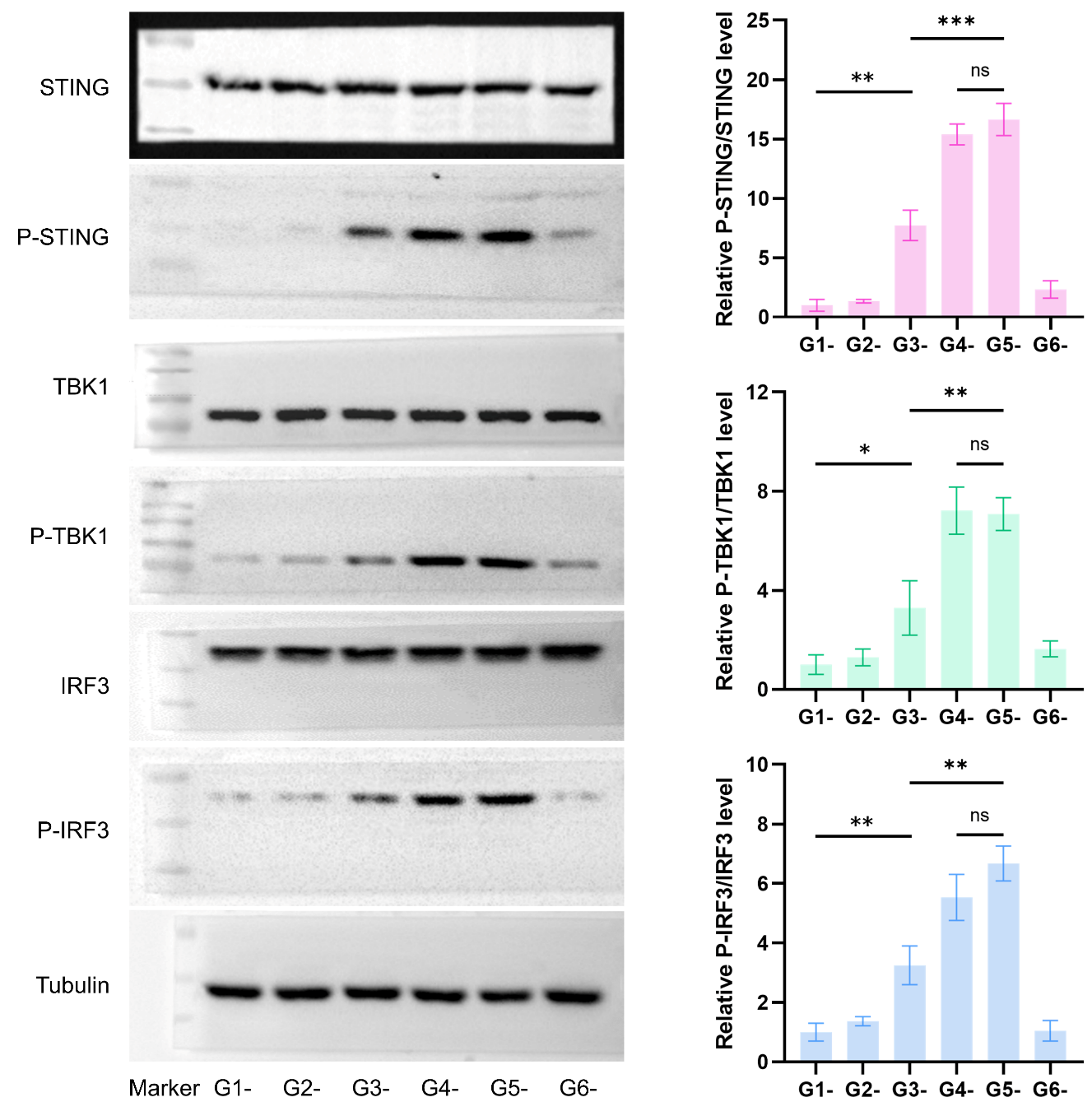


**Fig. S24.** Original uncropped Western blot images and semi-quantitative analysis corresponding to Fig. 2l. Data are presented as mean ± SD (n=3). ns, *p* > 0.05; **p* < 0.1; ***p* < 0.01; ****p* < 0.001.


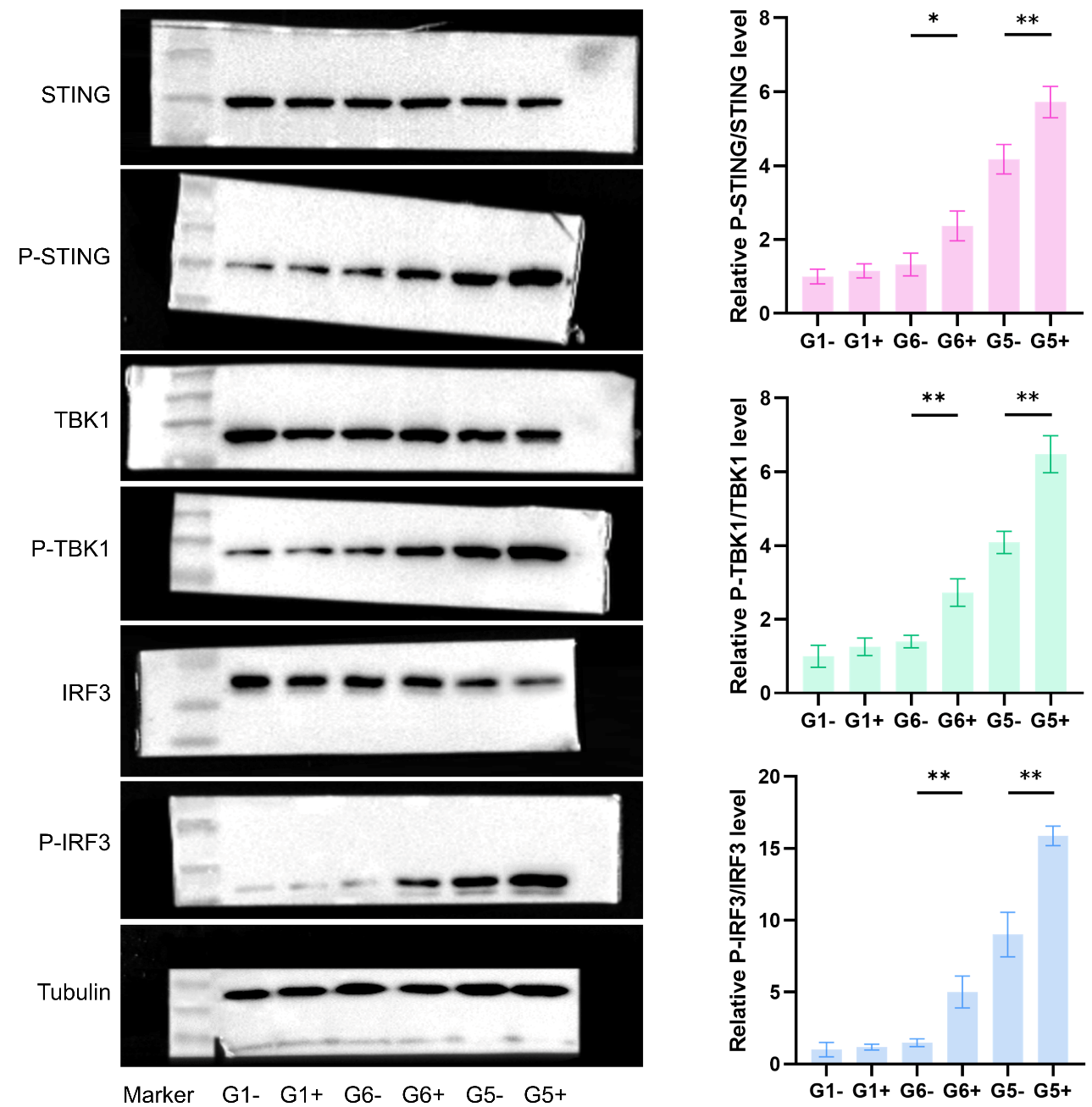


**Fig. S25.** Original uncropped Western blot images and semi-quantitative analysis corresponding to Fig. 2m. Data are presented as mean ± SD (n=3). **p* < 0.1; ***p* < 0.01.


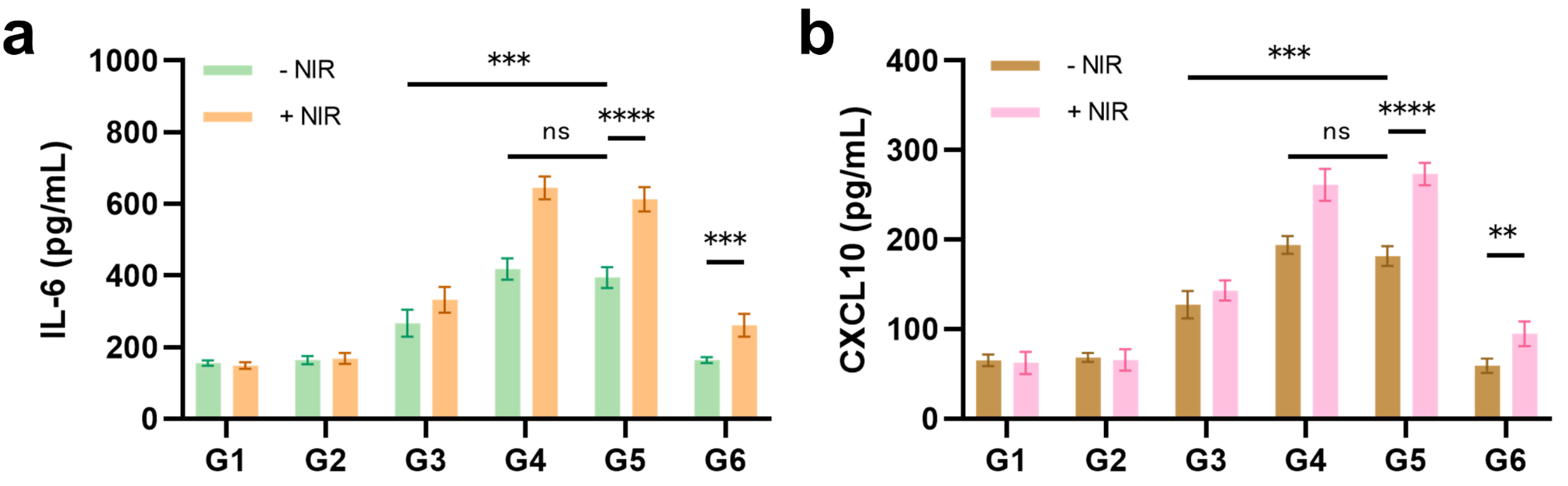


**Fig. S26.** ELISA of (a) IL-6 and (b) CXCL10 in supernatants of WEHI-164 cells under different treatments. G1: PBS, G2: cGAMP, G3: GIP, G4: GIP@M, G5: GIP@CRTM, G6: IP@CRTM. (+): tumor cells with NIR irradiation (808 nm, 0.5 W/cm^2^, 8 min), (-): tumor cells without NIR irradiation. Data were presented as mean ± SD (n=3). ns, *p* > 0.05; ***p* < 0.01; ****p* < 0.001; *****p* < 0.0001.


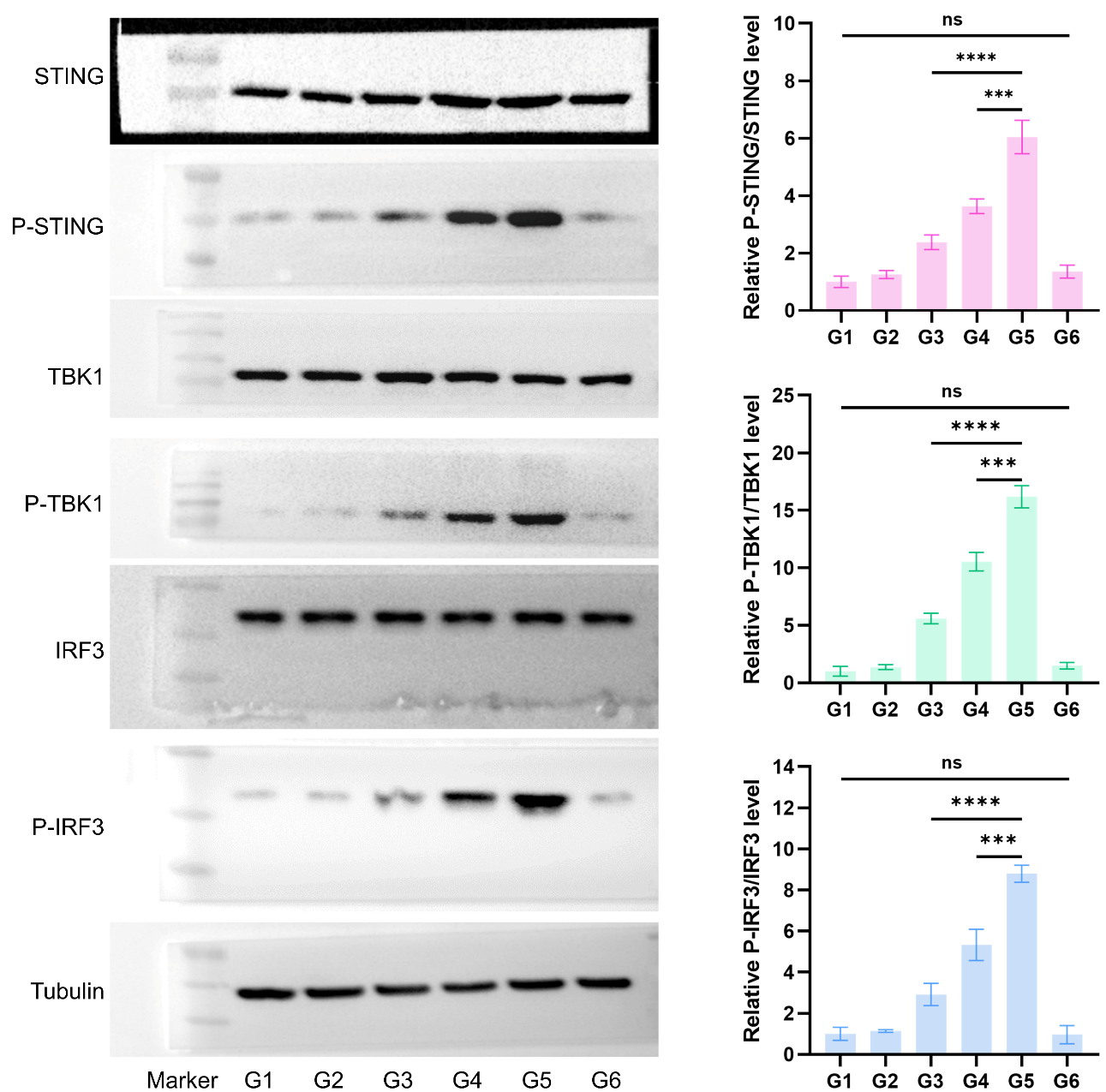


**Fig. S27.** Original uncropped Western blot images and semi-quantitative analysis corresponding to Fig. 3e. Data are presented as mean ± SD (n=3). ****p* < 0.001; *****p* < 0.0001.


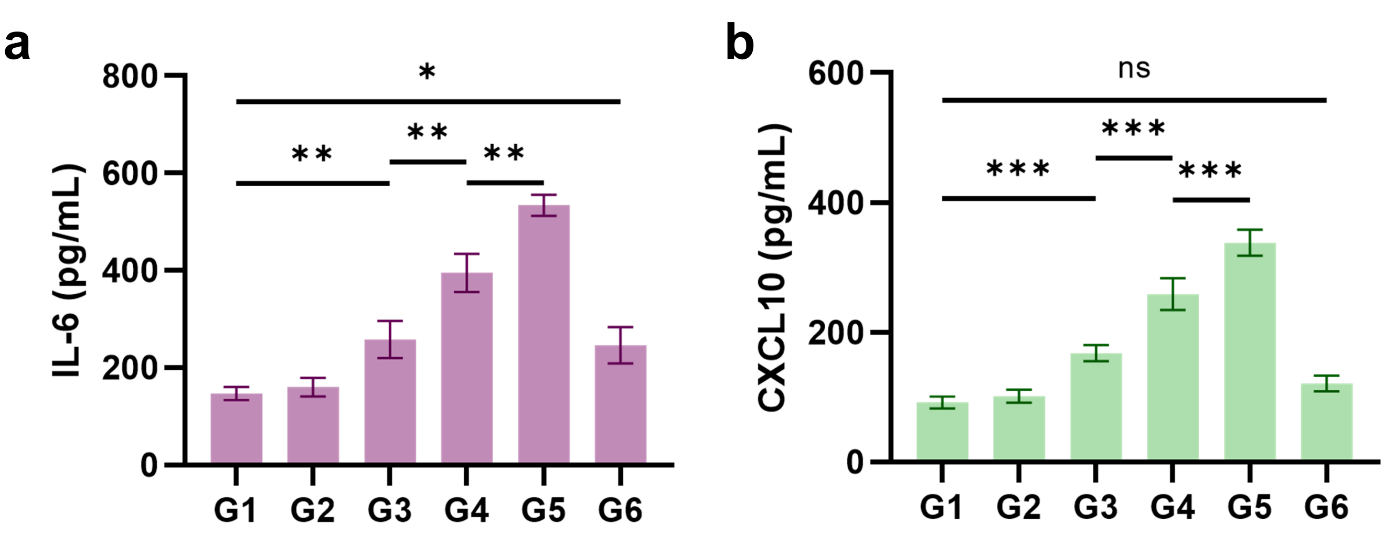


**Fig. S28.** ELISA of (a) IL-6 and (b) CXCL10 in supernatants of BMDCs with different treatments. G1: PBS, G2: cGAMP, G3: GIP, G4: GIP@M, G5: GIP@CRTM, G6: IP@CRTM. Data were presented as mean ± SD (n=3). ns, *p* > 0.05; **p* < 0.05; ***p* < 0.01; ****p* < 0.001.


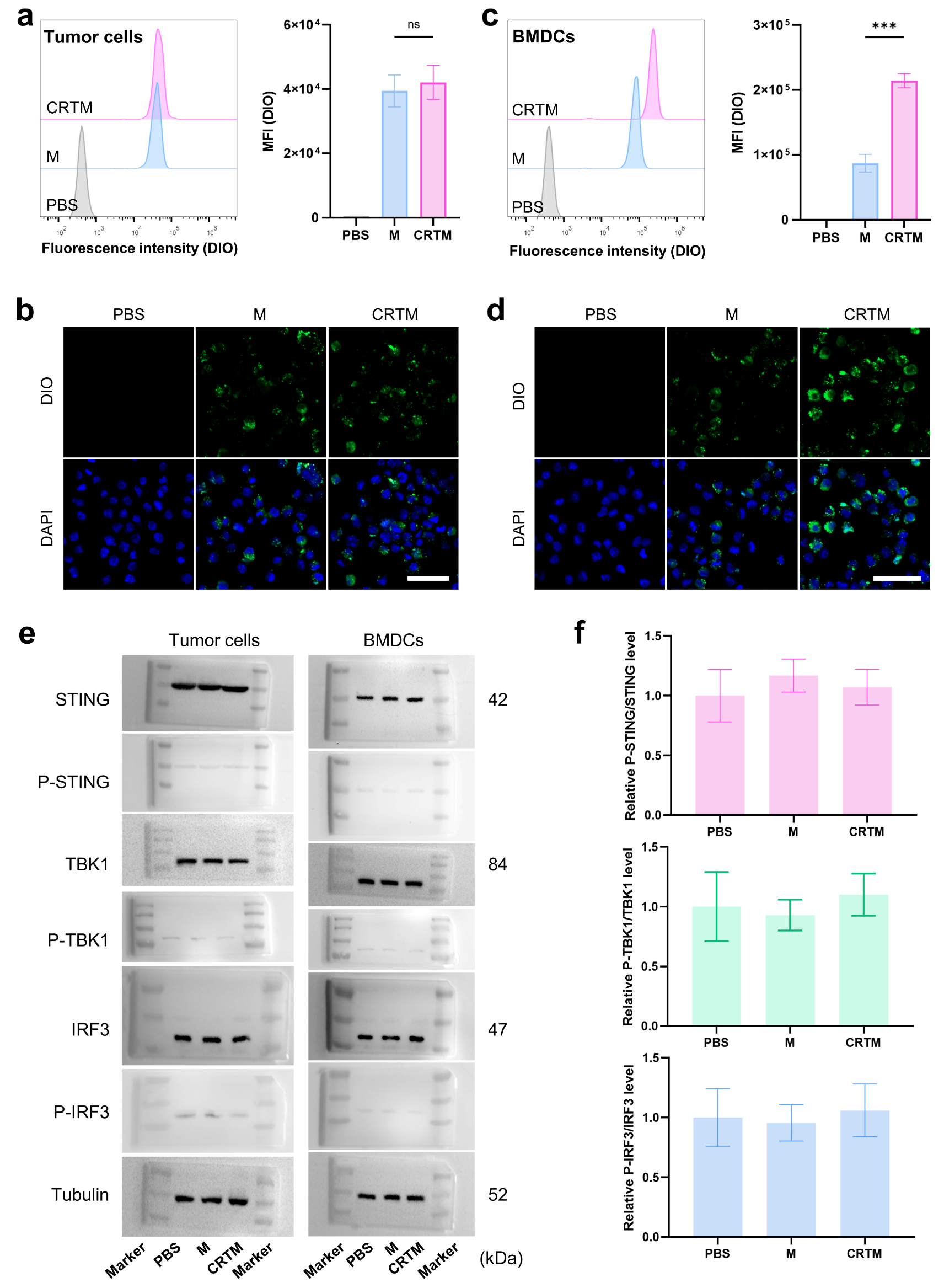


**Fig. S29.** Evaluation of membrane-mediated cellular uptake and STING pathway activation. (a) Flow cytometry and (b) fluorescence imaging of DiO-labeled M and CRTM uptake by homologous WEHI-164 tumor cells. Scale bar = 50 µm. (c) Flow cytometry and (d) fluorescence imaging of DiO-labeled M and CRTM uptake by BMDCs. Scale bar = 50 µm. (e) Western blot analysis and (f) quantification of STING pathway-related proteins in WEHI-164 tumor cells and BMDCs after treatment with PBS, M, or CRTM. Data are presented as mean ± SD (n=3). ns, not significant; ****p* < 0.001.


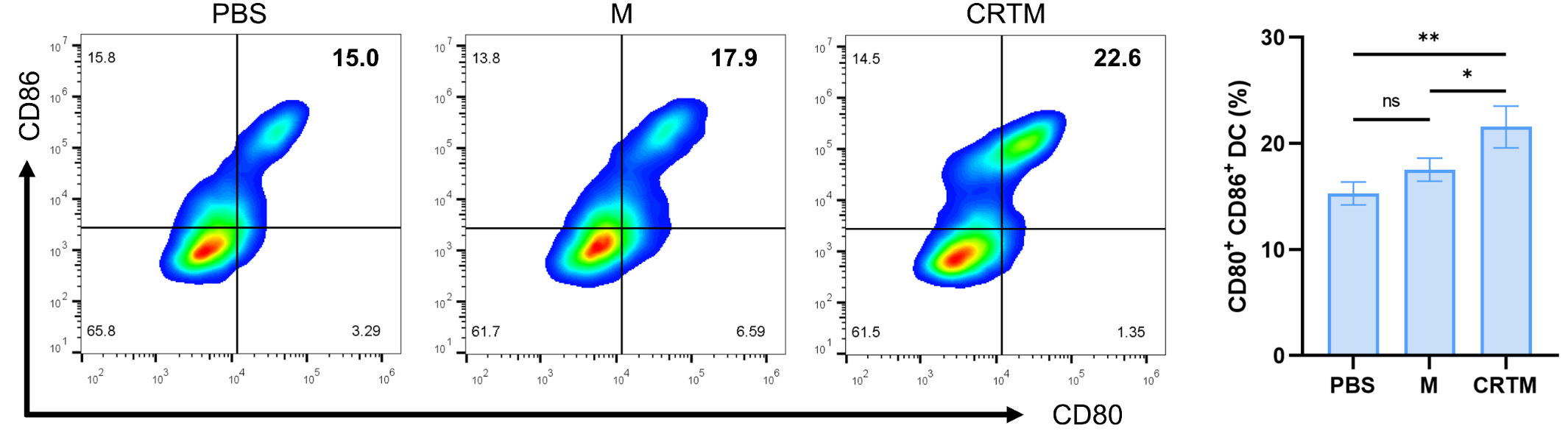


**Fig. S30.** Flow cytometric analysis and quantification of CD80⁺CD86⁺ BMDCs after treatment with PBS, M, or CRTM. Data are presented as mean ± SD (n=3). ns, not significant; **p* < 0.05; ***p* < 0.01.


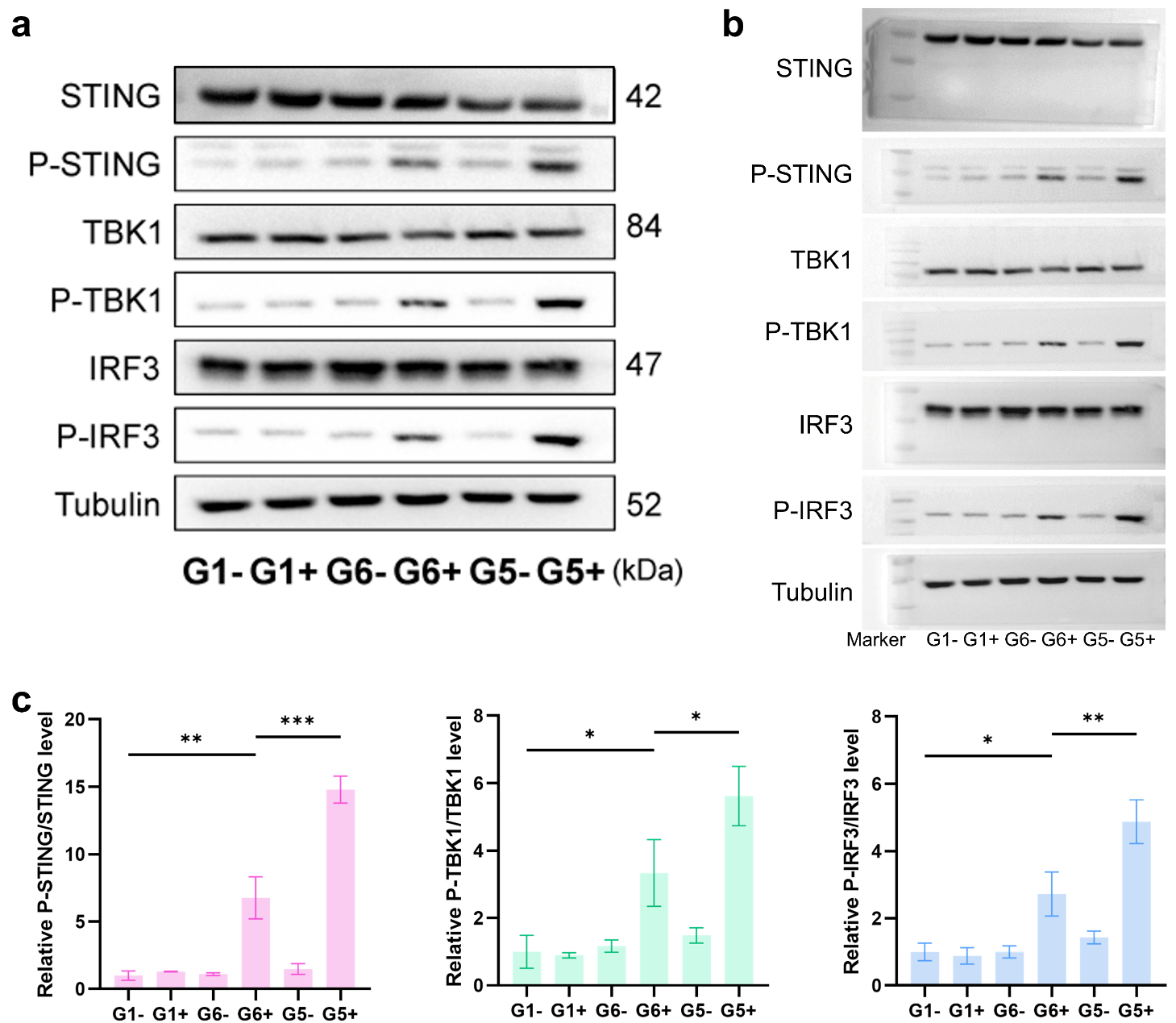


**Fig. S31.** (a) Western blot analysis of STING pathway activation in BMDCs after co-culture with WEHI-164 tumor cells subjected to different treatments. (b) Corresponding uncropped original Western blot images. (c) Semi-quantitative densitometric analysis of relative p-STING/STING, p-TBK1/TBK1, and p-IRF3/IRF3 levels. G1: PBS, G5: GIP@CRTM, G6: IP@CRTM. (+): tumor cells with NIR irradiation (808 nm, 0.5 W/cm^2^, 8 min), (-): tumor cells without NIR irradiation. Data are presented as mean ± SD (n=3). **p* < 0.05; ***p* < 0.01; ****p* < 0.001.


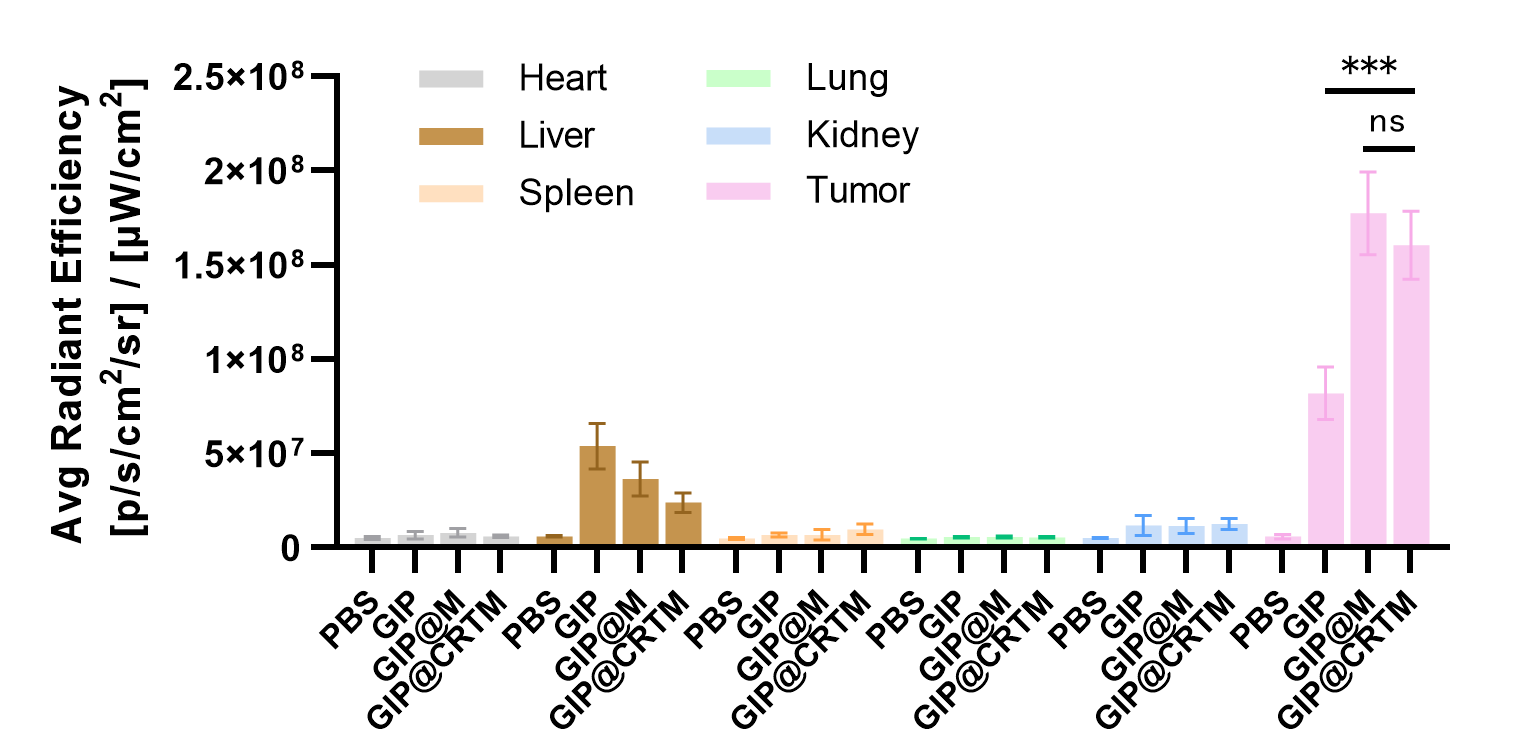


**Fig. S32.** Quantification of fluorescence signals in excised major organs and tumors at 12 h post-intravenous injection of nanoagonists. Data were presented as mean ± SD (n=5). ns, *p* > 0.05; ****p* < 0.001.


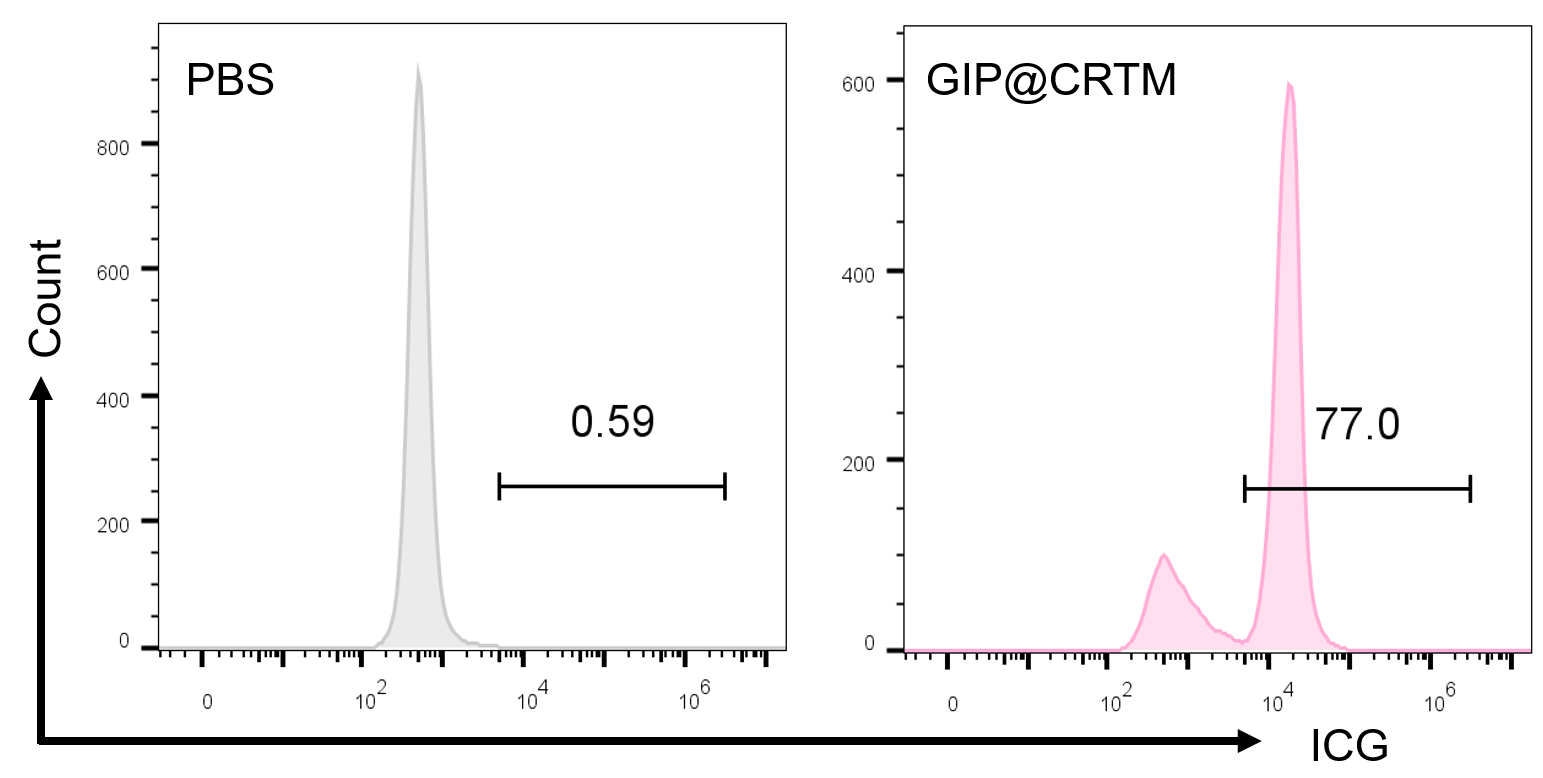


**Fig. S33.** Representative flow cytometry histograms of ICG fluorescence in mature DCs (CD45⁺CD11c⁺ CD80⁺CD86⁺) from tumor-draining inguinal lymph nodes after PBS or GIP@CRTM treatment.


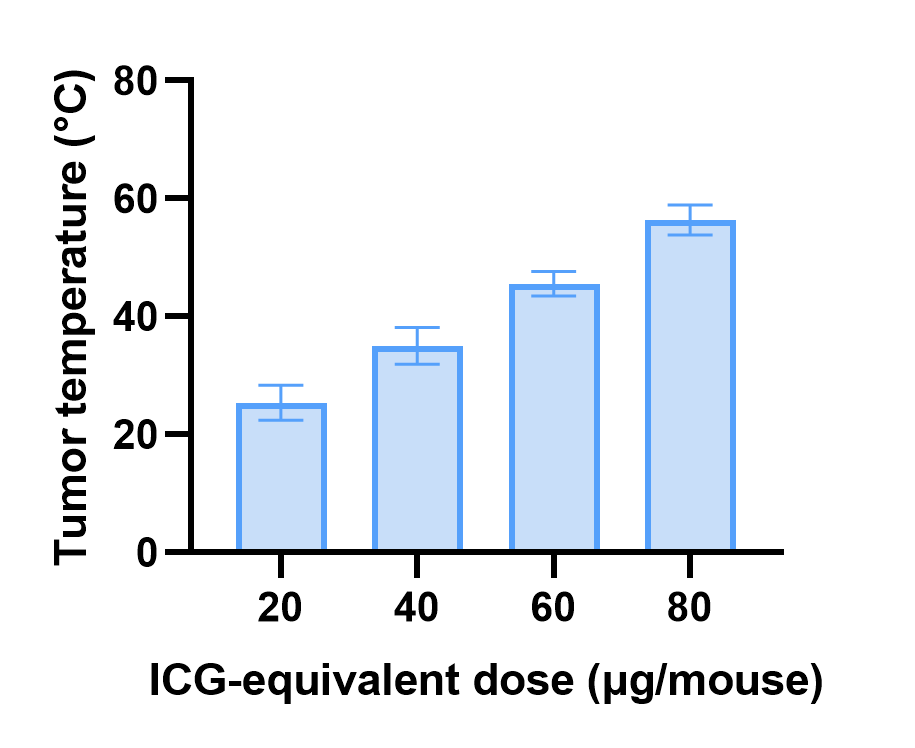


**Fig. S34.** Tumor temperature induced by IP@CRTM at different ICG-equivalent doses under NIR irradiation (808 nm, 0.5 W/cm^2^, 10 min). Data were presented as mean ± SD (n=5).


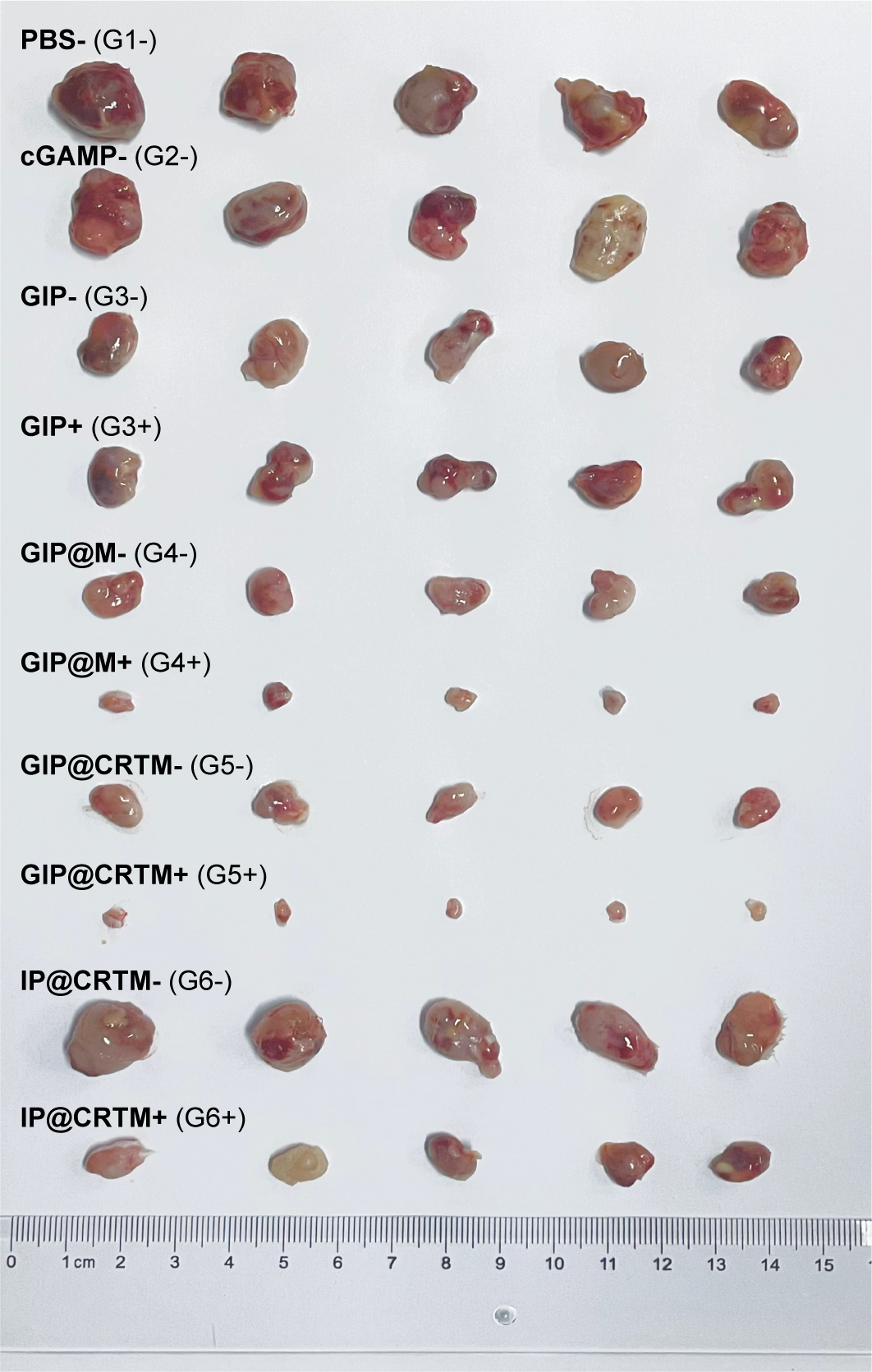


**Fig. S35.** Photographs of excised tumors from each group on day 10 of treatment. (+): tumors with NIR irradiation (808 nm, 0.5 W/cm^2^, 10 min), (-): tumors without NIR irradiation. (n = 5)


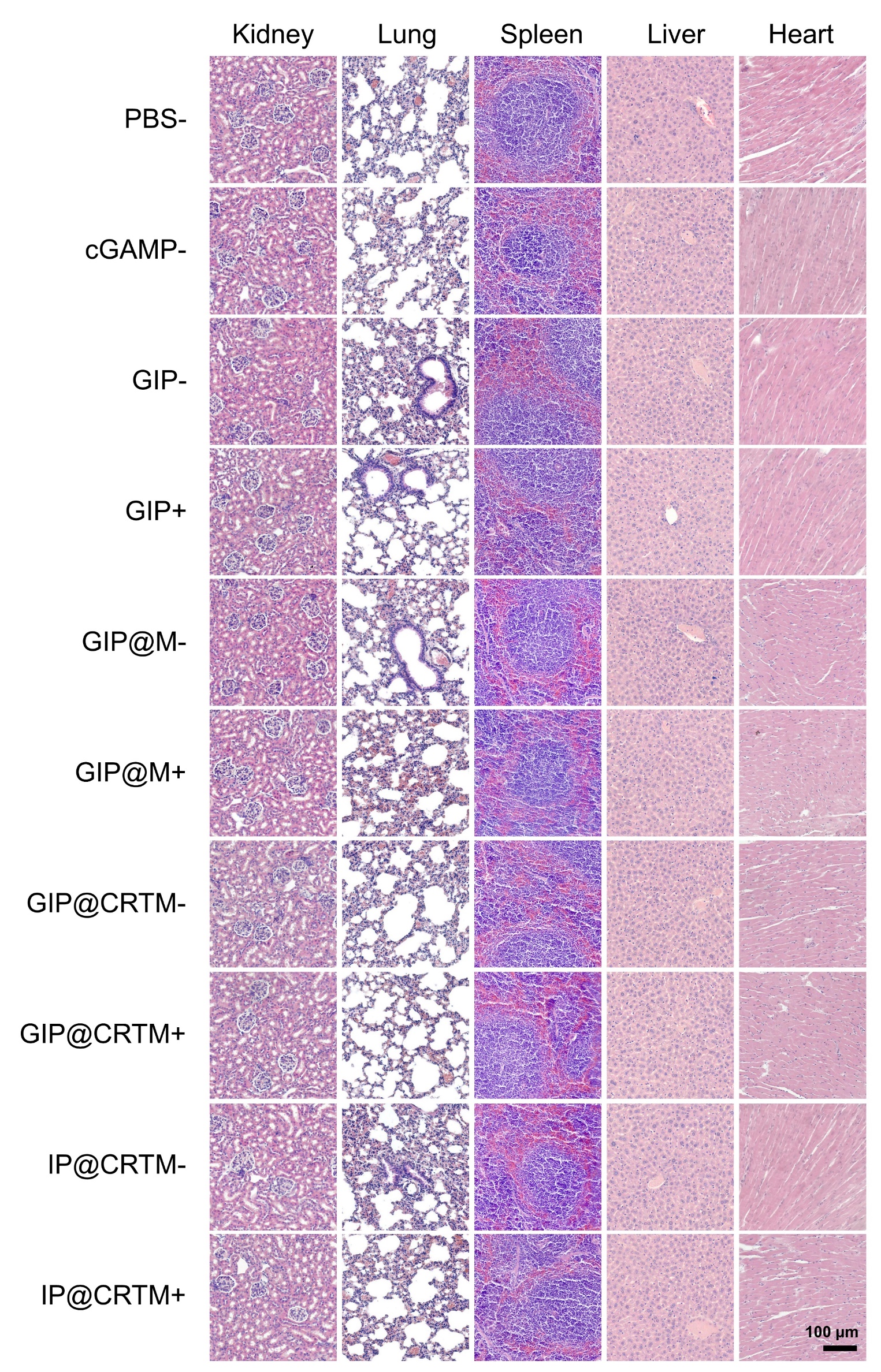


**Fig. S36.** Representative H&E staining of major organ from mice in different treatment groups. Scale bar = 100 μm. (+): tumors with NIR irradiation (808 nm, 0.5 W/cm^2^, 10 min), (-): tumors without NIR irradiation.


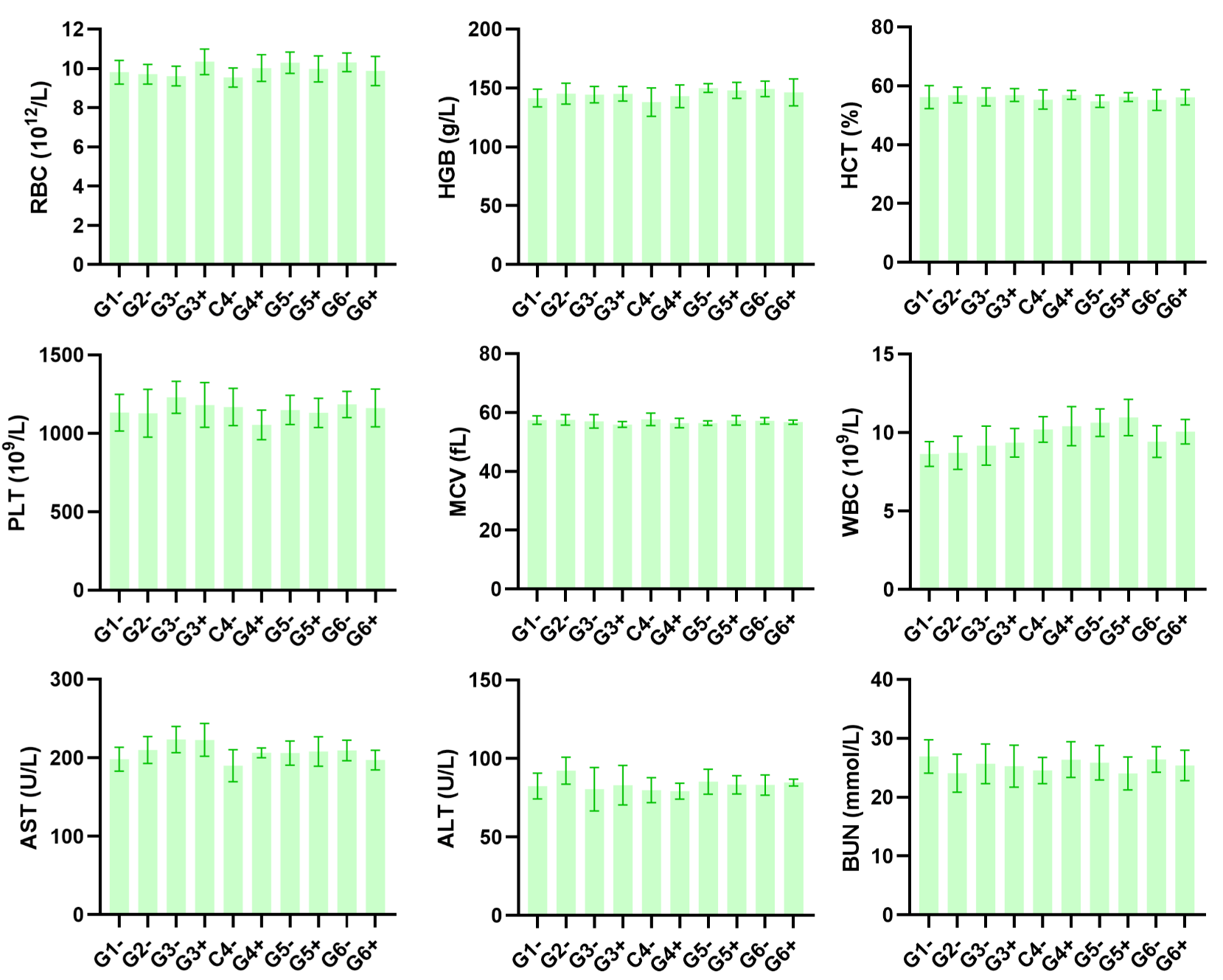


**Fig. S37.** Hematological and serum biochemical parameters of mice from different treatment groups, including complete blood counts: red blood cells (RBC), hemoglobin (HGB), hematocrit (HCT), platelets (PLT), mean corpuscular volume (MCV), and white blood cells (WBC); liver function markers: aspartate aminotransferase (AST), alanine aminotransferase (ALT); serum biochemistry blood urea nitrogen (BUN). G1: PBS, G2: cGAMP, G3: GIP, G4: GIP@M, G5: GIP@CRTM, G6: IP@CRTM. (+): tumors with NIR irradiation (808 nm, 0.5 W/cm^2^, 10 min), (-): tumors without NIR irradiation. Data were presented as mean ± SD (n=5).


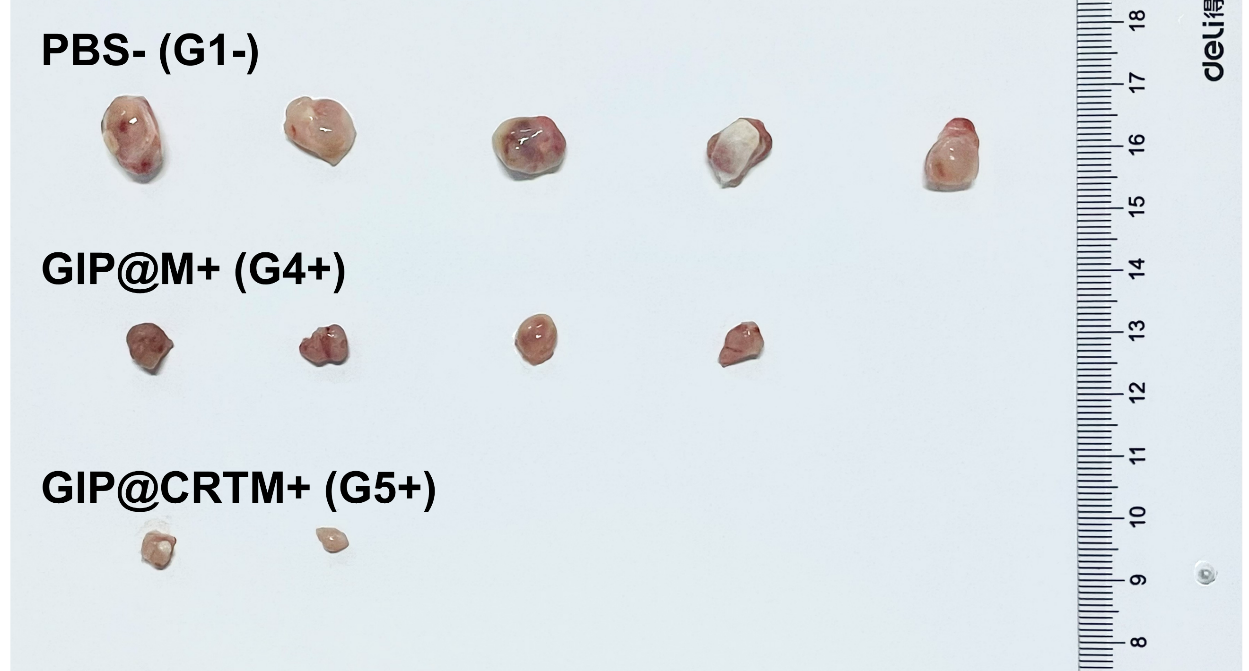


**Fig. S38.** Photograph of postoperative contralateral rechallenge tumors excised from the left flank on day 30 after treatment. (n = 5)


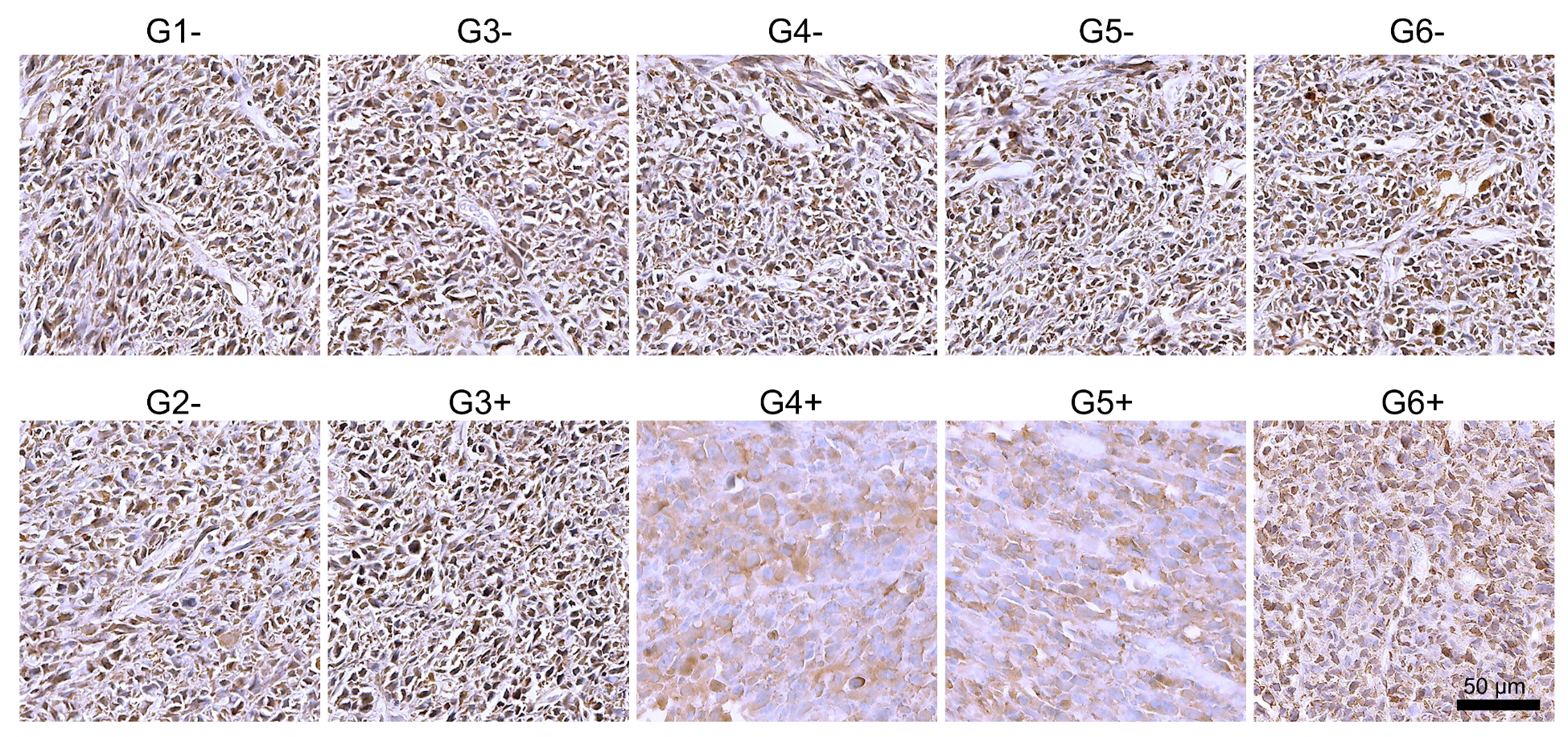


**Fig. S39.** IHC staining of HMGB1 in tumor tissues after different treatments. G1: PBS, G2: cGAMP, G3: GIP, G4: GIP@M, G5: GIP@CRTM, G6: IP@CRTM. (+): tumors with NIR irradiation (808 nm, 0.5 W/cm^2^, 10 min), (-): tumors without NIR irradiation. Scale bar: 50 μm.


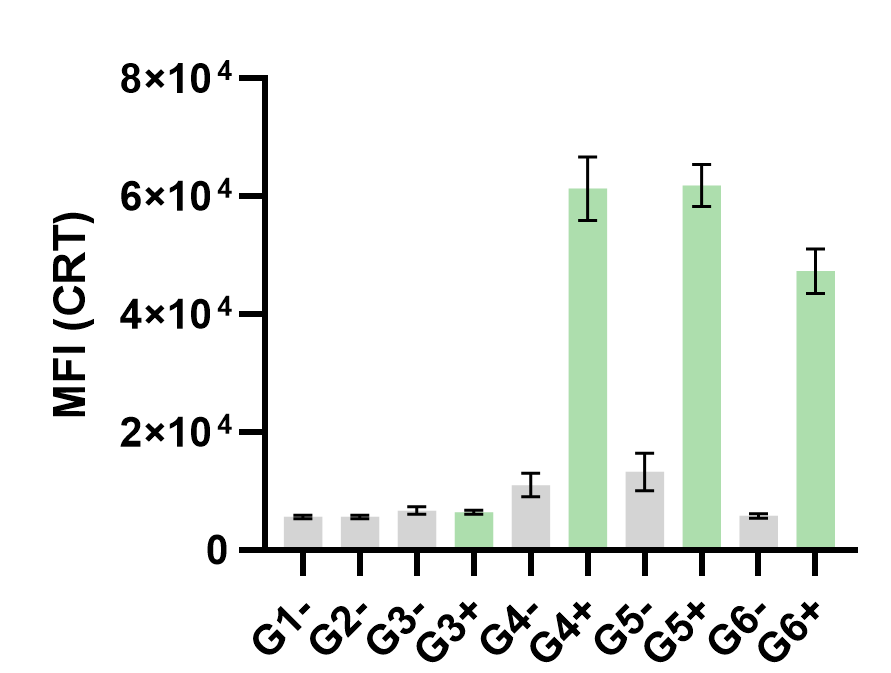


**Fig. S40.** Flow cytometry of surface CRT expression in tumor single-cell suspensions from different treatment groups. G1: PBS, G2: cGAMP, G3: GIP, G4: GIP@M, G5: GIP@CRTM, G6: IP@CRTM. (+): tumors with NIR irradiation (808 nm, 0.5 W/cm^2^, 10 min), (-): tumors without NIR irradiation. Data were presented as mean ± SD (n=5).


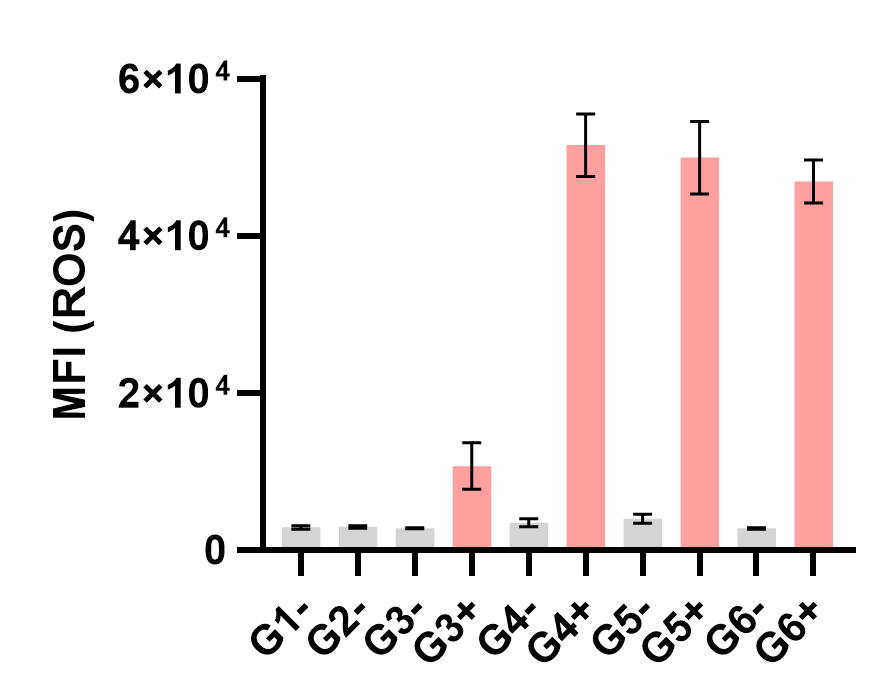


**Fig. S41.** Flow cytometry of ROS levels in tumor single-cell suspensions. G1: PBS, G2: cGAMP, G3: GIP, G4: GIP@M, G5: GIP@CRTM, G6: IP@CRTM. (+): tumors with NIR irradiation (808 nm, 0.5 W/cm^2^, 10 min), (-): tumors without NIR irradiation. Data were presented as mean ± SD (n=5).


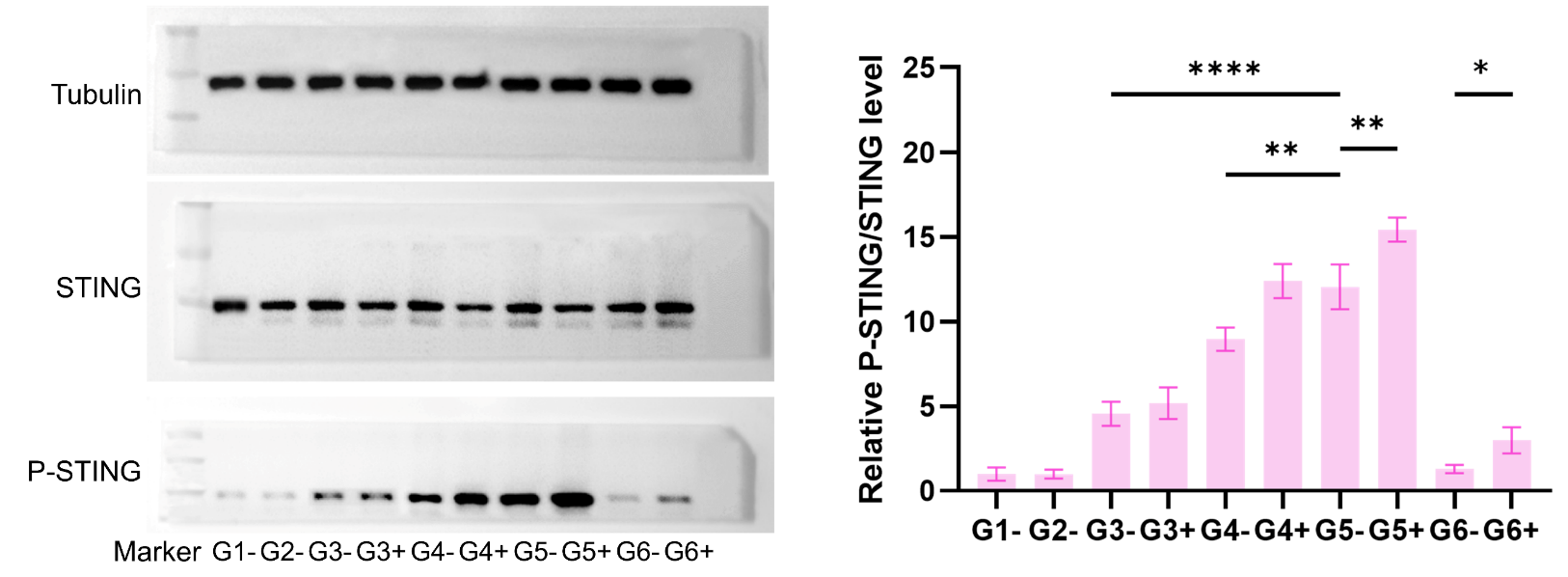


**Fig. S42.** Original uncropped Western blot images and semi-quantitative analysis corresponding to Fig. 7e. Data are presented as mean ± SD (n=5). **p* < 0.1; ***p* < 0.01; ****p* < 0.001; *****p* < 0.0001.


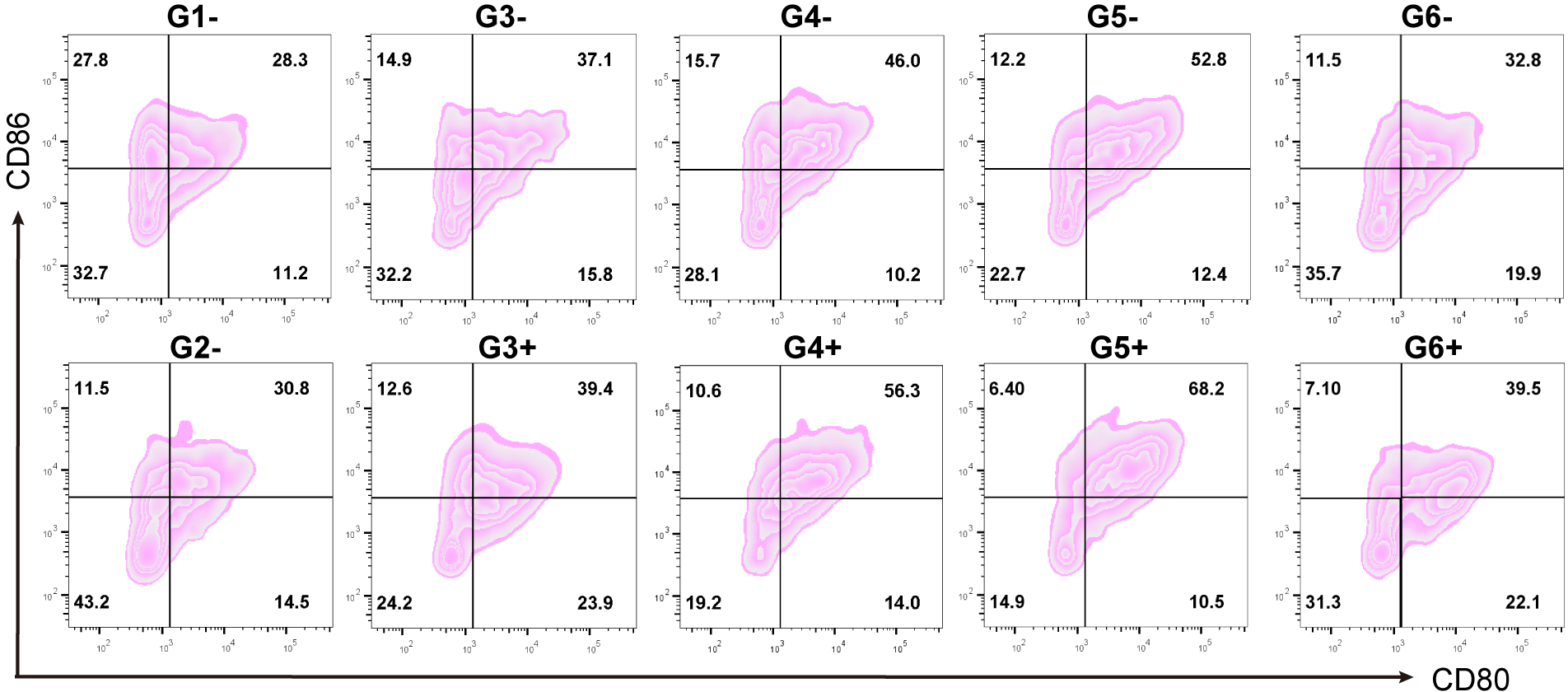


**Fig. S43. Representative flow cytometry plots of mature DCs isolated from tumor tissues in different treatment groups. Mature DCs were identified as CD45⁺CD11c⁺CD80⁺CD86⁺ cells. G1: PBS, G2: cGAMP, G3: GIP, G4: GIP@M, G5: GIP@CRTM, G6: IP@CRTM. (+): tumors with NIR irradiation (808 nm, 0.5 W/cm², 10 min), (-): tumors without NIR irradiation.**


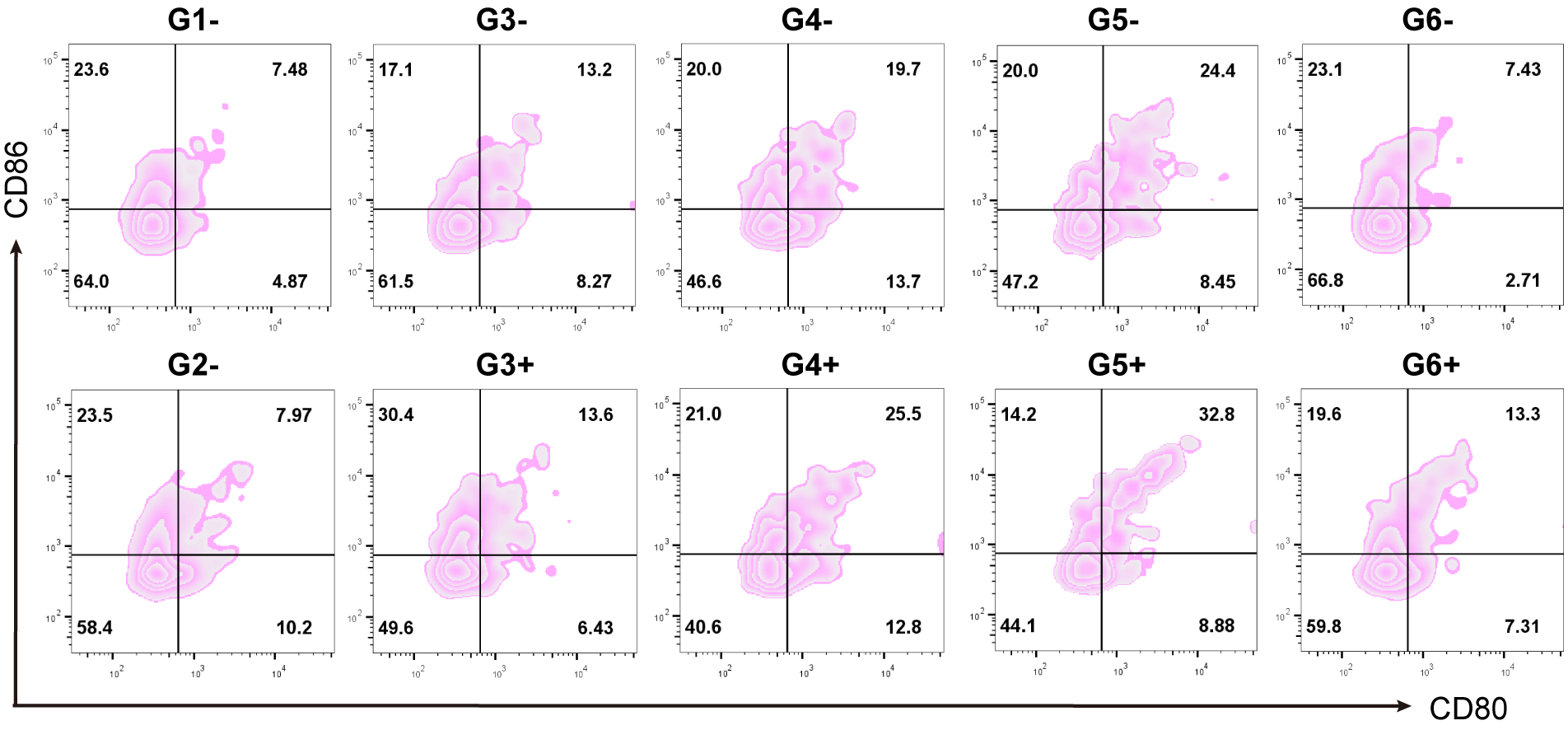


**Fig. S44.** Representative flow cytometric plots of mature DCs (CD45^+^CD11c^+^CD80^+^CD86^+^) in draining lymph nodes from different groups. G1: PBS, G2: cGAMP, G3: GIP, G4: GIP@M, G5: GIP@CRTM, G6: IP@CRTM. (+): tumors with NIR irradiation (808 nm, 0.5 W/cm^2^, 10 min), (-): tumors without NIR irradiation.


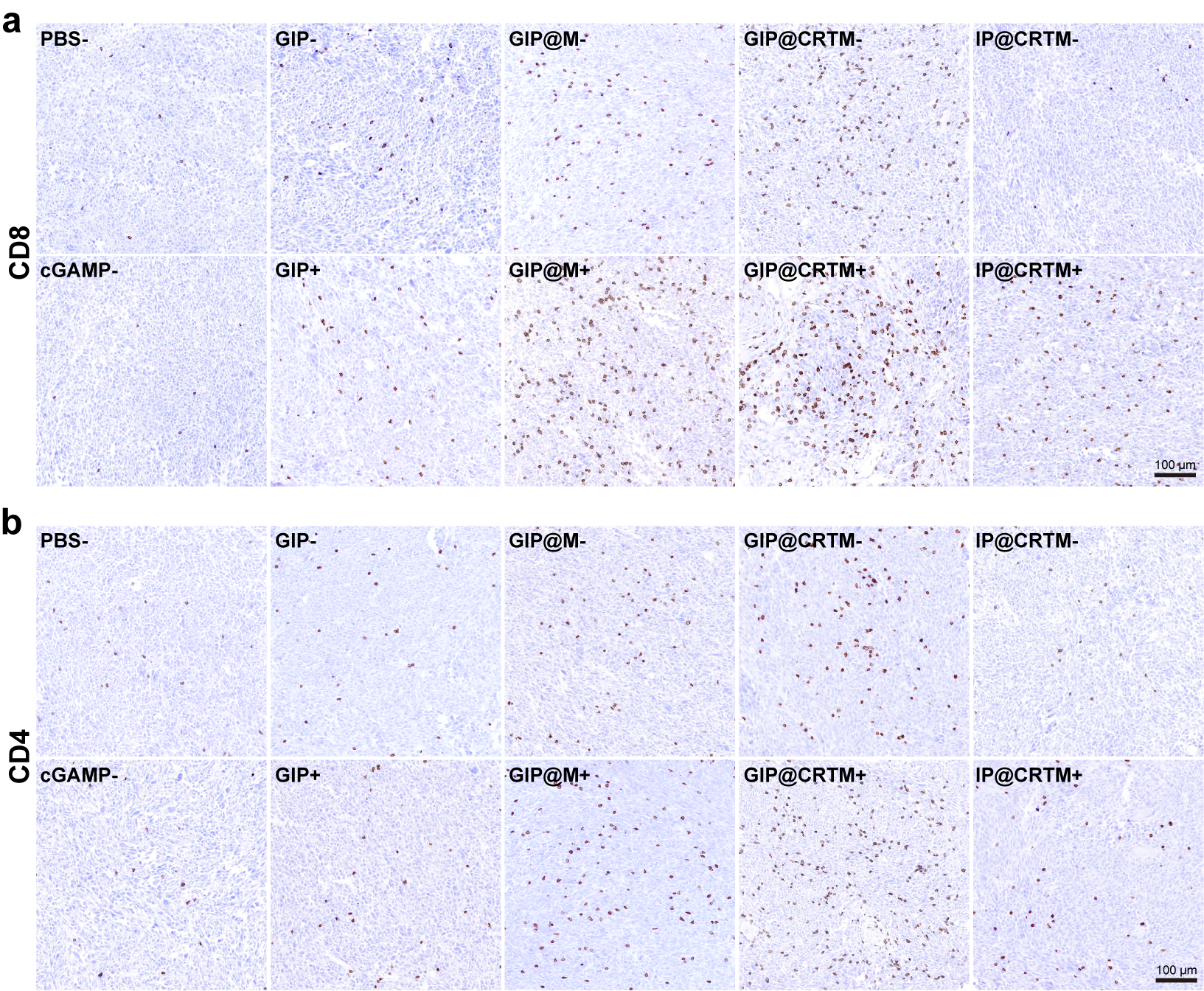


**Fig. S45.** Representative immunohistochemical staining of CD8⁺ (a) and CD4⁺ (b) T cell infiltration in tumor tissues across treatment groups. Scale bar = 100 μm. (+): tumors with NIR irradiation (808 nm, 0.5 W/cm^2^, 10 min), (-): tumors without NIR irradiation


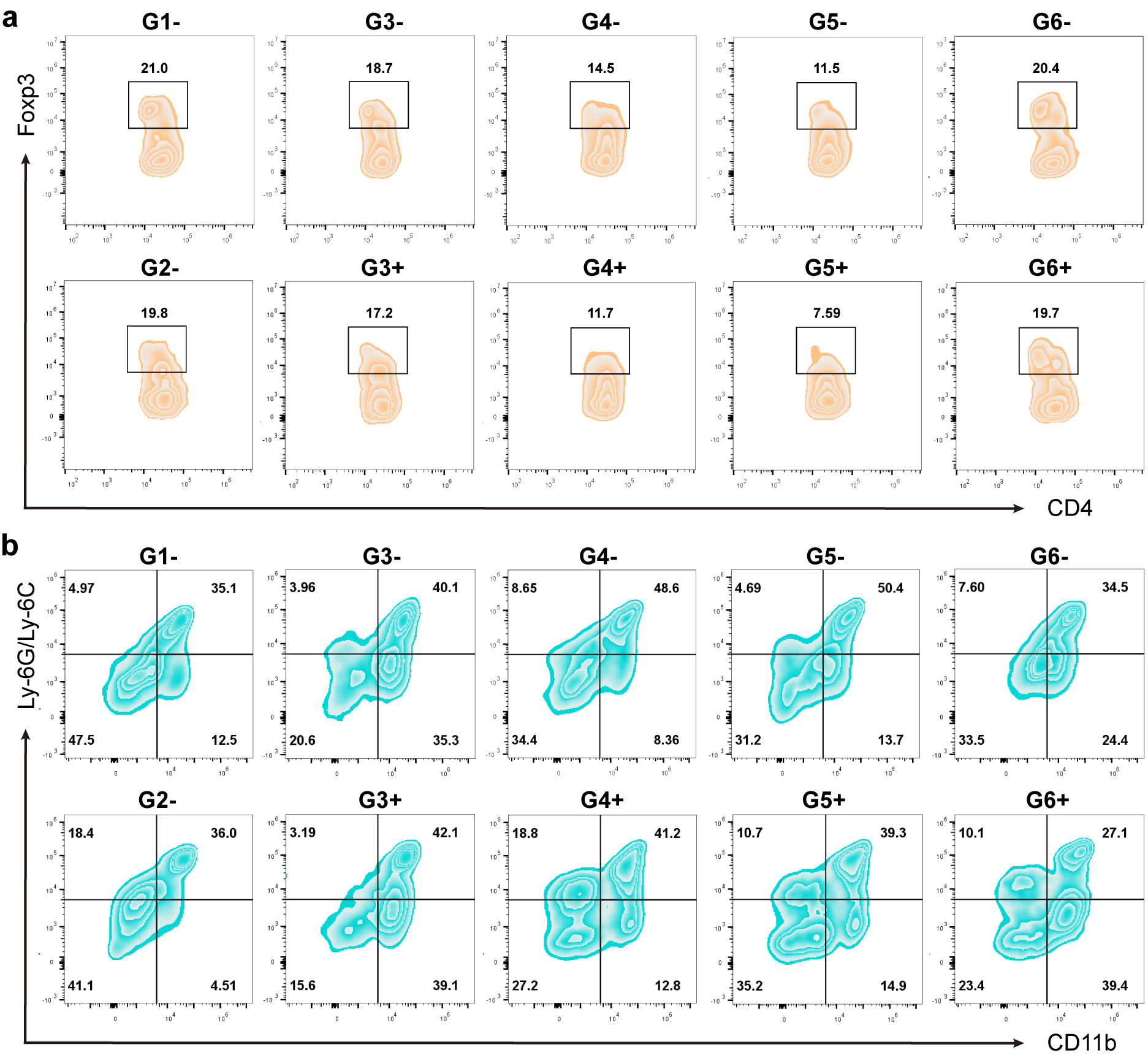


**Fig. S46.** Flow cytometry plots of intratumoral Tregs (CD45^+^CD3^+^CD4^+^Foxp3^+^) and MDSCs (CD45^+^CD11b^+^Ly-6G/Ly-6C^+^). G1: PBS, G2: cGAMP, G3: GIP, G4: GIP@M, G5: GIP@CRTM, G6: IP@CRTM. (+): tumors with NIR irradiation (808 nm, 0.5 W/cm^2^, 10 min), (-): tumors without NIR irradiation.


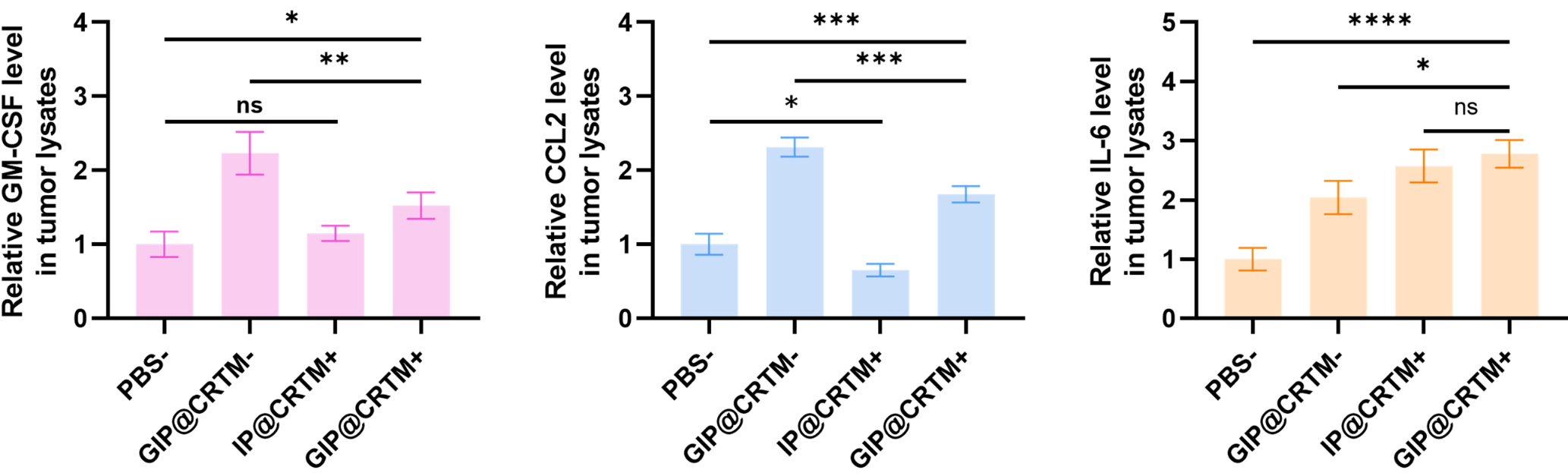


**Fig. S47.** Relative levels of GM-CSF, CCL2, and IL-6 in tumor lysates after different treatments. (+): tumors with NIR irradiation (808 nm, 0.5 W/cm^2^, 10 min), (-): tumors without NIR irradiation. Data are presented as mean ± SD (n=5). ns, not significant; **p* < 0.05; ***p* < 0.01; ****p* < 0.001; *****p* < 0.0001.


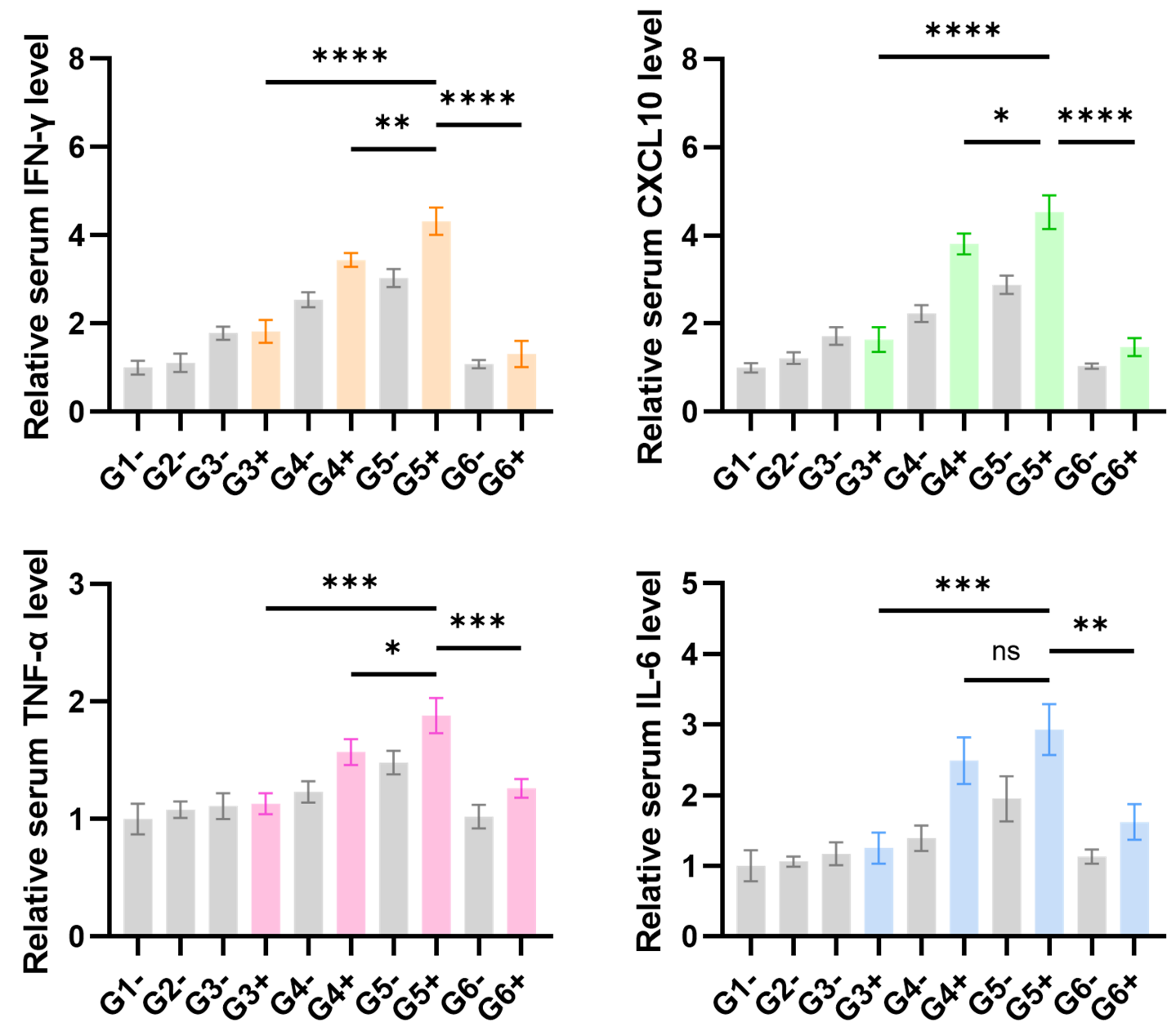


**Fig. S48.** Relative serum levels of immune-related cytokines and chemokines after different treatments. G1: PBS, G2: cGAMP, G3: GIP, G4: GIP@M, G5: GIP@CRTM, G6: IP@CRTM. (+): tumors with NIR irradiation (808 nm, 0.5 W/cm^2^, 10 min), (-): tumors without NIR irradiation. Values were expressed as mean ± SD (n = 5). ns, *p* > 0.05; **p* < 0.05; ***p* < 0.01; ****p* < 0.001; *****p* < 0.0001.


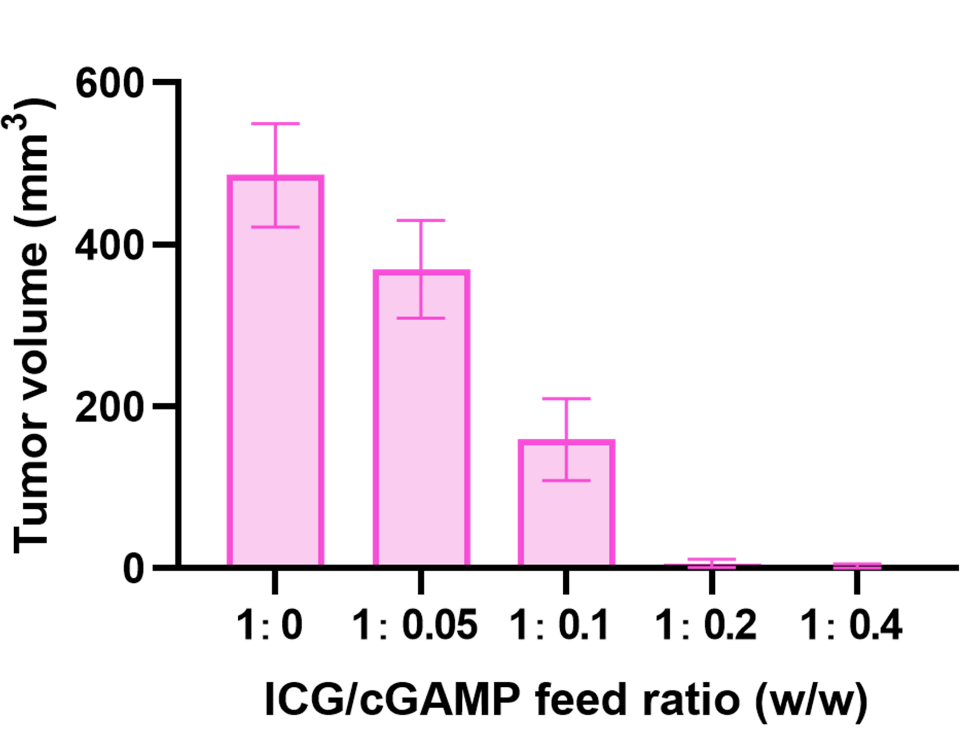


**Fig. S49.** Tumor volume after treatment with GIP@CRTM prepared at different ICG/cGAMP feeding ratios. Data are presented as mean ± SD (n=5).


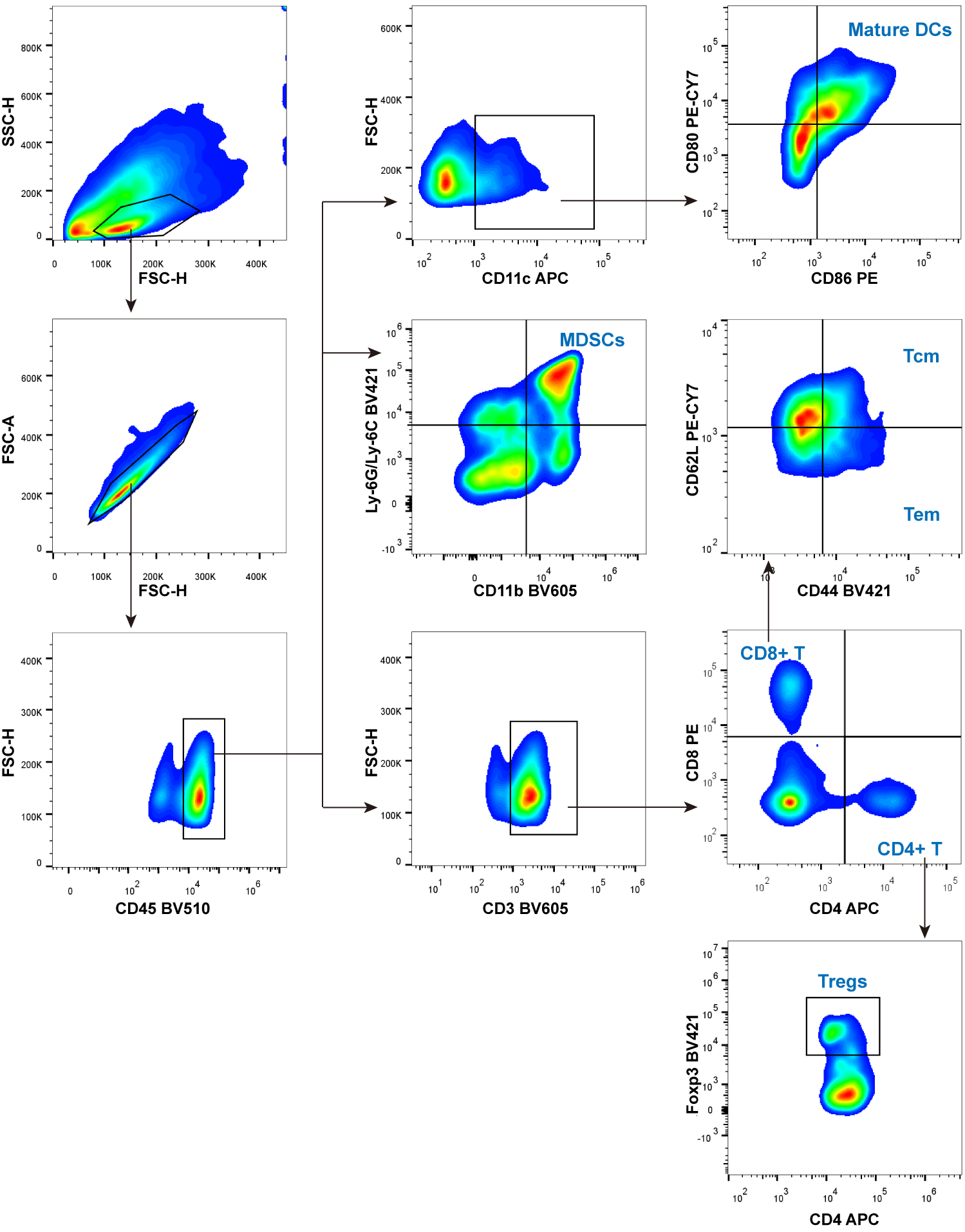


**Fig. S50.** Gating strategies for flow cytometry analysis of mature DCs (CD45^+^CD11c^+^CD80^+^CD86^+^), MDSCs (CD45^+^CD11b^+^Ly-6G/Ly-6C^+^), CD4^+^ T cells (CD45^+^CD3^+^CD4^+^), Tregs (CD45^+^CD3^+^CD4^+^Foxp3^+^), CD8^+^ T cells (CD45^+^CD3^+^CD8^+^), Tcm (CD45^+^CD3^+^CD8^+^CD44^+^CD62L^+^) and Tem (CD45^+^CD3^+^CD8^+^ CD44^+^CD62L^-^) in vivo.
